# Transcriptional Profiling Reveals Kidney Neutrophil Heterogeneity in Both Healthy People and ccRCC Patients

**DOI:** 10.1155/2021/5598627

**Published:** 2021-03-15

**Authors:** Yiliang Meng, Kai Cai, Jingjie Zhao, Keyu Huang, Xiumei Ma, Jian Song, Yunguang Liu

**Affiliations:** ^1^Department of Oncology, People's Hospital of Baise, Baise City, Guangxi Province, China; ^2^Department of Oncology, The Affiliated Southwest Hospital of Youjiang Medical University for Nationalities, Baise City, Guangxi Province, China; ^3^Radiation Therapy Center, The First Affiliated Hospital of Guangxi University of Chinese Medicine, Guangxi Province, China; ^4^Life Science and Clinical Research Center, The Affiliated Hospital of Youjiang Medical University for Nationalities, Baise City, Guangxi Province, China; ^5^Department of Hepatobiliary Surgery, The First Affiliated Hospital of Guangxi Medical University, Nanning City, Guangxi Province, China; ^6^Department of Radiation Oncology, Renji Hospital, School of Medicine, Shanghai Jiao Tong University, Shanghai, China; ^7^Insitute of Physiological Chemistry and Pathobiochemistry, University of Muenster, Germany; ^8^Department of Pediatrics, The Affiliated Hospital of Youjiang Medical University for Nationalities, Baise City, Guangxi Province, China

## Abstract

Neutrophil is known to critically impact the development of renal diseases (e.g., the clear cell renal cell carcinoma (ccRCC)), whereas the heterogeneity of neutrophils in ccRCC remains unclear. In the present study, kidney biopsies from healthy donors and ccRCC tissues were collected for single-cell RNA sequencing (scRNA-seq). In addition, the subpopulations of neutrophils in a healthy kidney and in the tumor microenvironment (TME) of ccRCC were expressed and then analyzed. The genes reported previously were mapped to all subpopulations identified here. On that basis, biological theme comparison and Gene Set Enrichment Analysis (GSEA) were employed to reveal and compare relevant biological functions. In a healthy kidney, neutrophils exhibit two subpopulations: one is more associated with renal autoimmunity, probably acting as therapeutic target; the other is suggested to resist infectious microorganisms. It is noteworthy that six subpopulations were identified in ccRCC biopsy, and two were more relevant to autoimmunity, while the other four are more relevant to the tumor pathology. Besides, ccRCC neutrophil could resist anticancer immune therapies of ipilimumab and pembrolizumab for their low/no expressions of CTLA-4, PD-1, and PD-L1. Thus, this study can help understand the heterogeneity and pathological significance of neutrophils in renal diseases.

## 1. Introduction

Neutrophil heterogeneity refers to an emerging fantastic topic. As revealed from recent studies, neutrophils are highly heterogeneous varying from tissues and pathological conditions [1, 2]. In addition, neutrophils are known to exhibit different functions to tumor development, which may impact the therapeutic efficiency against cancer [3, 4]. To identify neutrophil subpopulations and related functions, especially in a range of tumors, further efforts are required.

As revealed from recent studies, neutrophils critically impact the development of renal carcinoma (e.g., ccRCC) [5–7]. However, the diverse characters exhibited by different subpopulations have been extensively underestimated, mainly because the frequency of neutrophils in the kidney is extremely low and hinders the identification of its subpopulations [8]. The existing advantage of scRNA-seq facilitates the high-throughput analysis of thousands of genes at a single-cell level, so exploring kidney neutrophil heterogeneity under either healthy condition or ccRCC biopsies turns out to be practical in an extremely specific manner.

In this study, the scRNA-seq data acquired from the healthy kidney and ccRCC biopsies were investigated, and the transcriptome of renal neutrophils was profiled. In a healthy kidney, 2 subpopulations were identified. As indicated from the enriched gene profile, one type was more related to autoimmune diseases (e.g., IgA glomerulonephritis, dermatitis, and rheumatoid arthritis), while the other type is more related to infectious disease (e.g., Epstein-Barr virus infection, dengue fever, and Salmonella infections). In the ccRCC biopsy, however, considerable neutrophil was observed. On that basis, a total of 6 subclasses were identified. Note that the biological/pathological effects played by neutrophil subclasses that were identified in ccRCC biopsy overlapped with each other, so they are hard to term for the controversial contributions to a range of diseases (e.g., various tumors, infectious diseases, and autoimmune diseases).

## 2. Methods

### 2.1. scRNA-Seq Analysis

Raw data of healthy kidney biopsies (GSE131685) were offered from Liao et al. [9]. In addition, raw data of ccRCC biopsies (GSE121636) were retrieved from the NCBI GEO database. Though the mentioned two datasets were generated from different machines/platforms, both were acquired with 10x Genomics technology. R package Seurat (version 3.2.1) was employed to process scRNA-seq data [9–11]. SCTransform wrapper was adopted to remove technical variations and confounding mitochondrial genes during data normalization. Moreover, cell cycle phase scores were determined to mitigate the effect of cell cycle heterogeneity. R package scCATCH (version 2.1) was used to annotate cell clusters by complying with NCBI Gene symbols [12]. The healthy kidney and ccRCC data were analyzed, respectively, and then integrated with reciprocal PCA based on conserved genes expressed in both groups. Next, all the clusters were presented by unified manifold approximation and projection (UMAP) with a resolution of 0.6. Furthermore, violin plots and feature plots were adopted to exhibit the expression pattern of genes studied in this project.

### 2.2. Functional Enrichment Analysis [13]

Gene Set Enrichment Analysis (GSEA) was conducted to identify gene sets overrepresented in the respective cluster. The R package clusterProfiler (version 4.0) was used to analyze and visualize functional profiles (GO and KEGG) of enriched gene sets [14]. A *P* value < 0.05 showed statistical significance.

## 3. Results

### 3.1. Neutrophil Heterogeneity in Healthy Kidney

**Figure 1:**
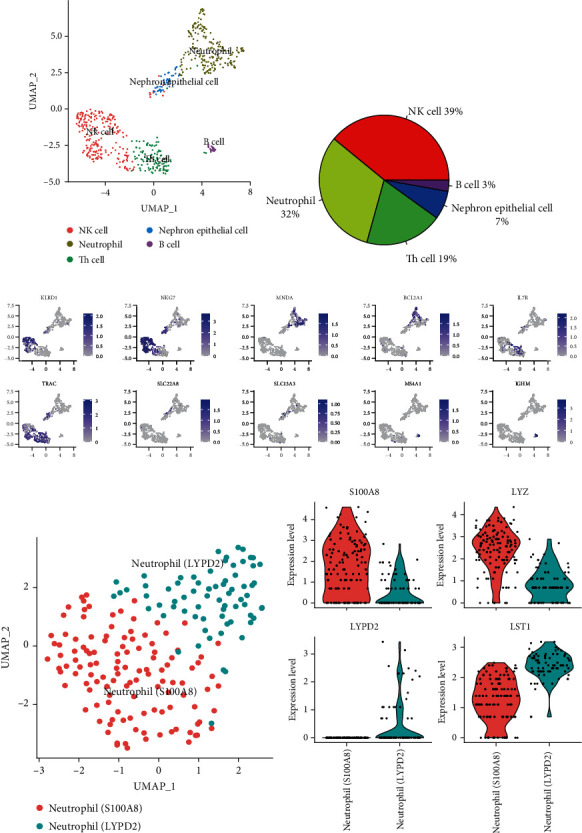
scRNA-seq analysis of CD45+ cells in healthy kidney. (a) UMAP plot shows clustering results from CD45+ cells from healthy kidney. They are NK cells, neutrophils, Th cell, nephron epithelial cell, and B cells (dot size at 0.5). (b) Pie plot indicates the frequency of each cell type. (c) Feature plots show the expression of known markers for each cluster. (d) UMAP plot shows 2 subclusters of neutrophils in healthy kidney (dot size at 4). (e) Violin plots show the differential expression levels of selected 2 genes from each neutrophil subcluster.

Healthy kidney biopsies were donated by 3 patients after radical nephrectomy [9, 13], and transcriptome data were acquired with 10x Genomics Chromium technology [9]. Clustering analysis was conducted with cells expressing PTPRC, a gene coding CD45 (Figure 1(a)). Through clustering analysis, 5 cell types were generated. After revising gene symbols by complying with NCBI Gene symbols, the mentioned 5 cell clusters were annotated as NK cells (cell type score 0.58), neutrophils (cell type score 0.67), Th cells (cell type score 0.63), nephron epithelial cells (cell type score 0.71), and B cells (cell type score 0.86), respectively. Besides, NK cells and neutrophils were reported to be the most abundant ones, taking up 39% and 32% to total CD45+ cells (Figure 1(b)), respectively. Note that nephron epithelial cells were also indicated to be positive for this marker. To test the cell annotation results, marker genes for the mentioned cell types were revealed by the feature plot (Figure 1(c)), i.e., KLRD1 and NKG7 for NK cells, MNDA and BCL2A1 for neutrophils, IL7R and TRAC for Th cells, SLC22A8 and SLC13A3 for nephron epithelial cells, and MS4A1 and IGHM for B cells [15, 16]. It is noteworthy that MNDA and BCL2A1 were expressed in a “compensatory” manner in the cluster of neutrophil, which demonstrated the existence of subpopulations. To test the hypothesis of this study, the whole population of neutrophils was isolated, and cell expressing mitosis genes (i.e., TOP2A or KI67) were regressed to minimize cell cycle effect on identification of neutrophil heterogeneity (Supplementary Fig. 1). After the selection, neutrophils underwent the subclustering, and 2 subpopulations were discovered (Figure 1(d)). To address it in an easy manner, the mentioned 2 neutrophil subpopulations were annotated with neutrophil (S100A8) and neutrophil (LYPD2), respectively. As indicated by further analysis, these 2 neutrophil subpopulations expressed different level of genes (e.g., S100A8, LYZ, LYPD2, and LST1) (Figure 1(e)), which demonstrated that these 2 subpopulations could be different in the expressions of various genes.

**Figure 2:**
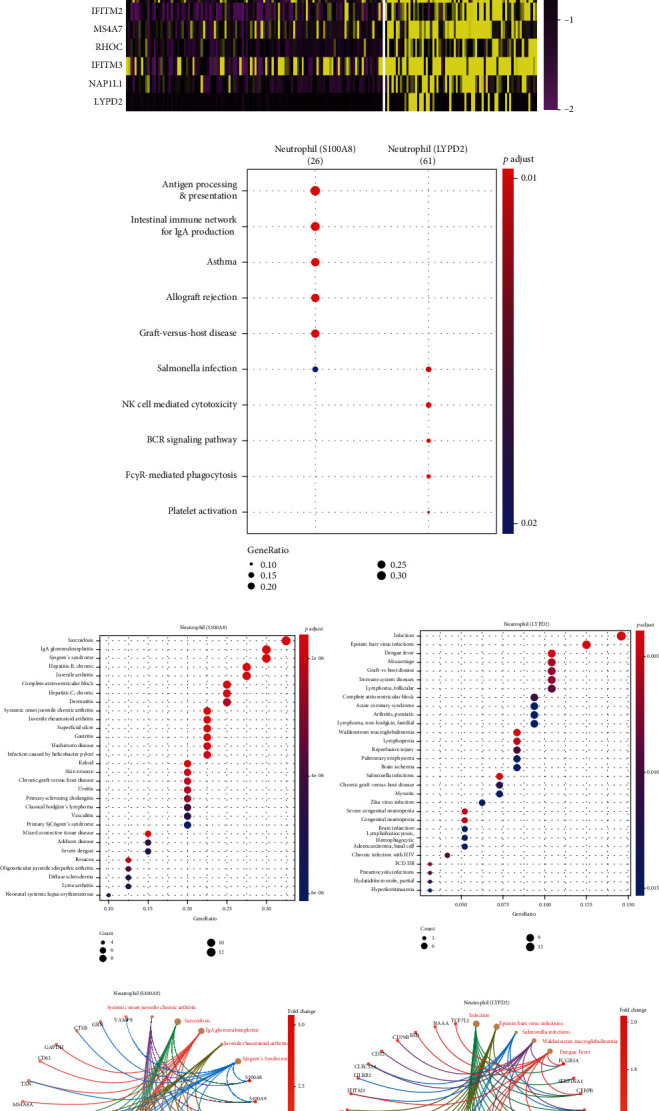
Analysis of healthy kidney neutrophil. (a) Heatmap shows the differential expression levels of top 10 genes selected from each neutrophil subcluster of a healthy kidney. (b) Biological theme comparison of healthy kidney neutrophil subclusters. (c) Overrepresentation analysis (ORA) of each healthy kidney neutrophil subcluster. (d) Cow plots show the gene concept network of each healthy kidney neutrophil subcluster.

To show the disparity in gene expression, 20 genes were taken for comparison. Notably, the mentioned 2 subpopulations displayed different gene profiles (Figure 2(a)), suggesting that they could exhibit different immunological features. Indeed, the gene profile from the first subpopulation was indicated to be more relevant to antigen presentation, in assistance to B cell/plasma cell for IgA production; the gene profile from the second subpopulation was reported to be more associated with BCR signaling and Fc*γ*-mediated phagocytosis (e.g., in assistance to NK cell-mediated cytotoxicity) (Figure 2(b)). Furthermore, these 2 subpopulations exhibited different pathological features. The first subpopulation was indicated to be more related to autoimmune diseases (e.g., IgA glomerulonephritis, juvenile chronic arthritis, rheumatoid arthritis, and sarcoidosis); the second one was more relevant to infectious diseases (e.g., Epstein-Barr virus infection, Salmonella infection, and dengue fever) (Figures 2(c) and 2(d) and Supplementary Fig. 2).

### 3.2. Neutrophil Heterogeneity in ccRCC Biopsies

**Figure 3:**
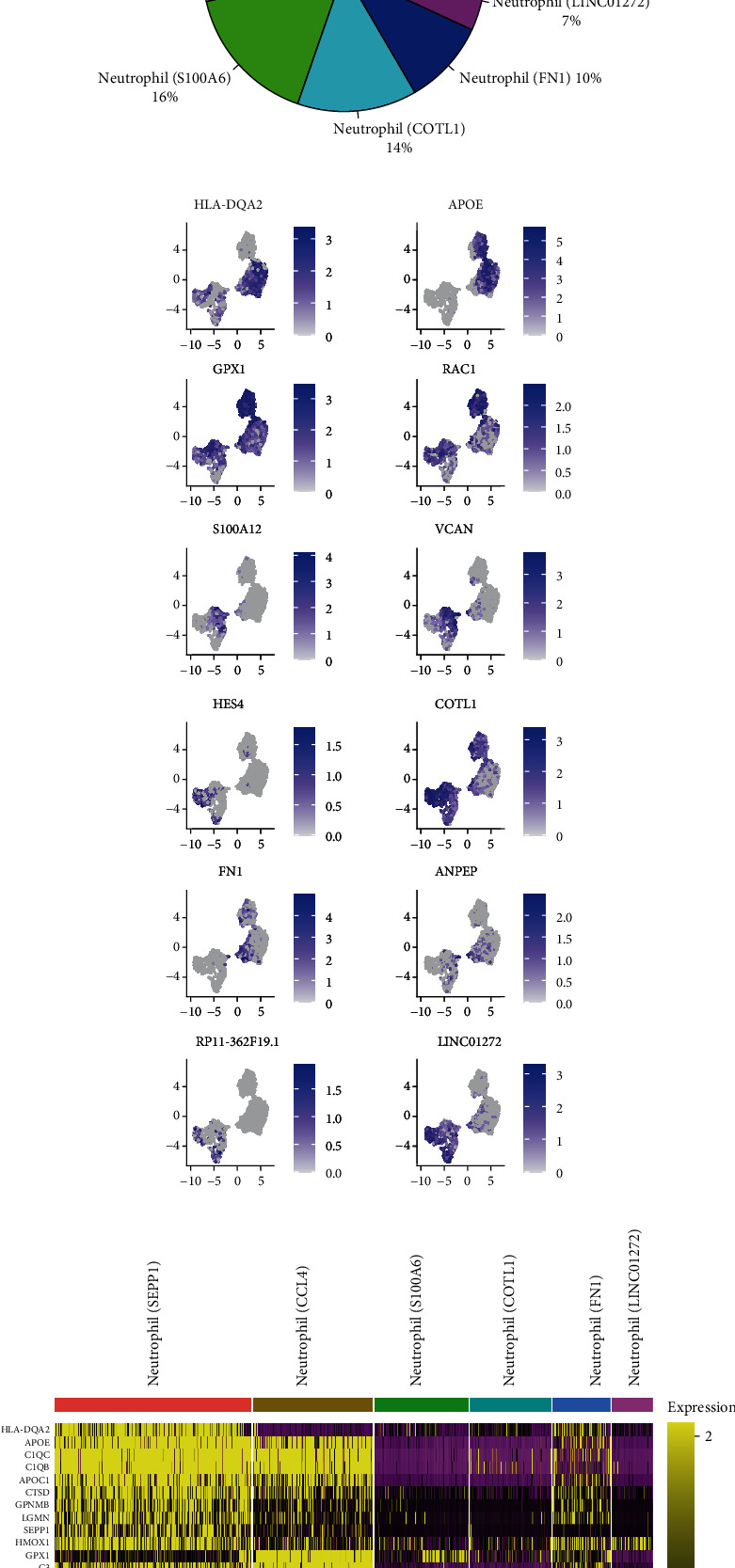
scRNA-seq analysis of neutrophils in ccRCC. (a) UMAP plot shows 6 neutrophil subclusters in ccRCC. (b) Pie plot indicates the frequency of each neutrophil subcluster in ccRCC. (c) Feature plots show the expression of 2 genes selected from each ccRCC neutrophil cluster. (d) Heatmap shows the differential expression levels of top 10 genes selected from each ccRCC neutrophil subcluster.

**Figure 4:**
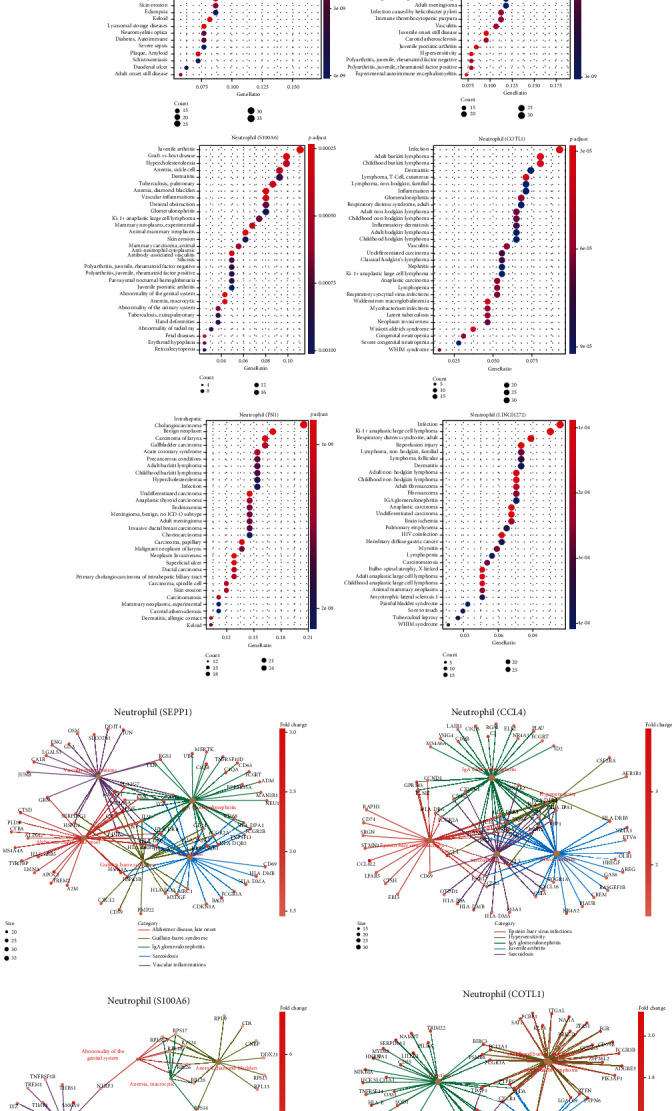
Analysis of ccRCC neutrophils. (a) Biological theme comparison of ccRCC neutrophil subclusters. (b) Overrepresentation analysis (ORA) of each ccRCC neutrophil subcluster. (c) Gene-concept network of each ccRCC neutrophil subcluster.

ccRCC neutrophils were abstracted based on cell annotation by the R package scCATCH [12]. Before the heterogeneity of ccRCC neutrophils was explored, cells expressing either TOP2A or KI67 were deleted (Supplementary Fig. 3). On the whole, 6 subclusters were identified, i.e., neutrophil (SEPP1), neutrophil (CCL4), neutrophil (S100A6), neutrophil (COLT1), neutrophil (FN1), and neutrophil (LINC01272) (Figure 3(a)). To be specific, the biggest part was comprised by neutrophil (SEPP1), while the smallest part was neutrophil (LINC01272) (Figure 3(b)). Next, the expressions of top 2 genes from each subcluster were tested and then compared (Figure 3(c) and Supplementary Fig. 4). As revealed from the analysis of the disparity of top 10 genes selected from each subpopulation, ccRCC neutrophil subclusters exerted overlapped expression of some genes (Figure 3(d)), demonstrating that the mentioned subpopulations could potentially share some immunological/pathological features. The first two ccRCC neutrophil subpopulations showed great similarity in immunological procedures (e.g., antigen presentation, lysosome, and phagosome); the third subpopulation was suggested to critically impact COVID-19 injection and ribosome; the last three subpopulations were significantly more relevant to Salmonella infection (Figure 4(a)). Furthermore, the first two subpopulations were noticeably more relevant to autoimmune diseases (e.g., IgA glomerulonephritis, Alzheimer disease, Sarcoidosis, hypersensitivity, and arthritis). Such a pathological feature made these two populations parallel with the first neutrophil subpopulation identified in a healthy kidney. However, these two subpopulations were also suggested to be related to microbial infections to a certain extent (Figures 4(b) and 4(c) and Supplementary Fig. 5). The last four populations, however, were reported to be mainly relevant to tumor development: neutrophil (S100A6) for mammary neoplasm; neutrophil (COTL1) for Burkitt lymphoma and cutaneous T cell lymphoma; neutrophil (FN1) for invasive neoplasm, papillary carcinoma, undifferentiated carcinoma, etc.; neutrophil (LINC01272) for Ki-1+ anaplastic large cell lymphoma and anaplastic carcinoma (Figures 4(b) and 4(c) and Supplementary Fig. 5). Note that the fourth and sixth subpopulations were relevant to infectious diseases as well, consistent with the second neutrophil subpopulation identified in a healthy kidney.

### 3.3. Comparison of Kidney Neutrophil Subpopulations between Healthy and ccRCC Biopsies

**Figure 5:**
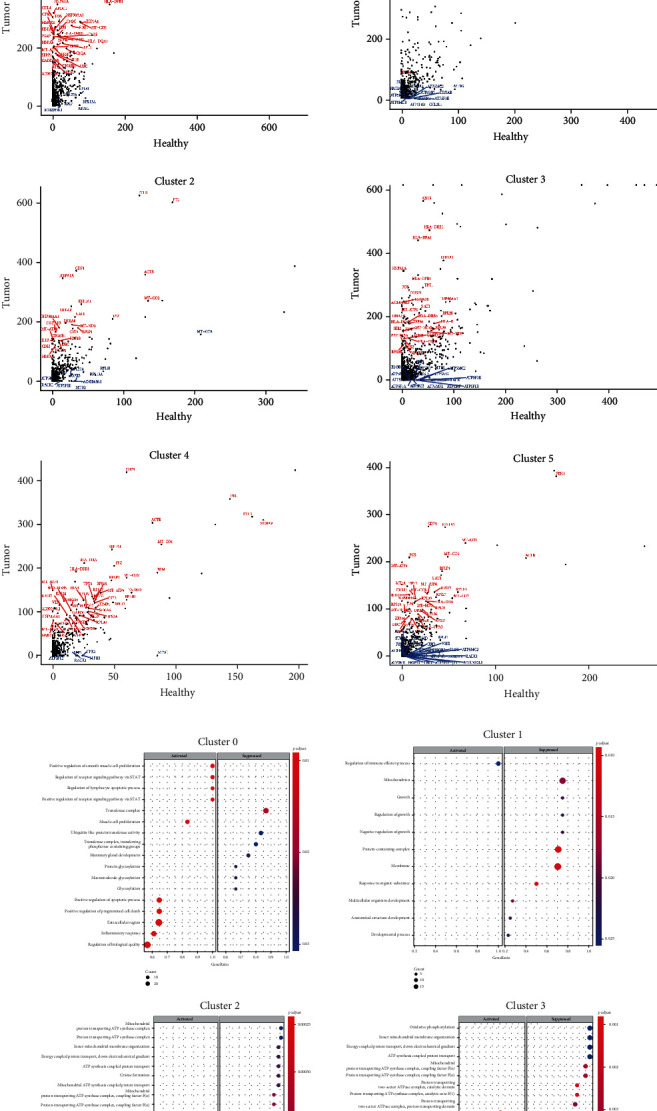
Analysis of integrated neutrophil of healthy kidney and ccRCC. (a) UMAP plot shows “Merged” healthy kidney neutrophils and ccRCC neutrophils based on all the genes detected. (b) UMAP plot shows 6 neutrophil subclusters of integrated neutrophils based on conserved genes. (c) Dot plots show the differential expression levels of the most significant genes for each cluster. (d) GSEA analysis of each integrated neutrophil subcluster. (e) Expression levels of CTLA-1, PD-1, and PD-L1 in ccRCC neutrophils.

To compare the healthy kidney neutrophils and ccRCC neutrophils, these neutrophils were first “merged” (Figure 5(a)), with which the Seurat package will evaluate all the genes. The UMAP plot showed significant different difference between these neutrophils, demonstrating that ccRCC neutrophils are highly heterogeneous. Afterwards, these neutrophils were “integrated” based on those conserved genes expressed by both ones. Next, 6 subpopulations were identified (Figure 5(b)). A more considerable number of neutrophils were observed in ccRCC than those in a healthy kidney. As revealed from the comparison, most DEGs, in ccRCC neutrophils, were expressed in a much higher level than in healthy ones (Figure 5(c), Supplementary Figure 6), revealing that ccRCC neutrophils are more active in the microenvironment of tumors. To verify this finding, the above enriched genes were placed into KEGG and GO analyses by clusters. In clusters 0 and 1, neutrophils exerted activated signaling in a positive regulation of apoptotic, programmed cell death and stronger inflammatory response, and suppressed activity in transferase, mitochondrion and membrane maintenance, which demonstrated that the mentioned subpopulations could be antitumor ones. Clusters 2, 3, 4, and 5 showed the upregulation in metabolic, catabolic, and lytic vacuole; cell communication; interaction with organisms; and cellular biosynthetic processes, while the downregulation was identified in ATP synthesis and proton-transporting ATP synthase complex. Such an immunological feature indicated that these could be more tumor-like cells and be more effective in modulation of tumor environments (Figure 5(d)).

### 3.4. ccRCC Neutrophils Could Potentially Resistant to Immune Therapies

Ipilimumab and pembrolizumab have been extensively employed as immune therapies in clinic against tumor, targeting on CTLA-4 and PD-1 signaling, respectively [17, 18]. They are used for advanced renal cell carcinoma [19, 20]. However, how ccRCC neutrophils will react to these two medicines remains unclear. Thus, the expression levels of CTLA4 (coding CTLA-4), PDCD1 (coding PD-1), and PDCD1LG2 (coding PD-L1) were tested. Disappointedly, almost the whole ccRCC neutrophils do not express these three genes (Figure 5(e) and Supplementary Fig. 7), demonstrating that these cells could “escape” from the immune therapy of either ipilimumab or pembrolizumab.

## 4. Discussion

Neutrophil heterogeneity and related immunological/pathological features in the development of renal disease has recently aroused wide attention [21]. It is known that neutrophils exhibit highly different phenotypes and functions mainly depending on tissues and individual conditions [22–25]. Though studies reported neutrophil subpopulation/heterogeneity in the blood, bone marrow, spleen, and even in the brain, lung, and gut [26–28], the kidney neutrophil has been rarely studied for its extremely low frequency in this tissue. This study reported neutrophil heterogeneity in ccRCC, comparing with it in healthy condition, in a single-cell level. On that basis, kidney neutrophil subpopulations in healthy condition and in ccRCC, together with their DEGs and discrete immunological/pathological features, were described.

Existing studies simply classified neutrophils into either antitumor or protumor subpopulations [29, 30], whereas this preliminary nomenclature overshadowed versatile immunological/pathological features of neutrophils. To remedy this defect, the transcriptome scRNA-seq technology provides high-throughput outcome of genes on a single-cell level, thereby making it practical to express and compare the similarity and disparity among thousands of cells, and provides a much better methodology to classify subpopulations in an elegant manner. In this study, two neutrophils were found in a healthy kidney, i.e., one was involved in autoimmunity, and the other was more relevant to the resistant to infectious diseases. The first subpopulation might act as a therapeutic target against autoimmune renal diseases. In ccRCC biopsy, neutrophil exhibited much higher heterogeneity, and 6 subpopulations were finally identified. Besides, since abundant genes were upregulated, immunological/pathological features were overlapped among some subpopulations. Nevertheless, the first two populations were suggested to be more relevant to autoimmune diseases, and the last four subpopulations could potentially participate in the development of cancers and act as therapeutic targets against cancer.

Limitation to this study includes the lacked technology of depleting specific neutrophil subpopulations, which should be addressed in the near future to systematically assess the contribution of each neutrophil subpopulation to the development of renal cancer. Besides, several other techniques could be performed with either healthy kidney or ccRCC biopsies (e.g., immunofluorescent staining and western blot) to validate the discoveries.

## Data Availability

The datasets and code generated or analyzed in this study are available from the corresponding author upon reasonable request.

## Supplementary Materials

Supplementary Materials Supplementary Figure 1: expression levels of TOP2A and KI67 by healthy kidney neutrophils. Feature plots and violin plots show the expression levels of TOP2A and KI67 in healthy kidney neutrophils. Supplementary Figure 2: gene-concept network of healthy kidney neutrophil subpopulations. Heat-like maps show disease-related genes in healthy kidney neutrophil subpopulations. Supplementary Figure 3: expression levels of TOP2A and KI67 by ccRCC neutrophils. Feature plots and violin plots show the expression levels of TOP2A and KI67 in ccRCC neutrophils. Supplementary Figure 4: gene-concept network of ccRCC neutrophil subpopulations. Heat-like maps show disease-related genes in ccRCC neutrophil subpopulations. Supplementary Figure 5: violin plots show the expression levels of top 2 significant genes selected from each ccRCC neutrophil cluster. Supplementary Figure 6: detailed lists of DEGs of integrated neutrophil subpopulations. Supplementary Figure 7: feature plots show the expression levels of CTLA-4, PD-1 and PD-L1 in ccRCC neutrophil cluster.
