# Supplementary material for: Transcriptional Profiling Reveals Kidney Neutrophil Heterogeneity in Both Healthy People and ccRCC Patients
Source: J Immunol Res. 2021 Mar 15;2021:5598627. doi: 10.1155/2021/5598627 (PMC7984911; doi:10.1155/2021/5598627)
Supplement: Supplementary Materials — Supplementary Figure 1: expression levels of TOP2A and KI67 by healthy kidney neutrophils. Feature plots and violin plots show the expression levels of TOP2A and KI67 in healthy kidney neutrophils. Supplementary Figure 2: gene-concept network of healthy kidney neutrophil subpopulations. Heat-like maps show disease-related genes in healthy kidney neutrophil subpopulations. Supplementary Figure 3: expression levels of TOP2A and KI67 by ccRCC neutrophils. Feature plots and violin plots show the expression levels of TOP2A and KI67 in ccRCC neutrophils. Supplementary Figure 4: gene-concept network of ccRCC neutrophil subpopulations. Heat-like maps show disease-related genes in ccRCC neutrophil subpopulations. Supplementary Figure 5: violin plots show the expression levels of top 2 significant genes selected from each ccRCC neutrophil cluster. Supplementary Figure 6: detailed lists of DEGs of integrated neutrophil subpopulations. Supplementary Figure 7: feature plots show the expression levels of CTLA-4, PD-1 and PD-L1 in ccRCC neutrophil cluster. [file 5598627.f1.zip › Supplementary files/Supplementary Figure 6 (1).pdf]

Differntially Expressed Genes (DEGs) of Cluster 4

	p_val	avg_logFC	pct.1	pct.2	p_val_adj
ATP5F1E	9,26E-50	-12,33	0	1	2,21E-45
ATP5MG	4,41E-46	-8,16543	0	0,936	1,05E-41
AC020656.	1,16E-43	-19,1499	0	0,894	2,77E-39
ATP5MC2	1,81E-42	-7,52766	0	0,872	4,32E-38
RACK1	5,91E-39	-20,1499	0	0,809	1,41E-34
MT1G	5,96E-39	-85,1499	0	0,809	1,42E-34
ELOB	1,09E-36	-2,49963	0	0,766	2,61E-32
ATP5MPL	1,50E-35	-3,59401	0	0,745	3,58E-31
ATP5ME	3,13E-32	-3,4588	0	0,681	7,46E-28
ATP5MF	3,78E-31	-1,83171	0	0,66	8,99E-27
ATP5F1D	6,64E-28	-2,13225	0	0,596	1,58E-23
AC090498.	3,45E-26	100,825	1	0	8,21E-22
MT-ATP8	3,46E-26	148,8182	1	0	8,24E-22
ATP5E	5,90E-26	25,21044	0,994	0	1,41E-21
GNB2L1	6,00E-26	67,81822	0,994	0	1,43E-21
FYB1	7,98E-26	-1,34792	0	0,553	1,90E-21
MT-ND4L	8,67E-26	80,04358	1	0,298	2,06E-21
ATP5L	1,58E-25	11,58043	0,983	0	3,76E-21
MT-ND3	2,31E-25	74,35647	1	0,915	5,49E-21
GABARAP	2,69E-25	24,09953	0,983	0,064	6,42E-21
ATP5G2	3,10E-25	20,86787	0,978	0	7,38E-21
GPX1	5,94E-25	46,13153	0,972	0	1,42E-20
ATP5PO	8,49E-25	-1,84391	0	0,532	2,02E-20
MT1H	9,24E-25	-24,1502	0	0,532	2,20E-20
TCEB2	1,18E-24	7,072396	0,961	0	2,82E-20
PABPC1	2,87E-24	30,21553	0,994	0,638	6,84E-20
HLA-B	3,81E-24	66,61242	1	0,936	9,08E-20
RPL17	4,71E-24	44,46062	0,994	0,66	1,12E-19
ALDOA	5,00E-24	17,42624	0,961	0,085	1,19E-19
VIM	5,42E-24	110,1753	1	0,766	1,29E-19
RPL39	1,09E-23	86,32641	1	1	2,60E-19
TPT1	1,55E-23	102,6684	1	0,979	3,70E-19
LAPTM5	2,24E-23	25,4393	0,994	0,702	5,34E-19
RPS24	2,76E-23	88,07448	1	1	6,57E-19
RPS21	2,77E-23	48,58937	0,994	0,809	6,59E-19
RPL37	3,62E-23	81,29024	1	1	8,63E-19
RPL30	3,67E-23	69,67508	1	0,936	8,73E-19
C14orf2	6,26E-23	9,008318	0,921	0	1,49E-18
VSIR	1,00E-22	-1,10041	0	0,489	2,38E-18
MT-CO2	1,45E-22	117,5414	1	1	3,45E-18
HLA-A	1,96E-22	47,47347	1	0,681	4,66E-18
ATP6V0C	2,08E-22	9,50819	0,91	0	4,95E-18
ACTG1	2,34E-22	66,70542	0,994	0,723	5,57E-18
EEF1A1	2,47E-22	194,7953	1	1	5,88E-18
NFKBIA	4,26E-22	64,44765	0,961	0,255	1,01E-17
RNASEK	5,13E-22	6,928242	0,916	0,064	1,22E-17
C10orf54	5,41E-22	8,319861	0,899	0	1,29E-17
FYB	5,67E-22	8,514459	0,899	0	1,35E-17

RPS10	7,20E-22	50,84098	0,989	0,702	1,72E-17
MT-ND5	1,02E-21	42,29101	0,994	0,617	2,42E-17
FOSB	2,46E-21	16,89112	0,933	0,234	5,85E-17
EEF1G	3,28E-21	23,81827	0,882	0	7,81E-17
RPS26	5,17E-21	56,1669	0,966	0,553	1,23E-16
NME2	5,91E-21	12,95005	0,882	0,021	1,41E-16
ACTB	5,92E-21	222,6684	1	1	1,41E-16
ATP5PF	1,02E-20	-1,80427	0	0,447	2,42E-16
SELENOH	1,04E-20	-1,27179	0	0,447	2,48E-16
RPS13	1,07E-20	67,66836	0,994	1	2,55E-16
MT-ND1	2,21E-20	18,66875	1	0,936	5,27E-16
PLAUR	2,47E-20	34,0205	0,955	0,447	5,89E-16
EIF1	2,84E-20	37,64983	1	0,957	6,78E-16
SRGN	3,12E-20	70,66824	1	0,851	7,44E-16
RPS28	3,55E-20	63,35096	1	1	8,46E-16
EEF2	4,53E-20	18,72332	0,955	0,383	1,08E-15
NACA	5,81E-20	31,92341	0,994	0,83	1,38E-15
USMG5	6,11E-20	4,632235	0,843	0	1,46E-15
MT-CO1	6,29E-20	165,3551	1	1	1,50E-15
RPLP1	6,49E-20	123,975	1	1	1,55E-15
PSAP	8,78E-20	47,67781	1	0,894	2,09E-15
MT-ATP6	8,79E-20	104,6189	1	0,957	2,09E-15
MAP3K8	9,52E-20	15,4704	0,865	0,064	2,27E-15
SMIM25	1,05E-19	-1,49704	0	0,426	2,50E-15
CD83	1,12E-19	10,0828	0,865	0,064	2,67E-15
LCP1	1,17E-19	20,74244	0,972	0,447	2,78E-15
RPL29	1,24E-19	47,60036	0,994	0,894	2,94E-15
RPL7A	1,27E-19	37,94349	0,994	0,872	3,03E-15
PPP1R15A	1,41E-19	14,68562	0,899	0,149	3,37E-15
PNRC1	1,59E-19	10,32648	0,933	0,362	3,79E-15
RPS7	2,00E-19	59,61777	0,989	0,915	4,77E-15
LYZ	2,04E-19	154,6682	1	0,979	4,85E-15
RPS12	2,06E-19	80,67474	0,994	1	4,90E-15
RPL18	2,24E-19	35,61864	1	0,936	5,33E-15
GPX3	2,39E-19	-24,0711	0,056	0,596	5,69E-15
RPL28	2,79E-19	60,66849	0,994	1	6,64E-15
RBM39	2,89E-19	4,324782	0,916	0,255	6,89E-15
ARPC1B	3,12E-19	22,02616	0,972	0,638	7,43E-15
MCL1	4,63E-19	16,65951	0,978	0,66	1,10E-14
FXYD5	4,92E-19	11,00363	0,966	0,489	1,17E-14
ATP5I	7,01E-19	6,269679	0,815	0	1,67E-14
BCL2A1	7,48E-19	18,52639	0,893	0,255	1,78E-14
NR4A1	8,89E-19	11,34847	0,843	0,064	2,12E-14
FAU	9,26E-19	36,53493	0,994	0,957	2,21E-14
ARHGDIB	9,59E-19	13,82312	0,944	0,447	2,28E-14
ZFP36	9,73E-19	72,66832	0,989	0,702	2,32E-14
COPS9	9,90E-19	-0,90083	0	0,404	2,36E-14
NAMPT	1,14E-18	16,70089	0,972	0,723	2,72E-14
CD74	1,20E-18	358,6684	0,994	0,83	2,85E-14
HLA-DRB5	1,88E-18	115,6655	0,944	0,298	4,47E-14

ANXA5	2,23E-18	9,169522	0,949	0,362	5,31E-14
SLC11A1	2,25E-18	9,119281	0,904	0,213	5,36E-14
HLA-C	2,36E-18	38,97498	0,994	0,894	5,63E-14
GAPDH	2,46E-18	76,6464	0,989	0,979	5,85E-14
ATP5G3	2,52E-18	9,865509	0,803	0	6,01E-14
CD44	3,27E-18	13,41402	0,955	0,511	7,78E-14
GPCPD1	3,51E-18	6,056224	0,831	0,064	8,36E-14
RPL24	5,57E-18	25,34783	1	0,83	1,33E-13
CLEC7A	7,54E-18	6,521142	0,888	0,234	1,80E-13
LINC01272	9,03E-18	8,311608	0,787	0	2,15E-13
TNFAIP3	9,28E-18	28,74767	0,809	0,043	2,21E-13
SMIM26	9,32E-18	-0,671	0	0,383	2,22E-13
ATP5MD	9,32E-18	-1,01762	0	0,383	2,22E-13
ATP5PB	9,41E-18	-0,84403	0	0,383	2,24E-13
BTG1	9,73E-18	39,6803	0,966	0,532	2,32E-13
RPL26	1,02E-17	54,66744	0,994	0,979	2,43E-13
RPLP0	1,04E-17	57,95749	0,983	0,787	2,47E-13
PPIA	1,09E-17	27,10378	0,989	0,723	2,60E-13
RHOA	1,11E-17	18,36973	0,972	0,532	2,64E-13
RPS8	1,18E-17	101,6673	0,994	0,979	2,80E-13
RPL19	1,22E-17	82,54142	1	0,979	2,92E-13
CFL1	1,28E-17	26,53194	0,994	0,851	3,06E-13
SH3BGRL3	1,50E-17	49,61931	0,994	0,979	3,58E-13
ATP5H	1,58E-17	2,947266	0,775	0	3,76E-13
ANXA1	1,62E-17	38,66071	0,916	0,34	3,86E-13
DDX5	1,66E-17	10,46986	0,966	0,617	3,94E-13
CD68	1,67E-17	9,840679	0,944	0,383	3,98E-13
S100A6	1,82E-17	78,53446	1	0,979	4,33E-13
NBEAL1	2,02E-17	4,831383	0,831	0,106	4,81E-13
ATP5J2	2,05E-17	6,19983	0,775	0	4,89E-13
C19orf43	2,21E-17	3,143235	0,77	0	5,26E-13
SOD2	3,26E-17	24,03662	0,91	0,426	7,77E-13
RPL35A	3,44E-17	34,92274	0,994	0,979	8,19E-13
HSP90AA1	3,80E-17	92,65453	0,983	0,447	9,06E-13
IL1B	4,43E-17	63,79838	0,904	0,298	1,05E-12
LSP1	4,49E-17	17,88169	0,949	0,404	1,07E-12
MAP1LC3B	4,49E-17	5,280984	0,815	0,085	1,07E-12
TSPO	5,18E-17	12,24898	0,978	0,809	1,23E-12
RPL6	6,34E-17	33,02049	0,994	0,872	1,51E-12
HNRNPA1	6,36E-17	19,12765	0,955	0,553	1,52E-12
BTF3	6,50E-17	16,61651	0,972	0,66	1,55E-12
HLA-E	7,76E-17	11,00346	0,961	0,553	1,85E-12
HLA-DRB1	8,48E-17	172,668	0,989	0,787	2,02E-12
SEM1	8,78E-17	-1,07298	0	0,362	2,09E-12
FGR	8,83E-17	7,758872	0,882	0,362	2,10E-12
RPL11	9,46E-17	55,66835	0,994	1	2,25E-12
CECR1	9,99E-17	2,92373	0,753	0	2,38E-12
PFN1	1,03E-16	34,11252	0,989	0,957	2,46E-12
SAT1	1,09E-16	48,66837	1	1	2,60E-12
CTSS	1,13E-16	17,44048	0,994	0,872	2,68E-12

C7orf73	1,14E-16	1,958313	0,747	0	2,72E-12
MT-ND6	1,15E-16	13,7698	0,781	0,043	2,75E-12
RPS3A	1,16E-16	71,66836	1	0,979	2,77E-12
H3F3A	1,25E-16	26,38268	0,989	0,979	2,98E-12
RPS23	1,39E-16	44,68653	1	1	3,32E-12
RPL22	1,81E-16	32,65442	0,989	0,979	4,31E-12
SRSF5	1,89E-16	10,34021	0,933	0,574	4,51E-12
RPL37A	1,89E-16	51,33304	0,994	0,957	4,51E-12
NLRP3	1,92E-16	6,24446	0,787	0,064	4,57E-12
S100A11	2,01E-16	33,66837	1	0,979	4,79E-12
FTH1	2,39E-16	155,6684	1	1	5,69E-12
MT-CYB	2,63E-16	24,66823	1	0,872	6,25E-12
LCP2	2,76E-16	7,027864	0,809	0,128	6,57E-12
TRAPPC5	2,82E-16	2,719776	0,781	0,064	6,72E-12
RPS3	2,94E-16	57,66483	0,994	0,979	7,01E-12
RPL9	3,10E-16	27,67141	0,994	0,936	7,38E-12
CYBB	3,18E-16	10,39778	0,86	0,298	7,57E-12
CLIC1	3,56E-16	7,016204	0,966	0,489	8,47E-12
NR4A2	3,59E-16	10,66412	0,848	0,191	8,54E-12
APLP2	3,77E-16	6,915264	0,921	0,426	8,98E-12
KLF4	4,72E-16	12,21659	0,865	0,234	1,12E-11
SERPINB1	4,92E-16	12,99467	0,904	0,362	1,17E-11
RPS15A	5,33E-16	50,66836	0,994	0,979	1,27E-11
ARPC2	6,91E-16	9,497306	0,972	0,574	1,65E-11
RBM3	6,99E-16	9,147137	0,933	0,468	1,67E-11
FLNA	7,12E-16	9,180988	0,865	0,255	1,70E-11
SARAF	7,80E-16	9,164282	0,882	0,277	1,86E-11
TRIR	8,24E-16	-1,09582	0	0,34	1,96E-11
ATP5PD	8,28E-16	-1,07167	0	0,34	1,97E-11
ATP5MC3	8,29E-16	-1,5388	0	0,34	1,97E-11
SERPINA1	8,49E-16	15,06936	0,966	0,702	2,02E-11
KLF6	8,68E-16	48,43028	0,921	0,489	2,07E-11
RPL4	9,09E-16	22,78078	0,994	0,745	2,17E-11
RPL8	9,81E-16	36,64875	1	0,957	2,34E-11
C5AR1	1,02E-15	8,707775	0,843	0,277	2,43E-11
RPL13	1,11E-15	72,66836	1	1	2,65E-11
JMJD1C	1,14E-15	6,906828	0,798	0,128	2,72E-11
ATP5B	1,20E-15	8,022742	0,725	0	2,86E-11
PLEK	1,25E-15	5,974016	0,826	0,17	2,98E-11
MT-CO3	1,28E-15	36,79529	1	1	3,05E-11
UBC	1,33E-15	16,94471	0,983	0,66	3,17E-11
H3F3B	1,37E-15	69,78829	0,994	0,915	3,27E-11
CDKN1A	1,44E-15	10,07981	0,798	0,128	3,44E-11
RPL5	1,45E-15	39,66123	0,972	0,83	3,45E-11
LGALS3	1,59E-15	17,47647	0,927	0,383	3,78E-11
ATP5F1	1,63E-15	3,709493	0,719	0	3,89E-11
ARPC3	1,69E-15	15,60676	0,972	0,745	4,02E-11
C11orf31	1,77E-15	2,438954	0,713	0	4,21E-11
VCAN	1,87E-15	46,27834	0,961	0,723	4,45E-11
YBX1	2,21E-15	21,61609	0,955	0,532	5,26E-11

CD37	2,24E-15	6,740944	0,949	0,553	5,33E-11
AHNAK	2,32E-15	14,74298	0,888	0,277	5,54E-11
RPL14	2,41E-15	34,11262	0,994	0,872	5,75E-11
MSN	2,54E-15	5,131687	0,837	0,277	6,05E-11
OAZ1	2,83E-15	21,21926	0,994	0,957	6,74E-11
HLA-DRA	2,90E-15	185,975	0,989	0,851	6,91E-11
SELK	3,00E-15	4,854168	0,708	0	7,15E-11
UQCRB	3,82E-15	8,284301	0,944	0,638	9,09E-11
S100A10	4,07E-15	100,4964	0,989	0,809	9,69E-11
RPS17	4,56E-15	11,43055	0,938	0,596	1,09E-10
JUND	4,90E-15	8,35585	0,854	0,298	1,17E-10
CD52	4,99E-15	13,67098	0,961	0,553	1,19E-10
EIF5	5,11E-15	5,144748	0,781	0,128	1,22E-10
IER3	5,84E-15	19,83673	0,798	0,17	1,39E-10
RPL18A	6,36E-15	66,88186	1	0,979	1,51E-10
EIF4G2	6,77E-15	2,797766	0,888	0,34	1,61E-10
RPL32	7,03E-15	93,66832	0,994	1	1,67E-10
STMP1	7,34E-15	-0,6133	0	0,319	1,75E-10
ATP5F1B	7,34E-15	-0,74984	0	0,319	1,75E-10
RIPOR2	7,38E-15	-0,79572	0	0,319	1,76E-10
AC245014.	7,47E-15	-2,67286	0	0,319	1,78E-10
YWHAZ	8,84E-15	19,78991	0,921	0,426	2,11E-10
CCNL1	9,08E-15	4,637642	0,865	0,362	2,16E-10
RPL34	9,22E-15	60,34878	1	1	2,20E-10
LINC00936	1,01E-14	6,145838	0,697	0	2,42E-10
ACSL1	1,08E-14	6,616202	0,77	0,106	2,57E-10
CD55	1,09E-14	8,733502	0,848	0,234	2,59E-10
GRB2	1,15E-14	5,45904	0,815	0,128	2,75E-10
CRYAB	1,20E-14	-6,19922	0,006	0,34	2,87E-10
FOSL2	1,33E-14	4,832142	0,747	0,064	3,17E-10
CNBP	1,36E-14	4,113413	0,91	0,34	3,23E-10
HSP90AB1	1,53E-14	33,21377	0,854	0,298	3,64E-10
GNG5	1,56E-14	6,207137	0,955	0,574	3,72E-10
RPL36	1,77E-14	39,54865	0,994	1	4,22E-10
UBE2D3	2,01E-14	5,526148	0,893	0,426	4,78E-10
RPL36AL	2,43E-14	14,35245	0,961	0,489	5,78E-10
NCF2	2,50E-14	4,732066	0,831	0,255	5,95E-10
FCN1	2,58E-14	14,7171	0,989	0,872	6,14E-10
HCLS1	2,68E-14	9,318668	0,876	0,277	6,39E-10
MYL12A	2,85E-14	7,996512	0,927	0,489	6,79E-10
REL	2,86E-14	4,469249	0,747	0,106	6,81E-10
TMSB10	2,89E-14	79,90118	0,994	1	6,88E-10
CRIP1	3,05E-14	47,65676	0,82	0,234	7,27E-10
ZFAND5	3,51E-14	13,91802	0,826	0,255	8,37E-10
HNRNPA2E	3,57E-14	9,399248	0,949	0,489	8,50E-10
EEF1B2	3,70E-14	19,02841	0,916	0,532	8,81E-10
ATF3	3,81E-14	28,71415	0,719	0,064	9,07E-10
PFDN5	4,51E-14	11,98362	1	1	1,07E-09
RPS16	5,73E-14	47,09856	0,994	1	1,36E-09
RAC2	5,87E-14	4,658449	0,798	0,17	1,40E-09

RHOG	5,97E-14	4,849344	0,837	0,34	1,42E-09
ATP5D	6,02E-14	3,315384	0,669	0	1,43E-09
ATP5J	6,14E-14	2,917408	0,669	0	1,46E-09
RPL12	6,41E-14	31,93872	0,994	0,979	1,53E-09
RPL36A	6,49E-14	10,18734	0,904	0,553	1,55E-09
ATP5F1A	6,53E-14	-0,59331	0	0,298	1,56E-09
RTRAF	6,56E-14	-0,64676	0	0,298	1,56E-09
ATP5F1C	6,57E-14	-1,15812	0	0,298	1,56E-09
MIOX	6,59E-14	-1,45576	0	0,298	1,57E-09
RBP5	6,62E-14	-2,13755	0	0,298	1,58E-09
ALDOB	6,63E-14	-5,26211	0	0,298	1,58E-09
LRRFIP1	6,70E-14	5,329127	0,865	0,362	1,60E-09
TYROBP	7,04E-14	20,36109	0,994	1	1,68E-09
CST3	7,41E-14	86,66836	0,994	0,979	1,77E-09
GRN	7,49E-14	13,0742	0,955	0,596	1,78E-09
HMGN2	7,69E-14	5,716733	0,815	0,255	1,83E-09
TMA7	8,07E-14	7,852349	0,966	0,766	1,92E-09
UCP2	8,38E-14	14,38776	0,815	0,255	2,00E-09
CD302	8,60E-14	5,34676	0,809	0,17	2,05E-09
SERP1	9,23E-14	7,074422	0,961	0,745	2,20E-09
CSTA	9,47E-14	7,413468	0,916	0,596	2,25E-09
NBPF26	9,75E-14	2,811015	0,657	0	2,32E-09
TKT	1,05E-13	10,25531	0,944	0,702	2,49E-09
PTMA	1,06E-13	23,71742	0,994	0,915	2,53E-09
TPM3	1,20E-13	11,48544	0,933	0,447	2,85E-09
RPS25	1,25E-13	40,70672	0,994	0,979	2,99E-09
THEMIS2	1,28E-13	2,181867	0,702	0,043	3,04E-09
SHFM1	1,37E-13	4,891549	0,657	0	3,26E-09
TMBIM6	1,50E-13	3,347094	0,843	0,34	3,58E-09
C1orf162	1,65E-13	10,58725	0,927	0,447	3,94E-09
SAMSN1	1,80E-13	10,6407	0,798	0,255	4,28E-09
GADD45B	1,85E-13	9,635668	0,753	0,149	4,42E-09
RPL15	1,88E-13	35,66979	0,989	0,936	4,48E-09
PPIF	2,02E-13	7,585442	0,725	0,085	4,82E-09
RPS15	2,11E-13	49,5413	1	1	5,02E-09
RPS27A	2,39E-13	54,50277	1	1	5,69E-09
GLTSCR2	2,43E-13	2,416476	0,646	0	5,79E-09
ARF1	2,59E-13	3,818697	0,843	0,298	6,16E-09
RPS9	2,82E-13	32,53545	1	1	6,72E-09
MYH9	2,85E-13	7,687574	0,747	0,128	6,79E-09
MYL6	3,02E-13	14,32083	0,994	0,894	7,20E-09
CSF3R	3,17E-13	5,032884	0,826	0,277	7,55E-09
TOMM7	3,52E-13	5,239783	0,949	0,745	8,39E-09
TAGLN2	3,66E-13	15,09055	0,888	0,34	8,72E-09
RPS27	3,74E-13	96,60245	1	1	8,90E-09
SRSF7	3,76E-13	6,888867	0,691	0,064	8,95E-09
ZYX	3,91E-13	5,315787	0,781	0,255	9,31E-09
PLIN2	4,30E-13	39,91185	0,708	0,085	1,02E-08
GLUL	4,61E-13	28,91256	0,871	0,404	1,10E-08
GSTP1	4,79E-13	-8,19806	0,961	0,745	1,14E-08

SPI1	4,99E-13	21,05583	0,944	0,532	1,19E-08
GABARAPL	5,13E-13	5,775428	0,685	0,064	1,22E-08
ARF5	5,49E-13	5,617427	0,77	0,191	1,31E-08
SELENOK	5,69E-13	-0,51523	0	0,277	1,35E-08
ATP5IF1	5,69E-13	-0,66483	0	0,277	1,35E-08
NOP53	5,73E-13	-0,62743	0	0,277	1,37E-08
ATP5MC1	5,75E-13	-1,09078	0	0,277	1,37E-08
AC007952.	5,76E-13	-3,26772	0	0,277	1,37E-08
PTPRE	5,78E-13	4,866226	0,843	0,298	1,38E-08
ATP5C1	5,88E-13	3,819118	0,635	0	1,40E-08
DAZAP2	5,95E-13	5,152374	0,921	0,447	1,42E-08
ITM2B	6,24E-13	1,361002	0,966	0,66	1,49E-08
SGK1	6,35E-13	26,43886	0,764	0,213	1,51E-08
HLA-DQB1	7,33E-13	66,65472	0,865	0,404	1,75E-08
ARRB2	8,86E-13	5,265365	0,865	0,34	2,11E-08
TREM1	9,54E-13	8,779719	0,753	0,191	2,27E-08
EVI2B	9,81E-13	4,007495	0,848	0,298	2,34E-08
HNRNPDL	1,05E-12	4,358799	0,787	0,213	2,49E-08
UPP1	1,14E-12	4,32143	0,713	0,128	2,72E-08
ASAH1	1,37E-12	4,704864	0,933	0,489	3,25E-08
AHR	1,38E-12	2,946461	0,674	0,064	3,29E-08
PKM	1,38E-12	9,001107	0,854	0,383	3,29E-08
MYADM	1,38E-12	5,025266	0,685	0,085	3,30E-08
ADGRE5	1,40E-12	5,66484	0,725	0,128	3,34E-08
ZEB2	1,51E-12	10,85342	0,848	0,447	3,60E-08
HMGB1	1,51E-12	4,916575	0,904	0,511	3,60E-08
RPL38	1,59E-12	15,63541	0,983	0,894	3,80E-08
CHCHD2	1,71E-12	7,976787	0,927	0,489	4,07E-08
SERF2	1,71E-12	16,76478	0,994	1	4,08E-08
PER1	1,74E-12	2,007213	0,635	0,021	4,14E-08
HERPUD1	1,78E-12	10,51744	0,775	0,255	4,24E-08
MGAT1	1,82E-12	9,428705	0,781	0,213	4,33E-08
DUSP1	1,90E-12	73,60751	0,989	0,915	4,52E-08
TGFBI	1,98E-12	6,382218	0,669	0,064	4,72E-08
HCST	2,41E-12	6,90804	0,938	0,596	5,74E-08
COTL1	2,44E-12	43,09969	0,978	0,766	5,81E-08
ATP5A1	2,51E-12	5,603631	0,612	0	5,98E-08
SLC25A3	2,57E-12	8,545646	0,854	0,34	6,12E-08
NEAT1	2,60E-12	35,53818	1	0,957	6,20E-08
RPS4Y1	2,79E-12	7,394733	0,893	0,468	6,65E-08
MYEOV2	2,86E-12	2,766606	0,607	0	6,80E-08
CTSB	2,89E-12	30,02646	0,904	0,574	6,88E-08
CD53	2,94E-12	3,502797	0,775	0,213	7,01E-08
TPM4	2,99E-12	5,277529	0,798	0,234	7,13E-08
CXCR4	3,06E-12	15,86781	0,736	0,149	7,30E-08
CORO1A	3,15E-12	10,1786	0,933	0,702	7,51E-08
DDX21	3,30E-12	4,630279	0,798	0,255	7,86E-08
AKAP13	3,39E-12	3,643799	0,713	0,149	8,07E-08
ANXA2	3,84E-12	28,45717	0,933	0,489	9,15E-08
SLC25A6	3,92E-12	20,56587	0,972	0,809	9,35E-08

ARHGDIA	4,12E-12	2,828608	0,747	0,17	9,81E-08
UBA52	4,41E-12	18,99403	1	0,979	1,05E-07
EIF3K	4,83E-12	8,298451	0,899	0,489	1,15E-07
CD48	4,84E-12	6,045322	0,848	0,426	1,15E-07
JPT1	4,89E-12	-0,49315	0	0,255	1,17E-07
ADA2	4,91E-12	-0,55207	0	0,255	1,17E-07
ATP2B1-AS	4,94E-12	-0,79106	0	0,255	1,18E-07
PDZK1IP1	4,94E-12	-9,15007	0	0,255	1,18E-07
ANP32B	5,19E-12	2,710759	0,758	0,191	1,24E-07
ITGB2	5,28E-12	10,64811	0,978	0,702	1,26E-07
CTSH	5,44E-12	3,572737	0,781	0,213	1,30E-07
HNRNPK	5,51E-12	4,193263	0,882	0,383	1,31E-07
RPS18	5,97E-12	90,97519	0,994	1	1,42E-07
HLA-DPA1	6,50E-12	69,64994	0,933	0,532	1,55E-07
C14orf166	6,56E-12	3,053109	0,596	0	1,56E-07
HIF1A	6,81E-12	10,01125	0,73	0,191	1,62E-07
ATP6V1G1	6,84E-12	4,173436	0,86	0,447	1,63E-07
HSPA8	6,85E-12	18,42034	0,742	0,213	1,63E-07
ODF3B	6,87E-12	5,70104	0,742	0,17	1,64E-07
TCEB1	7,24E-12	1,604755	0,59	0	1,72E-07
BHLHE40	7,60E-12	8,596864	0,685	0,106	1,81E-07
LILRB2	7,97E-12	3,2478	0,781	0,277	1,90E-07
S100A4	8,13E-12	47,35006	0,994	1	1,94E-07
TALDO1	8,22E-12	4,172714	0,921	0,447	1,96E-07
SLC2A3	8,58E-12	40,13514	0,843	0,447	2,04E-07
YWHAB	8,58E-12	6,919919	0,882	0,447	2,04E-07
RPL22L1	8,74E-12	4,910457	0,657	0,064	2,08E-07
RPS14	9,07E-12	45,66836	1	1	2,16E-07
HLA-DQA1	9,18E-12	54,33244	0,691	0,149	2,19E-07
IQGAP1	1,04E-11	4,796582	0,792	0,298	2,48E-07
CSDE1	1,07E-11	2,855748	0,787	0,234	2,56E-07
ATP6V0B	1,10E-11	3,084175	0,899	0,489	2,62E-07
HBEGF	1,15E-11	3,355741	0,68	0,106	2,73E-07
CYTIP	1,22E-11	6,888374	0,64	0,064	2,91E-07
NPM1	1,33E-11	6,659375	0,843	0,34	3,18E-07
RIPK2	1,36E-11	5,215344	0,624	0,043	3,25E-07
RPS4X	1,38E-11	22,66907	0,989	0,936	3,28E-07
PGK1	1,42E-11	8,03499	0,831	0,426	3,37E-07
SON	1,46E-11	2,710431	0,775	0,298	3,49E-07
JUNB	1,47E-11	15,68176	0,961	0,766	3,51E-07
FPR1	1,52E-11	2,485423	0,882	0,447	3,63E-07
KDM6B	1,58E-11	3,363597	0,713	0,149	3,75E-07
RPS6	1,62E-11	66,66752	0,989	0,957	3,86E-07
MAFB	1,64E-11	8,613776	0,82	0,34	3,91E-07
PDE4B	1,67E-11	5,724966	0,697	0,149	3,97E-07
RPL10	1,68E-11	67,98163	1	1	4,00E-07
HIGD2A	1,73E-11	5,704229	0,899	0,532	4,12E-07
B2M	1,74E-11	104,6683	1	1	4,16E-07
FTL	1,76E-11	213,6684	1	1	4,20E-07
EIF3F	1,80E-11	11,85549	0,831	0,383	4,29E-07

MS4A6A	1,98E-11	30,42869	0,91	0,596	4,72E-07
DNAJB1	2,33E-11	24,74369	0,64	0,064	5,55E-07
MYL12B	2,47E-11	5,755035	0,871	0,489	5,87E-07
ZNF331	2,76E-11	16,98279	0,663	0,085	6,56E-07
RASGEF1B	2,88E-11	4,898669	0,624	0,043	6,85E-07
STK17B	2,88E-11	5,38862	0,798	0,255	6,86E-07
RPS19	3,25E-11	44,97837	1	0,979	7,75E-07
PTPRC	3,27E-11	12,07041	1	1	7,79E-07
MT1E	3,43E-11	-3,11414	0,034	0,362	8,17E-07
VAPA	3,47E-11	4,246946	0,758	0,277	8,26E-07
TNFRSF1B	3,85E-11	5,601333	0,809	0,362	9,18E-07
RAB20	4,07E-11	2,367901	0,607	0,043	9,69E-07
SELENOW	4,13E-11	-0,57192	0	0,234	9,83E-07
SELENOT	4,16E-11	-0,5876	0	0,234	9,91E-07
MMP24OS	4,17E-11	-0,64135	0	0,234	9,92E-07
PPIB	4,23E-11	12,38823	0,815	0,298	1,01E-06
SLC25A5	4,31E-11	8,960231	0,831	0,404	1,03E-06
TLN1	4,35E-11	2,639711	0,68	0,128	1,04E-06
CAPZB	4,40E-11	3,307956	0,798	0,255	1,05E-06
SUMO2	4,50E-11	6,12085	0,91	0,574	1,07E-06
VASP	4,57E-11	2,315832	0,691	0,149	1,09E-06
COMMD6	4,58E-11	5,299715	0,921	0,66	1,09E-06
GPX4	4,67E-11	7,640438	0,938	0,723	1,11E-06
BTG2	4,71E-11	4,374924	0,691	0,149	1,12E-06
SELT	4,97E-11	2,166001	0,562	0	1,18E-06
SSH2	5,05E-11	2,658682	0,64	0,085	1,20E-06
HLA-DPB1	5,90E-11	60,64762	0,904	0,553	1,40E-06
SFPQ	6,79E-11	2,333132	0,764	0,213	1,62E-06
ATPIF1	6,95E-11	1,574232	0,556	0	1,66E-06
ZFAS1	7,68E-11	4,372846	0,876	0,553	1,83E-06
MIF	7,89E-11	10,16234	0,831	0,404	1,88E-06
RPSA	8,43E-11	35,60658	0,938	0,745	2,01E-06
EIF4B	8,81E-11	2,525166	0,73	0,213	2,10E-06
GLIPR1	9,95E-11	2,657431	0,826	0,319	2,37E-06
ATP6AP2	9,97E-11	2,602086	0,674	0,128	2,37E-06
NFKBIZ	1,05E-10	23,95259	0,758	0,255	2,50E-06
PLSCR1	1,05E-10	3,097612	0,747	0,255	2,51E-06
RPL27	1,07E-10	10,72304	0,989	0,936	2,54E-06
IVNS1ABP	1,08E-10	2,99796	0,646	0,085	2,58E-06
C8orf59	1,09E-10	2,535043	0,685	0,128	2,60E-06
ARL4A	1,10E-10	6,95123	0,697	0,213	2,61E-06
ITGAX	1,20E-10	7,57709	0,685	0,17	2,87E-06
PCBP2	1,25E-10	4,405568	0,876	0,532	2,97E-06
MT-ND2	1,27E-10	-4,66932	1	0,894	3,03E-06
LIMD2	1,31E-10	5,533324	0,753	0,234	3,11E-06
C20orf24	1,36E-10	1,692649	0,545	0	3,23E-06
EIF3L	1,53E-10	6,89405	0,826	0,34	3,65E-06
EIF3A	1,54E-10	3,472898	0,663	0,149	3,67E-06
DPYD	1,63E-10	2,416198	0,702	0,191	3,88E-06
NFE2L2	1,73E-10	2,44365	0,713	0,191	4,12E-06

IFNGR1	1,81E-10	3,216557	0,775	0,213	4,31E-06
TBXAS1	2,02E-10	3,860887	0,685	0,17	4,80E-06
LRP1	2,03E-10	2,73518	0,669	0,128	4,84E-06
CTSD	2,08E-10	4,095135	0,882	0,447	4,95E-06
RPS5	2,28E-10	19,80641	0,966	0,872	5,44E-06
MPEG1	2,31E-10	5,400641	0,831	0,383	5,51E-06
EIF3G	2,41E-10	3,850618	0,792	0,298	5,74E-06
GPSM3	2,47E-10	2,029525	0,843	0,468	5,87E-06
HNRNPF	2,50E-10	2,346195	0,725	0,191	5,96E-06
USP15	2,61E-10	2,964438	0,708	0,213	6,21E-06
GNAI2	2,66E-10	4,286876	0,843	0,447	6,33E-06
TSC22D3	2,67E-10	22,18302	0,758	0,298	6,36E-06
BNIP3L	2,79E-10	3,659498	0,73	0,213	6,64E-06
THBS1	2,84E-10	28,24582	0,629	0,128	6,78E-06
NR4A3	2,97E-10	7,176085	0,556	0,021	7,09E-06
LDHA	3,04E-10	17,7977	0,775	0,277	7,24E-06
IRF2BP2	3,08E-10	2,907045	0,68	0,191	7,33E-06
ELF1	3,10E-10	6,566062	0,669	0,149	7,38E-06
RPS11	3,37E-10	11,16995	0,994	0,915	8,03E-06
CCNI	3,39E-10	5,827771	0,91	0,681	8,08E-06
CYTOR	3,44E-10	-0,38183	0	0,213	8,20E-06
PCK1	3,47E-10	-1,27479	0	0,213	8,26E-06
LINC00493	3,52E-10	1,447384	0,528	0	8,38E-06
SAMHD1	3,63E-10	9,084227	0,747	0,319	8,66E-06
KLF10	3,82E-10	3,320855	0,624	0,106	9,10E-06
GPR183	3,87E-10	30,16404	0,68	0,149	9,21E-06
ICAM1	3,98E-10	4,126023	0,551	0,021	9,47E-06
FKBP1A	4,57E-10	4,453652	0,893	0,553	1,09E-05
HNRNPA3	4,89E-10	3,770431	0,792	0,298	1,17E-05
15. Sep	5,01E-10	1,98508	0,522	0	1,19E-05
CD300E	5,25E-10	11,08773	0,798	0,404	1,25E-05
RP11-1143	5,31E-10	5,849417	0,522	0	1,26E-05
QKI	5,61E-10	1,461751	0,556	0,021	1,34E-05
STX11	5,83E-10	2,51042	0,635	0,128	1,39E-05
GATM	5,91E-10	-2,6458	0,017	0,277	1,41E-05
OSM	6,02E-10	9,893755	0,522	0	1,43E-05
RPL23A	6,02E-10	21,11823	0,989	0,936	1,43E-05
FAM65B	6,45E-10	3,029617	0,517	0	1,54E-05
CRTAP	6,92E-10	1,995221	0,697	0,213	1,65E-05
RPL3	7,08E-10	33,66832	0,989	0,957	1,69E-05
FGL2	7,47E-10	5,912323	0,826	0,511	1,78E-05
PET100	7,48E-10	2,107145	0,747	0,298	1,78E-05
PRRC2C	7,55E-10	2,622184	0,702	0,213	1,80E-05
TPI1	7,60E-10	6,640297	0,826	0,404	1,81E-05
C19orf60	7,63E-10	1,416368	0,511	0	1,82E-05
EIF3H	7,83E-10	3,166644	0,837	0,468	1,87E-05
HNRNPC	8,00E-10	4,158047	0,82	0,489	1,90E-05
CD99	8,35E-10	4,940116	0,831	0,383	1,99E-05
COX4I1	8,63E-10	11,75308	0,972	0,894	2,05E-05
PLXDC2	8,65E-10	3,923876	0,629	0,128	2,06E-05

G0S2	8,79E-10	50,00379	0,685	0,213	2,09E-05
TNFAIP2	9,76E-10	1,664878	0,612	0,085	2,32E-05
PILRA	1,06E-09	2,115202	0,674	0,17	2,52E-05
TIMP1	1,10E-09	74,66836	0,938	0,596	2,61E-05
FCER1G	1,11E-09	23,31867	0,994	1	2,65E-05
ACTR3	1,15E-09	3,464677	0,736	0,319	2,74E-05
PSMA6	1,16E-09	3,383568	0,573	0,064	2,76E-05
OST4	1,16E-09	2,888686	0,916	0,66	2,77E-05
GNAS	1,17E-09	3,12439	0,663	0,17	2,80E-05
BCLAF1	1,18E-09	2,229242	0,573	0,064	2,81E-05
DUSP2	1,19E-09	8,006459	0,601	0,106	2,84E-05
DNAJA1	1,28E-09	4,529224	0,702	0,234	3,04E-05
CXCL8	1,28E-09	39,78784	0,635	0,149	3,05E-05
EIF4H	1,35E-09	1,719411	0,528	0,021	3,23E-05
NAPA	1,47E-09	2,133808	0,539	0,021	3,50E-05
SUB1	1,50E-09	4,632781	0,921	0,681	3,57E-05
RILPL2	1,53E-09	4,436919	0,719	0,255	3,64E-05
RAB31	1,63E-09	2,213526	0,719	0,191	3,89E-05
PRR13	1,66E-09	3,195981	0,826	0,489	3,94E-05
HSP90B1	1,70E-09	6,299528	0,702	0,17	4,06E-05
HLA-DMA	1,74E-09	10,42065	0,876	0,489	4,15E-05
SEPW1	1,81E-09	2,874504	0,5	0	4,32E-05
YPEL5	1,85E-09	3,172683	0,635	0,149	4,42E-05
CDC37	1,88E-09	3,737239	0,685	0,213	4,47E-05
HSPA5	2,00E-09	6,894525	0,68	0,191	4,77E-05
RPLP2	2,00E-09	35,56396	0,994	1	4,77E-05
TUBA1A	2,01E-09	29,76497	0,697	0,213	4,80E-05
CSTB	2,03E-09	12,43237	0,831	0,447	4,85E-05
UBE2J1	2,11E-09	2,319057	0,562	0,064	5,03E-05
CELF2	2,18E-09	4,466123	0,713	0,255	5,19E-05
ENO1	2,26E-09	9,632075	0,871	0,489	5,37E-05
EMD	2,29E-09	1,548329	0,601	0,106	5,45E-05
RAB24	2,33E-09	1,468188	0,517	0,021	5,55E-05
APBB1IP	2,53E-09	3,034506	0,539	0,043	6,03E-05
RANBP2	2,59E-09	4,858838	0,556	0,064	6,17E-05
SDCBP	2,71E-09	4,771472	0,871	0,553	6,45E-05
PSME1	2,76E-09	7,870171	0,865	0,447	6,57E-05
EEF1D	2,79E-09	7,061767	0,972	0,894	6,64E-05
RTF2	2,82E-09	-0,28445	0	0,191	6,72E-05
SQOR	2,83E-09	-0,35656	0	0,191	6,74E-05
NDUFAF8	2,83E-09	-0,35656	0	0,191	6,74E-05
REX1BD	2,83E-09	-0,52977	0	0,191	6,74E-05
AL034397.	2,84E-09	-0,42382	0	0,191	6,76E-05
RAB5IF	2,84E-09	-0,48684	0	0,191	6,77E-05
ELOC	2,84E-09	-0,64043	0	0,191	6,77E-05
LAPTM4A	2,89E-09	3,001872	0,685	0,17	6,88E-05
TMED10	2,89E-09	1,599113	0,635	0,149	6,88E-05
UBL5	2,92E-09	2,990993	0,938	0,681	6,96E-05
KDELR1	3,08E-09	1,180886	0,596	0,085	7,34E-05
FCGR2A	3,10E-09	2,065308	0,747	0,298	7,38E-05

ATP1B3	3,27E-09	4,959877	0,697	0,277	7,78E-05
XRCC6	3,30E-09	1,892336	0,556	0,064	7,86E-05
CHD1	3,47E-09	3,067229	0,646	0,17	8,26E-05
H2AFY	3,47E-09	3,270136	0,809	0,426	8,27E-05
TAGAP	3,66E-09	3,343685	0,573	0,085	8,71E-05
FAM26F	3,68E-09	4,018023	0,489	0	8,76E-05
ZNF385A	3,84E-09	2,835175	0,607	0,106	9,14E-05
CFP	4,07E-09	6,607641	0,843	0,468	9,69E-05
NAIP	4,43E-09	2,935677	0,652	0,149	0,000106
POMP	4,43E-09	4,372479	0,843	0,489	0,000106
GDI2	4,50E-09	4,143719	0,742	0,34	0,000107
GNG10	4,55E-09	1,715464	0,483	0	0,000108
ETS2	4,71E-09	3,990254	0,596	0,106	0,000112
SH3BGRL	4,81E-09	6,000714	0,837	0,596	0,000114
SPP1	4,81E-09	-19,1307	0,011	0,234	0,000115
ADRBK1	4,82E-09	1,400769	0,478	0	0,000115
FKBP5	4,97E-09	7,150375	0,646	0,17	0,000118
SNHG15	5,01E-09	3,235323	0,506	0,021	0,000119
TMEM258	5,07E-09	2,93238	0,871	0,574	0,000121
AIF1	5,16E-09	19,38915	0,983	0,936	0,000123
HLA-F	5,22E-09	1,5447	0,674	0,191	0,000124
FCGRT	5,26E-09	2,08948	0,899	0,511	0,000125
PSMB1	5,30E-09	2,497795	0,758	0,319	0,000126
IL10RA	5,30E-09	2,120666	0,618	0,149	0,000126
EIF4A1	5,31E-09	5,658056	0,775	0,319	0,000127
UBXN11	5,34E-09	1,446607	0,607	0,128	0,000127
EWSR1	5,42E-09	1,396907	0,567	0,085	0,000129
MXD1	5,44E-09	3,622559	0,646	0,17	0,00013
RPL10A	5,62E-09	10,82415	0,983	0,957	0,000134
PLBD1	5,65E-09	4,174264	0,567	0,085	0,000135
CAP1	5,75E-09	8,876289	0,837	0,532	0,000137
WDR83OS	5,89E-09	2,346647	0,831	0,447	0,00014
EREG	5,95E-09	11,29286	0,59	0,128	0,000142
SQSTM1	5,99E-09	2,733409	0,663	0,191	0,000143
SF3B1	6,10E-09	2,46904	0,674	0,213	0,000145
WASF2	6,42E-09	2,526848	0,596	0,106	0,000153
STRAP	6,54E-09	2,014834	0,545	0,064	0,000156
NUMB	6,55E-09	1,784663	0,652	0,191	0,000156
PNP	6,61E-09	4,598918	0,478	0	0,000157
POLD4	6,77E-09	3,160202	0,522	0,043	0,000161
MBNL1	6,83E-09	3,653447	0,764	0,319	0,000163
SOCS3	6,87E-09	3,25046	0,68	0,213	0,000164
SLC43A2	6,89E-09	2,300508	0,596	0,106	0,000164
PGD	7,01E-09	1,913607	0,697	0,255	0,000167
ROCK1	7,05E-09	2,474101	0,624	0,149	0,000168
SYAP1	7,55E-09	4,785723	0,573	0,085	0,00018
HNRNPU	7,68E-09	5,112105	0,787	0,404	0,000183
NDUFA13	7,79E-09	2,626458	0,725	0,298	0,000186
FERMT3	7,91E-09	1,5339	0,579	0,106	0,000188
XRN2	7,94E-09	1,852553	0,618	0,149	0,000189

NDUFA6	8,06E-09	2,411506	0,64	0,191	0,000192
PDIA3	8,16E-09	9,597808	0,607	0,149	0,000194
EMP3	8,25E-09	6,503521	0,91	0,681	0,000196
RPL35	8,27E-09	29,64895	0,989	0,979	0,000197
EIF3D	9,04E-09	1,408602	0,629	0,17	0,000215
CTNNB1	9,06E-09	4,167608	0,719	0,277	0,000216
ATF4	9,10E-09	1,38456	0,579	0,106	0,000217
OLR1	9,32E-09	7,844777	0,472	0	0,000222
CD36	9,34E-09	6,542642	0,725	0,255	0,000223
TRA2B	9,36E-09	3,745018	0,691	0,277	0,000223
MYD88	9,52E-09	2,27171	0,556	0,085	0,000227
AP1S2	9,69E-09	14,04386	0,854	0,617	0,000231
PCBP1	1,01E-08	5,158177	0,91	0,574	0,000242
COX5A	1,03E-08	3,024702	0,747	0,319	0,000246
SEC61B	1,04E-08	5,689827	0,854	0,447	0,000247
ATP6V1F	1,07E-08	2,095907	0,888	0,532	0,000255
SLA	1,07E-08	2,671853	0,708	0,277	0,000255
CYTH1	1,23E-08	1,133186	0,511	0,043	0,000294
COX6A1	1,24E-08	4,045257	0,916	0,723	0,000295
MAT2A	1,27E-08	4,538935	0,713	0,277	0,000303
RGCC	1,28E-08	17,69128	0,494	0,021	0,000306
ATP5G1	1,29E-08	1,603758	0,461	0	0,000307
CPPED1	1,32E-08	2,281074	0,601	0,128	0,000315
RGS10	1,33E-08	2,465746	0,691	0,234	0,000317
AREG	1,38E-08	71,44763	0,562	0,085	0,000329
CTSC	1,47E-08	4,986316	0,657	0,191	0,000349
TOMM20	1,47E-08	1,869037	0,691	0,234	0,00035
CSRNP1	1,51E-08	2,082979	0,551	0,085	0,00036
PPT1	1,52E-08	5,897432	0,685	0,255	0,000363
CLEC4E	1,58E-08	7,771701	0,612	0,149	0,000376
BRK1	1,70E-08	3,641897	0,865	0,553	0,000405
CPVL	1,73E-08	10,04676	0,831	0,532	0,000413
RRP12	1,78E-08	2,492971	0,455	0	0,000423
STAT3	1,82E-08	2,643333	0,545	0,085	0,000433
POLR1D	1,86E-08	2,233291	0,663	0,234	0,000444
CD93	1,89E-08	3,609109	0,646	0,213	0,00045
ENSA	1,90E-08	1,149099	0,539	0,064	0,000453
NINJ1	1,91E-08	6,266031	0,669	0,213	0,000456
BACH1	1,95E-08	4,836108	0,669	0,255	0,000466
MYO1G	1,97E-08	2,400844	0,596	0,128	0,000469
DDIT4	1,97E-08	9,874419	0,522	0,064	0,00047
LTA4H	2,04E-08	4,920638	0,702	0,277	0,000486
MFSD1	2,04E-08	3,371926	0,663	0,234	0,000486
GRINA	2,10E-08	2,521624	0,674	0,213	0,000499
LITAF	2,17E-08	16,75696	0,719	0,34	0,000517
C1orf43	2,19E-08	1,396206	0,596	0,128	0,000523
MRPS24	2,29E-08	1,296929	0,449	0	0,000545
HGD	2,29E-08	-0,25656	0	0,17	0,000546
GRK2	2,29E-08	-0,25656	0	0,17	0,000546
SELENOF	2,30E-08	-0,33064	0	0,17	0,000547

AC004687.	2,30E-08	-0,33064	0	0,17	0,000547
AC005280.	2,30E-08	-0,3996	0	0,17	0,000548
RIDA	2,30E-08	-0,46412	0	0,17	0,000548
RAN	2,34E-08	3,448927	0,702	0,277	0,000557
HNRNPUL1	2,34E-08	1,198029	0,522	0,064	0,000557
CLNS1A	2,41E-08	1,318754	0,522	0,064	0,000574
EIF1B	2,43E-08	3,408589	0,685	0,234	0,000578
ID2	2,44E-08	13,55564	0,697	0,234	0,000581
YWHAE	2,57E-08	2,317987	0,669	0,234	0,000613
RTN3	2,84E-08	1,283761	0,562	0,106	0,000675
TRIB1	2,87E-08	2,291505	0,472	0,021	0,000683
ERP29	2,88E-08	5,125127	0,775	0,362	0,000687
CANX	2,90E-08	4,238054	0,635	0,191	0,000692
FAM96A	2,96E-08	2,051982	0,539	0,085	0,000706
PGLS	3,00E-08	4,248683	0,736	0,34	0,000714
WAS	3,02E-08	1,718976	0,674	0,213	0,000719
CHURC1	3,04E-08	1,823037	0,573	0,128	0,000725
LGALS1	3,05E-08	23,30867	0,983	1	0,000726
PGAM1	3,13E-08	2,999254	0,719	0,298	0,000745
CCDC109B	3,26E-08	1,446746	0,444	0	0,000778
UBE2I	3,29E-08	1,461828	0,567	0,106	0,000784
CSNK1A1	3,39E-08	1,610845	0,657	0,191	0,000808
MINOS1	3,44E-08	2,595391	0,787	0,404	0,00082
AKIRIN2	3,55E-08	4,51929	0,596	0,149	0,000845
ACTR2	3,55E-08	9,877881	0,798	0,511	0,000846
ACADVL	3,57E-08	1,172846	0,551	0,085	0,000852
BZW1	3,63E-08	1,549738	0,584	0,128	0,000864
CD14	3,69E-08	11,38518	0,921	0,745	0,000879
APRT	3,70E-08	3,174502	0,792	0,426	0,000882
TRAPPC1	4,00E-08	3,087003	0,764	0,319	0,000952
RNF213	4,00E-08	1,600238	0,573	0,128	0,000953
HCK	4,05E-08	3,680544	0,713	0,319	0,000964
CXCL16	4,09E-08	3,819334	0,652	0,213	0,000973
SRSF3	4,11E-08	3,903986	0,82	0,511	0,000979
SEC11A	4,12E-08	3,259787	0,809	0,383	0,000981
RNF130	4,19E-08	4,640831	0,82	0,383	0,000997
MT-ND4	4,19E-08	9,657136	1	0,936	0,000999
EIF2S3	4,38E-08	2,507753	0,607	0,17	0,001042
OXA1L	4,38E-08	2,034555	0,551	0,106	0,001044
FAM49B	4,59E-08	3,118621	0,685	0,255	0,001094
PTEN	4,64E-08	1,327636	0,522	0,064	0,001105
09. Sep	4,64E-08	2,266575	0,584	0,128	0,001106
HLA-DMB	5,38E-08	2,340325	0,68	0,213	0,001281
PELI1	5,44E-08	4,132297	0,461	0,021	0,001295
CALM1	5,69E-08	4,843837	0,82	0,532	0,001356
CHMP1B	5,96E-08	10,3229	0,635	0,213	0,001419
RP11-670E	5,96E-08	3,31796	0,433	0	0,00142
C19orf70	6,35E-08	-0,65494	0,567	0,106	0,001512
SRRM1	6,38E-08	2,34978	0,708	0,298	0,00152
IL10RB	6,43E-08	0,9128	0,427	0	0,001531

RTFDC1	6,78E-08	1,131547	0,427	0	0,001616
ZFP36L2	6,80E-08	18,67207	0,725	0,383	0,00162
CFD	6,87E-08	4,731722	0,876	0,638	0,001637
ROMO1	6,89E-08	1,634735	0,669	0,255	0,00164
KRTCAP2	6,92E-08	2,066792	0,691	0,213	0,001648
DNAJB6	7,11E-08	3,398362	0,663	0,234	0,001693
PLP2	7,14E-08	3,027938	0,713	0,34	0,0017
RSL1D1	7,36E-08	2,617537	0,646	0,213	0,001754
ATP2B1	7,51E-08	8,643152	0,657	0,255	0,00179
EDF1	7,57E-08	0,605668	0,854	0,574	0,001803
USP3	7,85E-08	1,271188	0,5	0,064	0,001871
THBD	7,90E-08	7,325213	0,562	0,106	0,001881
EID1	8,21E-08	4,721455	0,697	0,277	0,001955
NDUFA12	8,38E-08	3,141989	0,607	0,17	0,001997
ALOX5	8,43E-08	1,092019	0,5	0,064	0,002007
PNPLA2	8,58E-08	0,937255	0,421	0	0,002043
LILRB3	9,40E-08	1,869192	0,59	0,106	0,00224
CNN2	9,46E-08	2,36899	0,567	0,128	0,002253
LYN	1,01E-07	1,564306	0,663	0,191	0,002403
ARF4	1,04E-07	2,304973	0,534	0,106	0,002469
SRA1	1,04E-07	1,709913	0,607	0,191	0,002477
PECAM1	1,04E-07	1,682098	0,596	0,17	0,002486
PTGER2	1,04E-07	1,737783	0,472	0,043	0,002487
CFLAR	1,06E-07	3,812364	0,607	0,191	0,002524
SRRM2	1,08E-07	1,42699	0,674	0,255	0,002562
PNPLA8	1,08E-07	1,906538	0,494	0,064	0,002581
PAK2	1,15E-07	1,887571	0,64	0,213	0,002737
LGALS9	1,17E-07	2,647739	0,652	0,234	0,002786
EIF3I	1,23E-07	1,250171	0,567	0,128	0,002929
TCF25	1,24E-07	1,332229	0,629	0,213	0,002945
INSIG1	1,26E-07	11,95461	0,489	0,064	0,002992
PNISR	1,27E-07	1,712383	0,612	0,17	0,003014
SMAP2	1,29E-07	2,802312	0,758	0,362	0,003066
CTB-61M7	1,31E-07	1,375743	0,416	0	0,003125
RTN4	1,40E-07	1,839969	0,691	0,277	0,003342
ACAP2	1,40E-07	1,499452	0,601	0,191	0,003344
SYNGR2	1,41E-07	2,804347	0,635	0,213	0,00336
SF1	1,43E-07	1,909974	0,612	0,213	0,003403
IER2	1,44E-07	13,09193	0,848	0,638	0,003437
CXCL2	1,49E-07	41,54939	0,489	0,064	0,003551
C16orf13	1,53E-07	0,994197	0,41	0	0,003656
CARD16	1,58E-07	4,939081	0,843	0,553	0,003771
SERBP1	1,59E-07	1,92807	0,607	0,149	0,003792
RAB7A	1,61E-07	3,541807	0,669	0,277	0,003846
CARD19	1,64E-07	1,838321	0,438	0,021	0,003898
HMHA1	1,64E-07	1,207198	0,41	0	0,003905
UTRN	1,69E-07	1,272608	0,551	0,128	0,004025
RBPJ	1,69E-07	2,428002	0,601	0,191	0,00403
IDS	1,71E-07	1,548488	0,551	0,128	0,004073
NSA2	1,73E-07	2,855813	0,601	0,191	0,004113

PHACTR1	1,75E-07	7,177347	0,669	0,34	0,004157
S100A9	1,75E-07	138,6708	0,989	1	0,004166
REEP5	1,75E-07	1,577977	0,64	0,234	0,004177
RNF149	1,77E-07	2,540051	0,798	0,489	0,004207
OTUB1	1,77E-07	1,261118	0,567	0,149	0,00422
IRAK3	1,78E-07	1,546649	0,539	0,106	0,004235
ANAPC11	1,81E-07	1,578662	0,635	0,213	0,004314
COX7C	1,83E-07	6,706942	0,955	0,851	0,004352
CENPX	1,84E-07	-0,30402	0	0,149	0,004385
NAT8	1,84E-07	-8,1503	0	0,149	0,004391
FABP1	1,84E-07	-0,44086	0	0,149	0,004393
MTRNR2L6	1,85E-07	-0,65418	0	0,149	0,004395
GSTA2	1,85E-07	-2,33698	0	0,149	0,004396
CCNH	1,96E-07	6,51484	0,545	0,106	0,004676
NONO	1,98E-07	1,464364	0,534	0,106	0,004724
ADGRE2	2,02E-07	1,73832	0,522	0,106	0,004817
PPDPF	2,04E-07	5,393587	0,798	0,511	0,004851
WNK1	2,06E-07	1,077075	0,483	0,064	0,004901
PYCARD	2,07E-07	8,170925	0,854	0,553	0,004923
SKP1	2,23E-07	3,865012	0,792	0,511	0,005308
KMT2E	2,23E-07	4,532154	0,567	0,128	0,005311
VIMP	2,25E-07	1,430418	0,404	0	0,005363
TUBA1B	2,30E-07	14,2499	0,685	0,34	0,005471
NCKAP1L	2,30E-07	1,292829	0,522	0,106	0,005485
SLC3A2	2,30E-07	1,93306	0,534	0,106	0,005485
DDX17	2,32E-07	1,470743	0,635	0,213	0,005526
HM13	2,48E-07	1,556483	0,567	0,128	0,005901
TUBB4B	2,49E-07	1,743639	0,478	0,064	0,005934
LILRB1	2,56E-07	4,674035	0,562	0,149	0,006089
SH3BP2	2,58E-07	1,718463	0,478	0,064	0,006147
TWF2	2,58E-07	1,16643	0,607	0,17	0,00615
APH1A	2,63E-07	0,953181	0,466	0,043	0,006256
LST1	2,68E-07	17,80281	0,966	0,894	0,006382
LILRA2	2,73E-07	2,130891	0,5	0,085	0,006511
UQCRH	2,74E-07	3,318762	0,916	0,745	0,006528
UBE2L6	2,76E-07	2,661576	0,5	0,085	0,006578
ETV6	2,76E-07	0,989779	0,399	0	0,006584
PTBP3	2,77E-07	1,080274	0,478	0,064	0,00659
NEDD9	2,88E-07	2,490245	0,427	0,021	0,00685
NDUFB4	3,08E-07	1,66316	0,646	0,255	0,007338
PTPN6	3,15E-07	2,408028	0,607	0,191	0,007495
SSR2	3,17E-07	5,623956	0,753	0,383	0,007553
HNRNPA0	3,17E-07	2,457568	0,596	0,191	0,007562
SPCS3	3,20E-07	1,403846	0,556	0,149	0,007622
UQCRC1	3,29E-07	1,461995	0,618	0,213	0,007832
PSMB10	3,30E-07	2,297096	0,702	0,319	0,007856
WDR1	3,31E-07	1,929103	0,596	0,191	0,007885
EPC1	3,31E-07	1,761187	0,517	0,106	0,007893
MFF	3,34E-07	1,073355	0,449	0,043	0,007952
PRKCB	3,52E-07	1,306537	0,59	0,17	0,008385

HN1	3,61E-07	1,030886	0,393	0	0,008605
RUNX1	3,68E-07	1,146108	0,421	0,021	0,008765
AUP1	3,76E-07	1,473996	0,472	0,064	0,008953
CLEC2B	3,78E-07	3,160003	0,579	0,17	0,00901
COX7B	3,89E-07	3,73499	0,815	0,532	0,009278
CALR	3,92E-07	17,19182	0,618	0,234	0,009343
COX16	3,99E-07	0,915085	0,494	0,085	0,009513
NFIL3	4,18E-07	2,131367	0,466	0,064	0,009966
TOP1	4,23E-07	2,30469	0,607	0,213	0,010076
PFKFB3	4,24E-07	3,851192	0,545	0,149	0,010107
TMSB4X	4,26E-07	66,66836	1	1	0,010155
DBI	4,40E-07	1,475319	0,86	0,532	0,010485
SKIL	4,41E-07	1,208633	0,444	0,043	0,01051
UBE2V1	4,42E-07	1,073032	0,444	0,043	0,010528
ZCCHC6	4,56E-07	1,067832	0,388	0	0,010861
SSR4	4,73E-07	2,883277	0,826	0,468	0,011265
XBP1	4,76E-07	1,986256	0,601	0,191	0,011348
JAK1	4,80E-07	1,539383	0,567	0,17	0,011438
LIMS1	4,82E-07	6,474298	0,584	0,191	0,011481
KLF2	4,91E-07	14,00236	0,601	0,213	0,01169
TMED5	4,92E-07	1,414695	0,489	0,085	0,011724
ATP6AP1	4,95E-07	1,359333	0,511	0,106	0,011785
UBE2D2	4,99E-07	1,742652	0,624	0,213	0,011894
NCL	5,01E-07	3,594856	0,674	0,277	0,011926
WSB1	5,01E-07	4,193408	0,674	0,298	0,011943
EIF4A3	5,06E-07	5,618608	0,478	0,064	0,012046
RPN1	5,10E-07	1,28129	0,562	0,149	0,012158
CTD-3252(	5,23E-07	5,171966	0,388	0	0,012461
FBXL5	5,39E-07	2,358765	0,618	0,213	0,012832
NUDT16	5,62E-07	3,387332	0,483	0,085	0,013381
BANF1	5,62E-07	2,111343	0,607	0,191	0,013385
CD163	5,72E-07	22,16638	0,68	0,319	0,013614
MKNK2	5,72E-07	1,011369	0,382	0	0,013626
PLK3	5,74E-07	1,235726	0,438	0,043	0,013675
PPP1R18	5,82E-07	2,250069	0,573	0,17	0,013852
RNPS1	5,91E-07	1,481413	0,528	0,128	0,014084
RBM8A	5,98E-07	1,843954	0,562	0,17	0,014249
LYST	5,99E-07	2,917129	0,612	0,234	0,014267
RPN2	6,13E-07	1,23134	0,5	0,085	0,014604
DNTTIP2	6,23E-07	1,224601	0,41	0,021	0,014848
EIF5A	6,25E-07	2,75325	0,522	0,128	0,014881
CAST	6,27E-07	2,840102	0,708	0,34	0,014944
CAPN2	6,50E-07	1,408595	0,461	0,064	0,015473
PLEKHB2	6,59E-07	1,025938	0,461	0,064	0,015703
TOM1	6,65E-07	1,228397	0,461	0,064	0,015835
GSTK1	6,94E-07	2,965937	0,725	0,319	0,016519
NAP1L1	7,16E-07	4,789182	0,764	0,489	0,017051
POLR2E	7,17E-07	2,103079	0,567	0,149	0,017083
PTGES3	7,23E-07	3,071314	0,646	0,255	0,017232
KIAA1033	7,45E-07	0,855235	0,376	0	0,017748

SQRDL	7,45E-07	0,855235	0,376	0	0,017748
TAPBP	7,49E-07	1,919628	0,64	0,277	0,017837
SNRPD3	7,50E-07	1,325368	0,556	0,17	0,017865
DSE	7,51E-07	3,77358	0,506	0,106	0,017894
IFITM2	7,54E-07	24,42965	0,888	0,702	0,017954
LINC00657	7,55E-07	1,213229	0,376	0	0,017986
AFF4	7,74E-07	0,975391	0,433	0,043	0,018441
CASP4	7,84E-07	1,147281	0,691	0,298	0,018677
CD164	8,06E-07	3,375668	0,618	0,213	0,019197
TNFSF13	8,08E-07	1,26561	0,376	0	0,019239
C9orf142	8,14E-07	1,023164	0,376	0	0,019384
PREX1	8,24E-07	1,238738	0,404	0,021	0,019639
LINC00152	8,54E-07	8,825493	0,376	0	0,020341
AP2M1	8,61E-07	1,110448	0,59	0,191	0,0205
FAM49A	8,63E-07	1,730284	0,556	0,17	0,020566
ARPC5	8,94E-07	3,035252	0,848	0,617	0,021291
IFNGR2	8,99E-07	2,254617	0,612	0,234	0,021423
ARHGAP4	9,01E-07	1,414598	0,478	0,085	0,021458
ETF1	9,01E-07	1,318791	0,551	0,17	0,021467
BLOC1S6	9,05E-07	1,113037	0,478	0,085	0,021556
XRCC5	9,07E-07	1,90805	0,534	0,149	0,021609
ALDH2	9,08E-07	3,621259	0,669	0,255	0,021637
C19orf38	9,09E-07	2,816936	0,618	0,255	0,021651
KYNU	9,14E-07	1,114822	0,511	0,106	0,021762
FOXO3	9,22E-07	1,871609	0,478	0,085	0,021953
UBR4	9,25E-07	0,988299	0,478	0,085	0,022038
ENY2	9,68E-07	1,773548	0,764	0,426	0,023063
UXT	9,73E-07	2,114446	0,674	0,319	0,02317
DDX3X	1,00E-06	3,715507	0,685	0,34	0,023865
UBXN4	1,01E-06	2,338527	0,545	0,149	0,024052
LMAN2	1,01E-06	1,214339	0,438	0,043	0,02411
RNH1	1,01E-06	2,921637	0,725	0,319	0,024174
RNF7	1,02E-06	1,661442	0,607	0,255	0,024307
APEX1	1,03E-06	1,946216	0,449	0,064	0,024628
TRAM1	1,04E-06	1,238285	0,472	0,085	0,024655
MAN2B1	1,04E-06	1,256605	0,399	0,021	0,024737
RAB5C	1,05E-06	1,75116	0,652	0,277	0,024911
SERTAD1	1,05E-06	1,616957	0,449	0,064	0,024968
CIRBP	1,08E-06	4,764003	0,753	0,383	0,025833
UBXN1	1,14E-06	2,406378	0,691	0,362	0,02711
MDH2	1,15E-06	1,05308	0,539	0,128	0,027391
RBM4	1,17E-06	0,789103	0,365	0	0,027962
FUS	1,21E-06	1,496089	0,68	0,255	0,028908
SERINC1	1,22E-06	1,422795	0,472	0,085	0,028995
IRS2	1,25E-06	3,865162	0,517	0,128	0,029809
CD4	1,26E-06	1,394265	0,573	0,191	0,030095
CCDC69	1,27E-06	0,931111	0,483	0,085	0,030353
PPP1R2	1,30E-06	1,460336	0,494	0,106	0,030871
FLII	1,32E-06	0,909286	0,421	0,043	0,031525
BIRC3	1,32E-06	4,352619	0,365	0	0,031551

UQCRC2	1,34E-06	1,062542	0,528	0,128	0,031823
STAT6	1,34E-06	0,903721	0,421	0,043	0,031914
GNA13	1,36E-06	1,619793	0,489	0,106	0,032285
HK3	1,36E-06	0,716365	0,393	0,021	0,03231
EGR1	1,37E-06	8,819699	0,629	0,277	0,03264
RBKS	1,41E-06	2,340094	0,393	0,021	0,033653
LDLR	1,43E-06	1,326857	0,444	0,064	0,033976
RGS19	1,45E-06	1,290667	0,551	0,149	0,034464
PHLDA1	1,45E-06	8,901962	0,416	0,043	0,03456
RHOB	1,46E-06	24,41472	0,73	0,426	0,034832
MCUB	1,46E-06	-0,27667	0	0,128	0,034843
CMBL	1,46E-06	-0,27667	0	0,128	0,034843
POLR2J3.1	1,46E-06	-0,27667	0	0,128	0,034843
HPD	1,46E-06	-0,48049	0	0,128	0,034868
MTRNR2L1	1,46E-06	-1,02679	0	0,128	0,034888
MS4A7	1,54E-06	5,235015	0,697	0,362	0,036655
GUK1	1,55E-06	2,352176	0,775	0,511	0,036854
LAP3	1,55E-06	1,846022	0,489	0,106	0,03702
UBQLN1	1,58E-06	0,685706	0,36	0	0,03759
C17orf62	1,58E-06	1,45429	0,36	0	0,037654
MMP24-AS	1,58E-06	1,158553	0,36	0	0,0377
CLTA	1,60E-06	2,094879	0,657	0,277	0,038059
BLVRB	1,63E-06	2,444503	0,775	0,426	0,038778
NCOA4	1,65E-06	1,591242	0,579	0,213	0,03919
SYK	1,65E-06	1,235267	0,545	0,17	0,039284
SNRPD2	1,65E-06	3,106407	0,809	0,489	0,039308
TGIF1	1,65E-06	1,232527	0,438	0,064	0,039348
FMNL1	1,70E-06	1,344108	0,618	0,213	0,040473
OXSR1	1,70E-06	1,06928	0,36	0	0,040482
PSMB4	1,71E-06	0,977597	0,489	0,106	0,040721
ZC3H15	1,72E-06	1,272888	0,534	0,128	0,040997
EIF3M	1,74E-06	2,255303	0,646	0,298	0,04134
BCL3	1,74E-06	1,173635	0,416	0,043	0,041543
DDOST	1,75E-06	1,258385	0,416	0,043	0,041667
VPS37B	1,76E-06	1,572103	0,36	0	0,041903
ANP32A	1,78E-06	0,983574	0,489	0,106	0,042339
SHKBP1	1,79E-06	0,972296	0,539	0,149	0,042594
ETHE1	1,81E-06	1,602015	0,388	0,021	0,043001
EHD1	1,81E-06	1,835721	0,438	0,064	0,043094
PSMA7	1,81E-06	4,939612	0,871	0,638	0,043222
SLC38A2	1,81E-06	1,119961	0,388	0,021	0,043231
SNRPG	1,82E-06	1,401685	0,736	0,404	0,043265
PID1	1,82E-06	3,766745	0,511	0,128	0,043393
DDX3Y	1,84E-06	1,443468	0,472	0,085	0,043798
APOBEC3A	1,87E-06	16,74888	0,596	0,213	0,044485
MAPRE1	1,87E-06	0,876376	0,478	0,085	0,044651
TAF1D	1,90E-06	2,113995	0,506	0,128	0,045211
CCL3L3	1,92E-06	8,984621	0,36	0	0,045821
MAGOH	1,93E-06	1,199391	0,539	0,149	0,045907
TMEM219	1,94E-06	1,186985	0,719	0,362	0,046276

RASSF5	1,98E-06	1,268766	0,483	0,106	0,047179
SNX10	1,99E-06	3,258811	0,59	0,191	0,047509
ERH	2,12E-06	1,230201	0,618	0,255	0,050478
WARS	2,13E-06	4,739841	0,624	0,277	0,050704
AP2S1	2,16E-06	2,3254	0,888	0,638	0,051562
DUSP6	2,17E-06	11,76791	0,736	0,426	0,051594
SH3KBP1	2,22E-06	1,494223	0,579	0,213	0,052914
DBNL	2,25E-06	0,975209	0,494	0,106	0,053705
SPTLC2	2,26E-06	1,411997	0,444	0,064	0,053737
SRSF10	2,29E-06	1,641262	0,461	0,085	0,054554
CXCL14	2,32E-06	-8,14494	0,006	0,149	0,055207
TCIRG1	2,33E-06	1,456978	0,539	0,149	0,055583
FCAR	2,34E-06	2,26541	0,382	0,021	0,055854
TRMT1	2,36E-06	0,877109	0,494	0,106	0,056239
GSTA1	2,36E-06	-2,35314	0,006	0,149	0,056264
KCTD12	2,37E-06	2,654726	0,567	0,191	0,056412
AGTRAP	2,37E-06	2,391182	0,663	0,319	0,05651
SMIM24	2,41E-06	-1,33406	0,006	0,149	0,057326
ABRACL	2,43E-06	1,579717	0,624	0,298	0,057997
SNRPB	2,44E-06	1,618783	0,567	0,17	0,058113
PLEC	2,48E-06	2,256259	0,433	0,064	0,059126
SIVA1	2,49E-06	0,757253	0,461	0,085	0,059327
RGS2	2,60E-06	2,475378	0,82	0,574	0,061858
CASP1	2,63E-06	1,895928	0,713	0,362	0,062536
CALCOCO2	2,63E-06	1,320923	0,455	0,085	0,062643
02. Sep	2,63E-06	1,240177	0,455	0,085	0,062659
OSTF1	2,64E-06	1,80079	0,607	0,213	0,062908
HMGB2	2,68E-06	3,695363	0,624	0,298	0,063795
CNPY3	2,71E-06	2,380868	0,764	0,404	0,064565
HNRNPL	2,72E-06	0,966215	0,433	0,064	0,064693
PDCD6	2,72E-06	1,089492	0,5	0,128	0,064731
PSMB3	2,72E-06	1,555528	0,787	0,404	0,064767
GGNBP2	2,77E-06	0,913391	0,433	0,064	0,066057
EZR	2,81E-06	3,815428	0,545	0,191	0,06696
CMTM7	2,87E-06	0,76271	0,551	0,149	0,068327
RBM17	2,93E-06	0,864387	0,466	0,085	0,069734
LAT2	2,94E-06	1,121375	0,478	0,106	0,069956
SCPEP1	2,95E-06	1,47084	0,522	0,149	0,070372
TMEM59	2,96E-06	1,44632	0,68	0,277	0,070603
PNN	2,97E-06	0,953068	0,376	0,021	0,07066
TSPAN14	2,99E-06	1,279729	0,376	0,021	0,071254
VDAC1	3,01E-06	1,493013	0,517	0,106	0,071626
GMFG	3,09E-06	4,168073	0,927	0,723	0,073493
COX8A	3,10E-06	6,205811	0,86	0,638	0,073847
COMT	3,12E-06	1,582088	0,579	0,213	0,074342
DAD1	3,22E-06	1,956445	0,646	0,319	0,07676
RNMT	3,26E-06	1,043125	0,455	0,085	0,077765
ADAM17	3,27E-06	1,049352	0,478	0,106	0,077985
S100A8	3,28E-06	167,6684	0,983	1	0,078071
ITGB1	3,33E-06	1,54622	0,539	0,17	0,07941

SORL1	3,35E-06	1,4754	0,449	0,085	0,079811
YWHAQ	3,37E-06	1,511533	0,472	0,106	0,080367
WHSC1L1	3,54E-06	0,844855	0,343	0	0,084429
SPCS1	3,61E-06	1,317095	0,685	0,319	0,086109
SSR1	3,73E-06	1,673489	0,539	0,149	0,088737
HSPD1	3,73E-06	20,64113	0,506	0,149	0,088814
GNPTG	3,86E-06	0,877309	0,371	0,021	0,091965
MTPN	3,91E-06	1,20244	0,685	0,383	0,09307
GNS	3,94E-06	2,516788	0,5	0,128	0,093969
NIPBL	4,05E-06	0,831874	0,449	0,085	0,09642
VDAC2	4,06E-06	2,104311	0,624	0,255	0,096757
PABPC4	4,15E-06	2,370959	0,517	0,149	0,098953
TGFB1	4,19E-06	1,510693	0,584	0,213	0,099737
CLK1	4,22E-06	1,114001	0,539	0,17	0,100511
CCL3	4,25E-06	39,42315	0,444	0,085	0,101206
BID	4,38E-06	1,776148	0,573	0,213	0,104245
RABAC1	4,46E-06	1,317267	0,607	0,213	0,106207
DMXL2	4,46E-06	2,328105	0,539	0,191	0,106302
NBPF14	4,48E-06	1,547619	0,444	0,085	0,106756
TAX1BP1	4,49E-06	2,746533	0,612	0,234	0,106936
CAPG	4,52E-06	19,43409	0,674	0,34	0,107655
ATP6V0D1	4,54E-06	1,552964	0,691	0,383	0,108071
RNASET2	4,58E-06	3,512838	0,781	0,468	0,109214
CMTM6	4,59E-06	4,008291	0,725	0,426	0,109359
MIDN	4,66E-06	2,107987	0,579	0,255	0,110932
PRAM1	4,82E-06	1,090751	0,466	0,106	0,114786
ZFP36L1	4,84E-06	16,3177	0,86	0,681	0,115172
DDIT3	4,99E-06	1,680257	0,365	0,021	0,118795
TRABD	5,05E-06	0,836564	0,393	0,043	0,120358
NDUFV2	5,06E-06	1,572677	0,522	0,128	0,120436
CSNK2B	5,14E-06	1,641956	0,674	0,298	0,12243
LAIR1	5,26E-06	3,720471	0,427	0,064	0,125262
HSBP1	5,30E-06	3,718604	0,781	0,404	0,12617
CAPNS1	5,30E-06	1,478334	0,629	0,298	0,126224
ERBB2IP	5,49E-06	0,661092	0,331	0	0,130777
GNB2	5,50E-06	2,081235	0,663	0,34	0,130897
UQCR11	5,66E-06	4,907217	0,933	0,809	0,13483
EIF3E	5,73E-06	8,983527	0,826	0,617	0,136588
MYO1F	5,87E-06	1,454913	0,669	0,362	0,139807
TOMM5	5,90E-06	1,123482	0,449	0,085	0,140649
ELOVL5	5,91E-06	1,067346	0,416	0,064	0,14078
DCTN3	5,99E-06	1,19222	0,461	0,106	0,142648
ATP13A3	6,07E-06	2,093294	0,522	0,17	0,144593
C9orf72	6,15E-06	1,645542	0,489	0,128	0,146451
NOTCH2	6,24E-06	0,336432	0,455	0,085	0,148747
LILRA1	6,33E-06	0,915051	0,36	0,021	0,150725
HSPE1	6,34E-06	18,95035	0,64	0,277	0,151137
SRI	6,36E-06	1,144862	0,449	0,085	0,151409
GSTO1	6,37E-06	5,315166	0,764	0,447	0,151616
DDX6	6,41E-06	0,874801	0,388	0,043	0,152587

P4HB	6,42E-06	1,561963	0,573	0,213	0,152834
THUMPD3-	6,44E-06	0,606509	0,427	0,064	0,153302
SF3B6	6,49E-06	1,627724	0,573	0,234	0,154701
TUBGCP2	6,52E-06	0,651982	0,388	0,043	0,15529
TMEM256	6,61E-06	1,137878	0,618	0,277	0,157507
FO538757.	6,66E-06	0,624537	0,326	0	0,15869
CYCS	6,68E-06	7,025282	0,64	0,34	0,159068
NABP1	6,72E-06	1,25599	0,461	0,106	0,160139
H2AFJ	6,75E-06	1,602067	0,5	0,128	0,160731
HMGA1	6,78E-06	1,659693	0,433	0,085	0,161557
DIAPH1	6,89E-06	1,377814	0,455	0,106	0,164005
LINC-PINT	6,95E-06	0,925399	0,326	0	0,165567
ADAM10	6,99E-06	1,427781	0,461	0,106	0,166452
GTF3A	6,99E-06	3,746774	0,494	0,149	0,16662
MRFAP1	7,28E-06	0,903493	0,461	0,106	0,173314
RARA	7,57E-06	0,933914	0,461	0,106	0,180249
TUBA1C	7,59E-06	1,973467	0,365	0,021	0,180882
CSF2RA	7,60E-06	1,3297	0,382	0,043	0,181127
SNHG8	7,65E-06	2,49085	0,573	0,234	0,182284
RAB1B	7,65E-06	1,08628	0,41	0,064	0,182327
TBCB	7,66E-06	0,799434	0,472	0,106	0,182379
ITGAM	7,75E-06	1,197201	0,461	0,085	0,18452
SYNCRIP	7,76E-06	1,04105	0,41	0,064	0,184781
VPS13C	7,77E-06	1,0198	0,5	0,149	0,185089
DOK3	7,83E-06	0,943726	0,41	0,064	0,186452
BASP1	7,83E-06	1,722626	0,382	0,043	0,186596
KTN1	7,89E-06	3,603587	0,534	0,17	0,187857
BIRC6	8,03E-06	0,735712	0,354	0,021	0,191198
PARVG	8,04E-06	1,433132	0,567	0,213	0,191522
SMG1	8,09E-06	0,739124	0,354	0,021	0,19264
SREK1	8,10E-06	0,809146	0,354	0,021	0,19285
TMPO	8,12E-06	1,152128	0,354	0,021	0,193514
MIR22HG	8,14E-06	1,278726	0,354	0,021	0,193864
RASSF4	8,15E-06	2,894362	0,382	0,043	0,194231
AMD1	8,22E-06	0,8138	0,382	0,043	0,19573
ARHGAP3C	8,29E-06	1,361104	0,466	0,106	0,197544
AGFG1	8,60E-06	0,884094	0,433	0,085	0,204883
TNFRSF14	8,60E-06	0,856648	0,483	0,128	0,204953
GK	8,62E-06	7,572801	0,522	0,191	0,205268
SF3A1	8,82E-06	0,801063	0,32	0	0,210138
VPS35	9,05E-06	1,351044	0,489	0,128	0,215672
CD63	9,15E-06	-0,69439	0,865	0,638	0,217973
ATG3	9,69E-06	2,031841	0,567	0,213	0,230831
FBL	9,71E-06	1,437573	0,438	0,085	0,231315
ARL4C	9,90E-06	4,035179	0,421	0,085	0,235768
SSFA2	1,01E-05	0,609097	0,348	0,021	0,240517
UFM1	1,01E-05	1,33153	0,449	0,106	0,241129
GHITM	1,01E-05	1,996663	0,573	0,234	0,241506
CTBS	1,03E-05	0,653286	0,348	0,021	0,245262
USP36	1,04E-05	1,136737	0,348	0,021	0,247521

RNF13	1,04E-05	2,108074	0,545	0,191	0,247647
RPS2	1,05E-05	26,66835	0,989	0,979	0,249828
GABARAPL	1,06E-05	1,328281	0,635	0,298	0,251999
SSB	1,07E-05	1,411409	0,478	0,128	0,253939
CSNK1D	1,08E-05	0,784667	0,376	0,043	0,257382
AMPD2	1,08E-05	1,155973	0,376	0,043	0,257636
STK4	1,08E-05	3,712526	0,478	0,128	0,258094
GUSB	1,09E-05	0,715379	0,376	0,043	0,260272
RBM47	1,10E-05	1,271283	0,472	0,128	0,262567
DOK2	1,10E-05	1,845488	0,612	0,277	0,26303
C11orf73	1,11E-05	0,618136	0,315	0	0,26392
KMT2C	1,11E-05	0,770148	0,511	0,149	0,26553
RP11-108M	1,12E-05	0,669385	0,315	0	0,266268
PRPF8	1,12E-05	0,669385	0,315	0	0,266268
PPP1R15B	1,12E-05	0,926501	0,438	0,085	0,266553
SLC20A1	1,13E-05	1,019405	0,315	0	0,268525
PPP1CA	1,13E-05	2,16638	0,64	0,319	0,269067
SRPRA	1,14E-05	0,764618	0,315	0	0,270681
CUX1	1,14E-05	1,241231	0,472	0,128	0,270948
AZIN1	1,14E-05	1,143605	0,399	0,064	0,271836
WAC	1,15E-05	1,094419	0,494	0,149	0,274255
GLYATL1	1,16E-05	-0,43975	0	0,106	0,275212
S100A1	1,16E-05	-0,5018	0	0,106	0,275346
MTRNR2L1	1,16E-05	-7,16919	0	0,106	0,275436
ARL8B	1,17E-05	0,89634	0,427	0,085	0,277556
RAP1B	1,21E-05	1,831387	0,629	0,319	0,287798
PTP4A2	1,21E-05	2,273393	0,725	0,362	0,288133
MAPK1IP1	1,21E-05	1,014614	0,399	0,064	0,28858
TFRC	1,21E-05	4,008112	0,315	0	0,28885
JUN	1,23E-05	33,25304	0,506	0,17	0,292668
TNFRSF1A	1,24E-05	1,011723	0,567	0,213	0,296073
PSMA1	1,25E-05	2,116672	0,635	0,298	0,297051
FLOT1	1,25E-05	1,44342	0,511	0,17	0,298149
SEC61G	1,25E-05	1,130443	0,758	0,489	0,298209
CSGALNAC	1,25E-05	0,957421	0,399	0,064	0,298334
07. Sep	1,26E-05	1,49755	0,551	0,213	0,301222
AOAH	1,28E-05	1,53272	0,573	0,213	0,305845
CCT6A	1,29E-05	1,332815	0,506	0,17	0,307285
CCT3	1,29E-05	0,604019	0,343	0,021	0,307584
KIF5B	1,29E-05	0,968375	0,511	0,17	0,308147
BTAF1	1,30E-05	0,779073	0,343	0,021	0,309052
FAM204A	1,31E-05	0,751378	0,461	0,106	0,311476
OVCA2	1,31E-05	0,508101	0,309	0	0,311938
PTP4A1	1,31E-05	2,104334	0,494	0,149	0,311972
CCRL2	1,32E-05	1,614065	0,343	0,021	0,314305
WDR45B	1,32E-05	1,440212	0,343	0,021	0,31499
STX10	1,36E-05	0,629637	0,461	0,106	0,324738
SH2B3	1,37E-05	1,155309	0,371	0,043	0,325527
TMX4	1,37E-05	4,818076	0,371	0,043	0,326124
ANXA11	1,37E-05	1,551191	0,596	0,255	0,326124

PTAFR	1,38E-05	0,750369	0,41	0,064	0,327765
ZNF706	1,38E-05	2,341291	0,657	0,362	0,327781
BIN2	1,38E-05	2,025316	0,455	0,106	0,328684
M6PR	1,38E-05	1,609339	0,461	0,128	0,329262
LENG8	1,40E-05	1,196032	0,444	0,106	0,333109
PHB2	1,40E-05	2,805462	0,494	0,149	0,333391
VCP	1,41E-05	1,851524	0,444	0,106	0,335552
HNRNPD	1,43E-05	0,824272	0,545	0,213	0,340517
RNASE6	1,48E-05	3,039956	0,596	0,277	0,351697
PTPN2	1,48E-05	1,394053	0,466	0,106	0,353642
SH3BP5	1,50E-05	2,809414	0,517	0,17	0,356249
POLE4	1,50E-05	0,737787	0,522	0,17	0,356808
PSMB8	1,53E-05	2,556086	0,584	0,277	0,363606
LSM7	1,56E-05	1,717663	0,618	0,255	0,370502
CDV3	1,56E-05	0,826364	0,444	0,106	0,370595
MANBA	1,56E-05	1,048745	0,444	0,106	0,372465
SEC13	1,59E-05	0,920071	0,393	0,064	0,378073
CCDC88A	1,62E-05	2,296898	0,506	0,17	0,385126
PPP4C	1,63E-05	1,121761	0,567	0,213	0,388449
FGFR1OP2	1,63E-05	0,570705	0,337	0,021	0,388841
ATXN2L	1,63E-05	0,570705	0,337	0,021	0,388841
MAFF	1,66E-05	1,39354	0,337	0,021	0,39546
PAFAH1B1	1,67E-05	0,88165	0,393	0,064	0,39687
ARIH2	1,67E-05	0,69182	0,337	0,021	0,398157
TIPARP	1,68E-05	1,82795	0,337	0,021	0,399144
FAM127A	1,70E-05	0,613896	0,303	0	0,403834
UBB	1,70E-05	5,886094	0,775	0,511	0,404212
ZNF207	1,71E-05	1,654887	0,584	0,255	0,407585
APOE	1,72E-05	-11,9986	0,067	0,277	0,410506
VPS28	1,74E-05	1,1908	0,691	0,362	0,413651
DNM2	1,74E-05	0,691979	0,303	0	0,415442
RIOK3	1,75E-05	0,856764	0,365	0,043	0,418025
SUPT6H	1,79E-05	0,742094	0,365	0,043	0,42634
PHF20L1	1,80E-05	0,616276	0,365	0,043	0,428058
SERINC3	1,80E-05	1,054422	0,466	0,128	0,428469
TNFSF13B	1,80E-05	4,568213	0,725	0,447	0,429254
CDC42EP3	1,81E-05	3,940623	0,517	0,191	0,431263
SURF4	1,81E-05	0,75559	0,303	0	0,431295
LINC00116	1,81E-05	0,75559	0,303	0	0,431295
BLVRA	1,81E-05	0,96982	0,556	0,191	0,432045
ERGIC3	1,83E-05	0,78248	0,551	0,191	0,435553
RP5-1171I1	1,85E-05	0,865623	0,303	0	0,441465
KHK	1,86E-05	-0,33972	0,006	0,128	0,444182
BNIP2	1,86E-05	1,132368	0,545	0,191	0,444201
S100A12	1,87E-05	43,6367	0,843	0,702	0,445754
SAP18	1,88E-05	1,61973	0,713	0,362	0,447109
JAML	1,88E-05	3,071141	0,652	0,362	0,447265
SRP72	1,90E-05	1,09754	0,449	0,106	0,453504
HIST2H2A/	1,92E-05	14,81824	0,303	0	0,458213
CCT4	1,93E-05	1,024842	0,438	0,106	0,460265

MOB1A	1,93E-05	1,9925	0,612	0,298	0,460588
METTL7A	1,94E-05	0,789544	0,416	0,085	0,461035
CCL4	1,96E-05	59,82069	0,303	0	0,466181
ELL2	2,06E-05	1,778579	0,36	0,043	0,489893
LY86	2,06E-05	1,717403	0,685	0,426	0,491259
IL1RN	2,06E-05	4,728232	0,343	0,021	0,491687
SLC25A37	2,07E-05	3,38987	0,562	0,234	0,494011
NUTF2	2,09E-05	1,014056	0,388	0,064	0,496939
POLR2A	2,11E-05	0,813129	0,331	0,021	0,502445
PDCD4	2,12E-05	0,777622	0,331	0,021	0,50528
ATP6V1B2	2,14E-05	1,846401	0,607	0,319	0,510046
COPE	2,15E-05	1,642195	0,713	0,383	0,513272
SRSF2	2,19E-05	1,807596	0,567	0,234	0,521394
MORF4L1	2,20E-05	1,561105	0,725	0,404	0,524568
NCOR1	2,21E-05	0,96634	0,478	0,149	0,527302
TMEM14C	2,22E-05	0,560551	0,461	0,106	0,528584
GNB1	2,23E-05	0,894297	0,511	0,17	0,530693
RHOQ	2,24E-05	0,908044	0,36	0,043	0,532769
SLC7A7	2,24E-05	3,200521	0,618	0,34	0,534487
ZNF281	2,25E-05	0,654446	0,298	0	0,535168
SCO2	2,25E-05	0,876835	0,298	0	0,536101
DDX39B	2,26E-05	0,823693	0,36	0,043	0,537299
PCSK1N	2,26E-05	-2,15686	0,011	0,149	0,538969
ATP1A1	2,28E-05	1,541601	0,517	0,17	0,541933
TPP1	2,28E-05	1,498787	0,657	0,319	0,541963
PSMD4	2,28E-05	0,672126	0,36	0,043	0,543906
PFDN2	2,36E-05	0,925421	0,5	0,17	0,561637
MPC1	2,38E-05	1,043752	0,433	0,106	0,566235
SNX2	2,38E-05	0,993216	0,489	0,149	0,566282
OS9	2,41E-05	0,657468	0,534	0,191	0,575093
HSPH1	2,45E-05	5,89035	0,354	0,043	0,582547
MAP3K2	2,49E-05	2,047495	0,489	0,17	0,593992
C4orf3	2,53E-05	1,606946	0,837	0,638	0,603453
GTF3C6	2,59E-05	0,782923	0,466	0,128	0,616028
TMEM176I	2,59E-05	1,106578	0,646	0,319	0,616396
SPAG9	2,59E-05	1,517626	0,382	0,064	0,617465
PHLDA2	2,60E-05	1,510838	0,326	0,021	0,618296
C9orf16	2,62E-05	1,30036	0,579	0,234	0,623577
RELB	2,66E-05	1,129614	0,382	0,064	0,632498
PHC2	2,66E-05	1,010896	0,382	0,064	0,633789
NAAA	2,66E-05	2,416691	0,5	0,17	0,634428
RB1CC1	2,68E-05	2,119571	0,326	0,021	0,63868
SFT2D1	2,68E-05	1,098423	0,579	0,255	0,63872
NFKB2	2,68E-05	0,669575	0,326	0,021	0,63876
DEK	2,71E-05	2,378748	0,607	0,277	0,64621
FES	2,74E-05	0,663716	0,382	0,064	0,653089
SLC15A3	2,75E-05	0,727815	0,382	0,064	0,654633
HEXB	2,76E-05	0,622912	0,528	0,191	0,656409
FNBP4	2,79E-05	0,787771	0,292	0	0,663914
LINC01420	2,79E-05	0,880653	0,292	0	0,664183

SPEN	2,81E-05	0,767049	0,292	0	0,668356
NDUFB9	2,82E-05	1,402917	0,579	0,255	0,67131
IDI1	2,83E-05	1,208191	0,354	0,043	0,67305
CYB5R4	2,87E-05	0,681057	0,382	0,064	0,682937
TM9SF2	2,87E-05	2,056405	0,472	0,149	0,683435
IPO7	2,87E-05	0,758842	0,292	0	0,684683
NFAM1	2,89E-05	0,771053	0,292	0	0,687996
STX12	2,90E-05	0,980685	0,382	0,064	0,689667
ZC3HAV1	2,90E-05	1,810611	0,292	0	0,689956
CREM	2,92E-05	10,68223	0,399	0,085	0,696695
ANO6	2,94E-05	0,616045	0,354	0,043	0,701036
EIF4EBP1	2,97E-05	1,063742	0,427	0,106	0,706771
SHOC2	2,98E-05	1,072272	0,449	0,128	0,710312
MAP2K3	2,98E-05	1,072272	0,449	0,128	0,710312
MRPL33	2,99E-05	2,036199	0,59	0,255	0,712204
SEC31A	3,01E-05	0,781765	0,427	0,106	0,717257
TRIM22	3,07E-05	1,277972	0,399	0,085	0,731751
VPS29	3,09E-05	1,516192	0,59	0,255	0,735147
DPP7	3,10E-05	0,851882	0,427	0,106	0,738765
SCAF11	3,12E-05	1,283668	0,607	0,255	0,74273
LILRA5	3,15E-05	2,942093	0,624	0,319	0,751506
STK10	3,16E-05	1,072781	0,472	0,149	0,752405
IRF1	3,23E-05	4,893284	0,607	0,277	0,76824
PPP1CB	3,23E-05	1,307714	0,663	0,298	0,769389
RSRP1	3,23E-05	1,150249	0,506	0,17	0,770429
BRD2	3,24E-05	1,235783	0,404	0,085	0,771795
SETX	3,26E-05	0,972492	0,427	0,106	0,776293
NR1H2	3,30E-05	1,024842	0,376	0,064	0,784936
TMEM176/	3,34E-05	1,969245	0,506	0,149	0,795161
CLEC4A	3,36E-05	1,179122	0,5	0,191	0,799447
ARHGAP2€	3,37E-05	1,06106	0,36	0,043	0,803562
NUP98	3,38E-05	0,611305	0,32	0,021	0,804835
ATRX	3,40E-05	0,932106	0,427	0,106	0,810785
LYPLA1	3,41E-05	1,074161	0,348	0,043	0,812209
ITPRIP	3,42E-05	0,572993	0,287	0	0,814103
DNAJB11	3,42E-05	0,83465	0,287	0	0,814359
SDHB	3,46E-05	0,770556	0,433	0,106	0,824366
MLF2	3,46E-05	0,800785	0,472	0,149	0,824947
NDEL1	3,47E-05	0,823445	0,376	0,064	0,825814
CHD2	3,47E-05	1,548589	0,348	0,043	0,826802
ARID4B	3,47E-05	1,034295	0,444	0,106	0,827403
C18orf32	3,49E-05	0,858611	0,348	0,043	0,832036
IL13RA1	3,50E-05	1,130811	0,348	0,043	0,832894
SRSF6	3,51E-05	0,723967	0,388	0,064	0,836802
TANK	3,52E-05	0,642298	0,404	0,085	0,839476
NDUFA10	3,53E-05	0,664427	0,438	0,106	0,841943
ATP5O	3,54E-05	0,815989	0,287	0	0,844269
PLD3	3,59E-05	1,620223	0,483	0,149	0,856324
LYAR	3,60E-05	0,720188	0,287	0	0,856624
MED28	3,62E-05	0,775257	0,348	0,043	0,861922

MEF2D	3,62E-05	1,081143	0,287	0	0,8629
CNIH4	3,63E-05	0,929485	0,376	0,064	0,863561
HLA-DQA2	3,64E-05	9,902814	0,41	0,106	0,866993
CDA	3,64E-05	1,701513	0,433	0,106	0,867843
PAIP2	3,65E-05	0,80085	0,652	0,298	0,869614
NRROS	3,68E-05	0,77208	0,348	0,043	0,876378
PPP2CA	3,71E-05	0,921448	0,483	0,17	0,883665
ACSL3	3,73E-05	1,307307	0,354	0,043	0,887645
DAP3	3,73E-05	0,869819	0,438	0,106	0,889207
RBM5	3,73E-05	0,588108	0,348	0,043	0,88926
RP2	3,74E-05	0,574435	0,348	0,043	0,891815
CHMP2A	3,74E-05	1,354634	0,579	0,255	0,891941
ADIPOR1	3,78E-05	1,423444	0,421	0,106	0,900044
DHRS7	3,80E-05	1,508068	0,466	0,149	0,905327
VAMP8	3,81E-05	5,671131	0,876	0,66	0,907981
ERICH1	3,81E-05	0,991349	0,433	0,106	0,908227
DICER1	3,87E-05	0,65275	0,449	0,128	0,922964
LPXN	3,92E-05	0,96449	0,399	0,085	0,934616
PSMB6	3,94E-05	1,326764	0,584	0,277	0,93791
DNAJC8	3,96E-05	0,891799	0,421	0,106	0,942476
TCEA1	3,97E-05	1,342603	0,506	0,191	0,944718
METRNL	3,98E-05	1,050642	0,472	0,149	0,948285
GNAQ	4,04E-05	0,975897	0,444	0,128	0,962708
NDUFB11	4,12E-05	2,188183	0,764	0,489	0,980514
IRAK1	4,18E-05	0,478632	0,281	0	0,995304
DDX46	4,22E-05	1,17347	0,483	0,17	1
SRGAP2	4,23E-05	1,316973	0,315	0,021	1
C16orf72	4,26E-05	1,258208	0,41	0,085	1
THAP9-AS1	4,26E-05	0,643082	0,315	0,021	1
TMEM173	4,30E-05	0,720858	0,354	0,043	1
CTSA	4,41E-05	0,858318	0,506	0,17	1
ANKRD28	4,43E-05	3,019728	0,343	0,043	1
LRRC59	4,43E-05	0,676227	0,281	0	1
SELL	4,44E-05	1,381467	0,517	0,213	1
CHORDC1	4,45E-05	0,689483	0,281	0	1
MTSS1	4,46E-05	1,381264	0,449	0,128	1
HLX	4,48E-05	0,834751	0,281	0	1
CTA-29F11	4,48E-05	1,494667	0,281	0	1
CALM2	4,52E-05	6,74303	0,916	0,83	1
BCL6	4,55E-05	2,07386	0,343	0,043	1
ATP6V0E1	4,55E-05	2,073286	0,837	0,617	1
CSK	4,61E-05	1,51414	0,494	0,17	1
ANXA6	4,66E-05	0,736589	0,343	0,043	1
PCMT1	4,68E-05	0,724813	0,343	0,043	1
SRSF11	4,69E-05	1,52783	0,539	0,234	1
NCF1	4,72E-05	4,372764	0,624	0,255	1
PHF20	4,73E-05	0,741036	0,421	0,106	1
TAOK3	4,77E-05	1,181424	0,466	0,128	1
PXN	4,80E-05	0,358641	0,41	0,085	1
UBE2R2	4,80E-05	2,024579	0,5	0,191	1

NDUFB2	4,80E-05	-1,7477	0,792	0,532	1
AZI2	4,84E-05	3,70306	0,376	0,064	1
TXNIP	4,86E-05	18,5969	0,809	0,638	1
MTRNR2L8	4,90E-05	-1,0164	0,022	0,17	1
RHEB	4,98E-05	0,738445	0,455	0,128	1
ARL5A	5,01E-05	1,966396	0,421	0,106	1
RPS29	5,10E-05	45,04793	0,989	0,957	1
SLC31A2	5,16E-05	2,084166	0,438	0,128	1
EIF4A2	5,18E-05	2,440139	0,573	0,277	1
VEGFA	5,20E-05	0,91934	0,404	0,085	1
MX2	5,22E-05	1,617632	0,399	0,085	1
TYMP	5,27E-05	12,82853	0,927	0,766	1
HBP1	5,31E-05	0,554537	0,309	0,021	1
SELPLG	5,31E-05	0,859259	0,438	0,106	1
SUSD6	5,32E-05	1,328478	0,309	0,021	1
MYO9B	5,34E-05	0,73006	0,494	0,17	1
ZC3H11A	5,36E-05	0,837518	0,309	0,021	1
ZSWIM6	5,37E-05	0,711271	0,309	0,021	1
TTC19	5,37E-05	0,757017	0,309	0,021	1
CNOT2	5,37E-05	0,986811	0,309	0,021	1
JOSD1	5,42E-05	0,883222	0,365	0,064	1
ERGIC1	5,44E-05	1,024694	0,365	0,064	1
STARD7	5,46E-05	0,99567	0,348	0,043	1
DOCK2	5,46E-05	1,190997	0,478	0,149	1
OGDH	5,51E-05	0,853931	0,365	0,064	1
SMAP1	5,53E-05	0,741675	0,275	0	1
UBE2A	5,56E-05	0,577874	0,348	0,043	1
AGTPBP1	5,59E-05	0,846007	0,393	0,085	1
FBXW5	5,60E-05	0,710747	0,275	0	1
GBAS	5,61E-05	0,778485	0,275	0	1
RBMS1	5,62E-05	1,065851	0,522	0,191	1
GOLGA7	5,74E-05	0,509249	0,348	0,043	1
IGBP1	5,75E-05	0,933372	0,438	0,128	1
ARAP1	5,77E-05	0,723405	0,365	0,064	1
LRRK2	5,79E-05	1,167977	0,444	0,128	1
TXNDC17	5,80E-05	1,025277	0,511	0,191	1
SH3TC1	5,85E-05	0,826338	0,337	0,043	1
CCL4L2	5,85E-05	25,86712	0,275	0	1
HEXA	5,90E-05	0,63424	0,365	0,064	1
CMC2	5,90E-05	0,63424	0,365	0,064	1
MORF4L2	5,91E-05	0,618394	0,337	0,043	1
PDCD6IP	5,91E-05	0,747698	0,416	0,106	1
MYDGF	5,99E-05	0,816867	0,489	0,17	1
MEAF6	5,99E-05	0,614675	0,337	0,043	1
NR3C1	6,07E-05	0,850587	0,438	0,128	1
TNRC6B	6,07E-05	0,831408	0,388	0,085	1
COX7A2L	6,19E-05	1,325758	0,607	0,319	1
NDUFA3	6,21E-05	0,647357	0,736	0,426	1
PAG1	6,25E-05	1,071304	0,421	0,106	1
CD300C	6,39E-05	1,148301	0,343	0,043	1

SLTM	6,42E-05	0,651049	0,427	0,106	1
ELMO1	6,59E-05	0,482919	0,27	0	1
ZNF277	6,64E-05	0,498979	0,27	0	1
GRASP	6,64E-05	0,648285	0,27	0	1
CS	6,69E-05	0,55653	0,27	0	1
TRMT112	6,69E-05	0,992283	0,702	0,383	1
NLRP1	6,70E-05	0,482124	0,303	0,021	1
SF3B4	6,73E-05	0,542772	0,303	0,021	1
ROGDI	6,73E-05	0,648937	0,27	0	1
SARNP	6,74E-05	0,825547	0,303	0,021	1
RB1	6,77E-05	0,991383	0,371	0,064	1
UBE2L3	6,80E-05	1,148684	0,528	0,191	1
FAM195B	6,81E-05	0,560758	0,27	0	1
HOOK3	6,84E-05	1,323045	0,343	0,043	1
BRMS1	6,85E-05	0,689253	0,27	0	1
SNRPA	6,88E-05	0,759077	0,388	0,085	1
GPI	6,93E-05	0,733833	0,36	0,064	1
ANKRD12	6,95E-05	1,383538	0,36	0,064	1
HINT1	6,99E-05	5,981897	0,865	0,489	1
SNX6	7,05E-05	1,134869	0,41	0,106	1
PICALM	7,07E-05	1,121065	0,511	0,191	1
MRPL54	7,18E-05	0,929005	0,511	0,213	1
PSMD13	7,25E-05	0,456727	0,343	0,043	1
TAF15	7,36E-05	0,674129	0,331	0,043	1
06. Sep	7,46E-05	0,932421	0,331	0,043	1
RNF181	7,50E-05	1,157573	0,612	0,319	1
PDIA6	7,50E-05	2,219587	0,466	0,149	1
TMEM33	7,53E-05	0,691871	0,331	0,043	1
VAMP3	7,56E-05	0,800212	0,444	0,128	1
CAPZA2	7,64E-05	2,089982	0,663	0,383	1
TRAPPC3	7,72E-05	0,543358	0,388	0,085	1
GSN	7,75E-05	2,416102	0,382	0,085	1
HADHA	7,76E-05	1,97899	0,567	0,277	1
MRPL52	7,83E-05	0,468257	0,59	0,255	1
BST2	8,00E-05	0,482633	0,601	0,277	1
SH3GLB1	8,10E-05	0,819883	0,461	0,149	1
NHP2	8,11E-05	1,412	0,416	0,106	1
EIF2A	8,13E-05	1,274543	0,427	0,128	1
CD9	8,21E-05	2,842122	0,298	0,021	1
ALOX5AP	8,30E-05	2,464207	0,298	0,021	1
BCAS2	8,32E-05	2,304464	0,382	0,085	1
TES	8,33E-05	1,080122	0,427	0,128	1
FAM133B	8,37E-05	0,658805	0,393	0,085	1
MGEA5	8,38E-05	0,565993	0,264	0	1
AIM1	8,38E-05	0,565993	0,264	0	1
TOB1	8,40E-05	1,179552	0,298	0,021	1
PLEKHG2	8,41E-05	1,089147	0,298	0,021	1
FAM21C	8,43E-05	0,580782	0,264	0	1
SNRPA1	8,43E-05	0,586927	0,298	0,021	1
UVRAG	8,45E-05	0,664341	0,298	0,021	1

AC013461.	8,52E-05	0,684395	0,264	0	1
ANAPC16	8,55E-05	1,997393	0,607	0,319	1
BEST1	8,69E-05	1,287642	0,264	0	1
GTF2A2	8,76E-05	1,237586	0,545	0,234	1
CCND3	8,81E-05	1,513493	0,472	0,149	1
CTSL	8,83E-05	10,34331	0,461	0,149	1
SAP30BP	8,87E-05	0,773444	0,382	0,085	1
PMAIP1	8,90E-05	17,81822	0,264	0	1
TGOLN2	8,90E-05	1,237607	0,534	0,234	1
SDHD	8,93E-05	1,600809	0,444	0,128	1
HSPA9	8,94E-05	0,982502	0,404	0,106	1
GAS7	9,26E-05	0,683673	0,365	0,064	1
EMB	9,32E-05	0,723585	0,326	0,043	1
MYCBP2	9,33E-05	0,825018	0,404	0,106	1
PAPOLA	9,34E-05	1,164844	0,545	0,213	1
FBP1	9,38E-05	3,075046	0,399	0,106	1
DHRS4	9,43E-05	0,804578	0,354	0,064	1
IFNAR1	9,52E-05	1,035496	0,326	0,043	1
FOXN3	9,55E-05	1,176408	0,376	0,085	1
U2AF1	9,57E-05	0,627585	0,326	0,043	1
CREBBP	9,63E-05	0,634021	0,354	0,064	1
HK1	9,67E-05	0,550002	0,326	0,043	1
SMS	9,68E-05	0,926397	0,444	0,149	1
LMNA	9,72E-05	14,09613	0,292	0,021	1
LAMP1	9,82E-05	0,933324	0,461	0,149	1
MTDH	9,95E-05	2,529204	0,624	0,362	1
TXN2	0,000101	1,403608	0,427	0,128	1
RAD23A	0,000102	1,030571	0,478	0,191	1
RBMX	0,000102	1,265448	0,478	0,191	1
CCNK	0,000103	0,46442	0,354	0,064	1
SNRPF	0,000103	0,789847	0,522	0,213	1
CDK5RAP3	0,000104	0,545402	0,258	0	1
AKR1B1	0,000104	0,600412	0,258	0	1
ITPK1	0,000105	0,421955	0,292	0,021	1
GLTP	0,000106	0,585713	0,292	0,021	1
PPP1R7	0,000106	0,646361	0,292	0,021	1
ARRDC3	0,000106	0,752228	0,258	0	1
B3GNT5	0,000106	3,864396	0,258	0	1
KPNB1	0,000106	0,721889	0,376	0,085	1
RIN3	0,000107	1,103356	0,472	0,17	1
LRRC25	0,000107	1,073076	0,534	0,234	1
PRPF40A	0,000107	0,987694	0,494	0,191	1
RBBP6	0,000109	0,664239	0,36	0,064	1
TMEM167.	0,000109	1,747609	0,657	0,404	1
COX6C	0,000112	4,362944	0,792	0,638	1
PYGL	0,000113	0,842275	0,348	0,064	1
TBRG1	0,000113	1,060288	0,399	0,106	1
PRELID1	0,000113	0,720231	0,848	0,638	1
CKLF	0,000116	1,201805	0,68	0,404	1
ADAM15	0,000116	0,721999	0,32	0,043	1

WBP2	0,000116	1,33008	0,388	0,085	1
NFKB1	0,000117	0,812831	0,298	0,021	1
SMCHD1	0,000117	1,045068	0,466	0,149	1
CSF1R	0,000118	1,356954	0,5	0,191	1
IL6ST	0,000119	0,648668	0,32	0,043	1
RBM25	0,00012	0,915401	0,489	0,191	1
SUN2	0,000121	0,572766	0,32	0,043	1
DRAP1	0,000121	2,278283	0,629	0,319	1
RSRC2	0,000122	0,934886	0,466	0,17	1
MRPS21	0,000123	1,607161	0,596	0,319	1
COX5B	0,000124	4,772862	0,876	0,745	1
PDCD5	0,000124	0,568096	0,348	0,064	1
STXBP2	0,000124	2,740162	0,764	0,553	1
UQCRFS1	0,000126	2,178252	0,652	0,34	1
SLC12A6	0,000126	0,492821	0,253	0	1
COPS2	0,000128	0,524378	0,253	0	1
WIPF1	0,000128	1,359817	0,517	0,213	1
FAM105A	0,000128	0,580525	0,253	0	1
ARL5B	0,00013	2,900322	0,343	0,064	1
GCC2	0,000131	0,941135	0,287	0,021	1
THAP2	0,000131	1,13723	0,287	0,021	1
HSPA4	0,000132	0,946471	0,287	0,021	1
PRDX3	0,000132	0,957374	0,466	0,17	1
DCAF7	0,000132	0,667161	0,287	0,021	1
AKIRIN1	0,000132	0,496038	0,287	0,021	1
DDX39A	0,000132	0,551773	0,287	0,021	1
AQP9	0,000134	1,251097	0,449	0,149	1
PCNP	0,000134	0,703117	0,399	0,106	1
RNASEH2C	0,000135	0,812134	0,421	0,128	1
HIPK3	0,000135	0,766489	0,421	0,128	1
FLI1	0,000136	1,205028	0,343	0,064	1
HMGN3	0,000138	2,726345	0,59	0,319	1
SERPINB9	0,000139	2,676154	0,511	0,213	1
UGT2B7	0,000139	-0,49219	0,006	0,106	1
KCNAB2	0,000139	1,189828	0,393	0,106	1
DGKZ	0,000139	0,498224	0,382	0,085	1
ARL6IP1	0,000141	1,690321	0,444	0,149	1
QSOX1	0,000141	0,683712	0,371	0,085	1
PCF11	0,000141	0,729448	0,343	0,064	1
ARHGEF1	0,000143	0,870147	0,393	0,106	1
FOS	0,000144	150,6431	0,961	0,915	1
ERP44	0,000146	1,24311	0,393	0,106	1
IL6R	0,000147	0,934243	0,343	0,064	1
HNRNPH3	0,000148	0,848793	0,433	0,128	1
SLC16A3	0,000148	1,451424	0,534	0,234	1
MPV17	0,000149	0,533324	0,315	0,043	1
PRCP	0,00015	1,49898	0,416	0,128	1
UBAP1	0,00015	0,631028	0,315	0,043	1
DOCK8	0,000151	1,856371	0,489	0,191	1
RNF166	0,000151	1,020311	0,315	0,043	1

ABHD5	0,000154	2,29321	0,382	0,106	1
PCYT1A	0,000154	0,454124	0,247	0	1
TXNL1	0,000156	0,750287	0,393	0,106	1
CYLD	0,000156	1,341694	0,343	0,064	1
DYNC1H1	0,000156	0,691525	0,449	0,149	1
CBX3	0,000157	1,776884	0,489	0,191	1
ZDHHC7	0,000159	0,535857	0,343	0,064	1
LAMTOR2	0,000159	0,967983	0,59	0,277	1
UFC1	0,00016	2,022028	0,517	0,213	1
NRIP1	0,00016	0,549413	0,247	0	1
ADCY7	0,000162	0,657279	0,281	0,021	1
LUC7L3	0,000162	0,755569	0,416	0,128	1
KIAA0141	0,000163	0,808738	0,247	0	1
JDP2	0,000164	0,868572	0,281	0,021	1
FLOT2	0,000164	0,490351	0,281	0,021	1
FBXO7	0,000164	0,585467	0,281	0,021	1
MNDA	0,000165	13,82306	0,86	0,638	1
CDC42	0,000168	2,750151	0,854	0,702	1
ADPGK	0,000169	0,740793	0,438	0,149	1
TIMM17B	0,000171	0,702396	0,365	0,085	1
MRPL51	0,000173	0,697368	0,511	0,213	1
PRNP	0,000177	4,882603	0,416	0,128	1
DENND3	0,000177	0,757358	0,309	0,043	1
ADAR	0,000181	0,461392	0,376	0,085	1
PSMD12	0,000187	0,585447	0,348	0,064	1
TLR2	0,00019	1,797413	0,494	0,213	1
SF3B2	0,000193	1,013488	0,41	0,128	1
COPB2	0,000193	0,607279	0,337	0,064	1
MAX	0,000195	0,640627	0,365	0,085	1
TMEM2	0,000195	1,459294	0,242	0	1
LEPROTL1	0,000196	0,977824	0,388	0,106	1
SAT2	0,000197	1,222137	0,612	0,298	1
UQCRQ	0,000197	1,706949	0,753	0,532	1
SEC22B	0,000197	0,501535	0,242	0	1
RBL2	0,000198	0,517301	0,242	0	1
C17orf89	0,000198	0,558943	0,242	0	1
KLHL24	0,000198	0,598919	0,242	0	1
OGFRL1	0,000199	0,926312	0,382	0,106	1
IFRD1	0,000199	2,989192	0,382	0,106	1
EIF1AX	0,000199	2,725223	0,472	0,191	1
STARD4	0,0002	0,548107	0,242	0	1
EIF4E	0,000202	2,095974	0,416	0,128	1
ANKRD10	0,000204	0,65448	0,275	0,021	1
LPCAT2	0,000204	0,480229	0,275	0,021	1
ILF3	0,000205	0,626211	0,449	0,149	1
SLC7A5	0,000206	1,623174	0,242	0	1
IGLV3-1	0,000206	8,818779	0,242	0	1
MOB3A	0,000207	0,612056	0,371	0,085	1
PTK2B	0,000207	1,091427	0,36	0,085	1
NXT1	0,000208	1,340851	0,371	0,085	1

GLRX	0,000211	1,181269	0,669	0,362	1
COX6B1	0,000211	5,869938	0,938	0,872	1
ZMIZ1	0,000213	0,729165	0,315	0,043	1
C1QBP	0,000214	1,086306	0,388	0,106	1
TOMM22	0,000215	1,100652	0,494	0,191	1
NDUFB3	0,000217	0,996359	0,517	0,213	1
ARL6IP5	0,000218	1,557117	0,601	0,298	1
GLIPR2	0,000218	2,11866	0,669	0,362	1
CCT2	0,000218	3,804675	0,303	0,043	1
CACYBP	0,000219	3,839503	0,331	0,064	1
NOL7	0,000219	0,768451	0,36	0,085	1
YTHDC1	0,000219	0,985242	0,36	0,085	1
USP8	0,000219	1,030661	0,36	0,085	1
RASSF2	0,000219	0,686896	0,427	0,128	1
NUFIP2	0,00022	0,910272	0,303	0,043	1
ASPH	0,00022	8,748107	0,303	0,043	1
SSNA1	0,000221	0,858587	0,331	0,064	1
TACC1	0,000221	1,182847	0,421	0,149	1
DAPK1	0,000222	0,994931	0,303	0,043	1
MEGF9	0,000222	0,927039	0,303	0,043	1
CCR1	0,000222	0,93937	0,461	0,17	1
PNPLA6	0,000224	0,634625	0,281	0,021	1
RAP1A	0,000225	1,391884	0,59	0,319	1
TMEM50A	0,000227	0,824316	0,663	0,362	1
EMILIN2	0,000227	1,242688	0,506	0,213	1
CDC42SE1	0,000228	0,721082	0,556	0,234	1
RBP7	0,000229	0,761196	0,433	0,128	1
TWISTNB	0,000233	1,520291	0,331	0,064	1
UBA1	0,000234	0,950457	0,36	0,085	1
LACTB	0,000236	0,481454	0,371	0,085	1
RP11-644F	0,000236	0,394848	0,236	0	1
HGSNAT	0,000236	0,441789	0,236	0	1
POLR2G	0,000236	0,667317	0,427	0,128	1
TMED2	0,000238	0,86789	0,393	0,106	1
ATF6B	0,000238	0,580035	0,331	0,064	1
METTL9	0,000239	1,16035	0,607	0,319	1
FUNDC2	0,00024	0,773245	0,382	0,106	1
FAM21A	0,000241	0,46318	0,236	0	1
IL1RAP	0,000242	0,479558	0,236	0	1
SBDS	0,000244	0,624528	0,331	0,064	1
YME1L1	0,000245	0,979122	0,416	0,128	1
CYC1	0,000246	1,131564	0,427	0,149	1
ZNF267	0,000247	1,37907	0,365	0,085	1
MT1F	0,000249	-7,92494	0,107	0,298	1
MIR181A1	0,000251	2,042929	0,27	0,021	1
GABPB1	0,000252	0,860727	0,27	0,021	1
RNF19B	0,000252	0,839605	0,27	0,021	1
SMC5	0,000254	0,463072	0,27	0,021	1
C1orf52	0,000254	0,414097	0,27	0,021	1
MED10	0,000254	0,474451	0,27	0,021	1

HAVCR2	0,000256	1,898176	0,36	0,085	1
MPP1	0,000259	0,735051	0,365	0,085	1
NPC2	0,000261	8,686954	0,961	0,83	1
EP300	0,000266	0,584635	0,365	0,085	1
COPA	0,000266	1,269724	0,449	0,17	1
PARK7	0,000267	2,095328	0,742	0,553	1
EIF2S2	0,000268	1,343681	0,494	0,191	1
CAPZA1	0,00027	1,074856	0,657	0,362	1
PTPN1	0,00027	0,812399	0,298	0,043	1
TUFM	0,000272	2,634058	0,539	0,277	1
CUEDC2	0,000278	0,692782	0,298	0,043	1
IRF5	0,000282	0,817125	0,298	0,043	1
NAGA	0,000283	0,893091	0,404	0,128	1
NDUFV1	0,000284	0,764122	0,455	0,17	1
HPCAL1	0,000285	0,806266	0,298	0,043	1
FAM208A	0,000285	0,404217	0,275	0,021	1
MED13L	0,000286	0,681529	0,326	0,064	1
SRSF1	0,000286	0,681529	0,326	0,064	1
RXRA	0,000288	0,864128	0,354	0,085	1
LPCAT1	0,00029	0,636033	0,326	0,064	1
C11orf98	0,000291	0,388322	0,23	0	1
EPB41	0,000291	0,435563	0,23	0	1
LSM1	0,000292	0,566242	0,298	0,043	1
YTHDF3	0,000293	0,701553	0,376	0,106	1
PRKCD	0,000294	0,657315	0,326	0,064	1
COX19	0,000294	0,468949	0,23	0	1
PAPD5	0,000294	0,949523	0,23	0	1
PPA1	0,000295	1,504734	0,449	0,17	1
FAM45A	0,000295	0,957298	0,399	0,128	1
PRDX6	0,000296	1,109467	0,579	0,277	1
PSMA5	0,000297	1,553463	0,416	0,149	1
MACF1	0,000297	0,709777	0,354	0,085	1
ZC3H12A	0,0003	0,942689	0,23	0	1
RP6-159A1	0,000301	0,824197	0,23	0	1
IGSF6	0,000303	6,633784	0,64	0,383	1
AP000769.	0,000307	1,813286	0,23	0	1
CTNNA1	0,000308	0,853733	0,421	0,149	1
PPP1CC	0,000308	0,877629	0,421	0,149	1
CKS2	0,000309	10,68226	0,343	0,085	1
ARPC1A	0,000311	0,557953	0,326	0,064	1
TPR	0,000313	0,587929	0,421	0,128	1
CHN2	0,000314	0,607972	0,264	0,021	1
BTN3A2	0,000314	1,333663	0,264	0,021	1
RASGRP4	0,000315	0,390944	0,264	0,021	1
CREG1	0,000318	1,123182	0,421	0,149	1
SLC8A1	0,000321	0,885519	0,303	0,043	1
BCAP31	0,000324	0,722676	0,556	0,255	1
MEF2A	0,000328	0,881585	0,376	0,106	1
PTTG1IP	0,000332	0,763693	0,404	0,128	1
PPM1G	0,000333	0,468863	0,303	0,043	1

HNRNPM	0,00034	0,996043	0,494	0,234	1
MALAT1	0,000342	226,6684	1	1	1
MGST3	0,000342	0,864484	0,433	0,149	1
JARID2	0,000347	1,929713	0,382	0,106	1
MCOLN1	0,000347	0,709164	0,292	0,043	1
SKAP2	0,000348	1,152533	0,461	0,17	1
GCA	0,000349	0,870138	0,629	0,34	1
IL17RA	0,000349	0,8103	0,41	0,128	1
C6orf62	0,000349	1,108714	0,382	0,106	1
NUP214	0,000349	1,038421	0,618	0,34	1
GIMAP1	0,00035	0,783225	0,292	0,043	1
UBR5	0,000358	0,381754	0,225	0	1
IER3IP1	0,000358	0,551051	0,292	0,043	1
RAB8A	0,000358	0,716689	0,404	0,128	1
RP11-22N1	0,00036	0,399509	0,225	0	1
OSTC	0,000361	2,104795	0,551	0,255	1
LGALS8	0,000363	0,52246	0,292	0,043	1
VPS51	0,000363	0,598346	0,292	0,043	1
ADRBK2	0,000363	0,4341	0,225	0	1
AHSA1	0,000363	0,479274	0,225	0	1
RFWD2	0,000363	0,479274	0,225	0	1
PGS1	0,000365	0,467031	0,292	0,043	1
ARGLU1	0,000365	1,307755	0,472	0,191	1
DNAJC25-C	0,000366	0,467534	0,225	0	1
RAP2B	0,000369	0,954254	0,393	0,128	1
MARCKSL1	0,000369	6,029998	0,348	0,085	1
RBX1	0,00037	0,763478	0,73	0,468	1
PHF3	0,00037	0,673729	0,32	0,064	1
BOD1L1	0,00037	0,759059	0,32	0,064	1
PRRC2A	0,00037	0,557386	0,225	0	1
ABCA1	0,000371	0,853509	0,225	0	1
PIAS1	0,000374	0,765683	0,371	0,106	1
SOAT1	0,000374	0,592959	0,32	0,064	1
DARS	0,000375	0,6616	0,32	0,064	1
VSIG4	0,000376	3,012568	0,225	0	1
RER1	0,000377	0,950302	0,348	0,085	1
SCAND1	0,000379	1,291819	0,596	0,34	1
PTGER4	0,000379	3,770344	0,343	0,085	1
STIP1	0,000384	0,749387	0,258	0,021	1
CDKN1B	0,000384	0,749387	0,258	0,021	1
USP7	0,000386	0,554112	0,258	0,021	1
YIPF4	0,000387	0,49851	0,258	0,021	1
MDM2	0,000388	0,925411	0,258	0,021	1
JAZF1	0,000388	0,615998	0,258	0,021	1
STAB1	0,000388	0,89683	0,326	0,064	1
EPRS	0,000389	0,625771	0,258	0,021	1
FURIN	0,000389	0,40171	0,258	0,021	1
AES	0,000394	0,83399	0,343	0,085	1
TCP1	0,000396	0,921197	0,416	0,149	1
CD33	0,000396	1,354053	0,416	0,149	1

PTPN18	0,000401	1,055825	0,371	0,106	1
NUP58	0,000408	0,727557	0,343	0,085	1
JMJD6	0,000409	0,904443	0,343	0,085	1
SET	0,000413	2,040458	0,601	0,34	1
SNX3	0,000417	1,920114	0,713	0,468	1
LAMTOR4	0,00042	1,628939	0,921	0,766	1
PRPF38B	0,000422	0,690954	0,393	0,128	1
ABI3	0,000423	1,401138	0,41	0,149	1
ZFAND2A	0,000425	5,055798	0,315	0,064	1
PRKCSH	0,000426	0,538907	0,376	0,106	1
SPATA13	0,000426	0,932969	0,287	0,043	1
NOP58	0,00043	1,5095	0,287	0,043	1
ICAM2	0,000431	0,715905	0,343	0,085	1
TPD52L2	0,000431	0,662229	0,343	0,085	1
IDH3B	0,000433	0,756957	0,287	0,043	1
MIS18BP1	0,000435	1,018934	0,348	0,085	1
MED30	0,000436	0,535626	0,287	0,043	1
SMCO4	0,000437	1,028212	0,466	0,191	1
RLIM	0,000437	1,398157	0,287	0,043	1
CPSF3L	0,000437	0,356946	0,219	0	1
SND1	0,000439	0,792915	0,343	0,085	1
MAPKAPK3	0,000441	0,604831	0,315	0,064	1
ARHGAP9	0,000443	0,645406	0,287	0,043	1
UFD1L	0,000444	0,410571	0,219	0	1
CH507-42F	0,000444	0,410571	0,219	0	1
LSM6	0,000446	0,596929	0,472	0,191	1
BAX	0,000446	0,974426	0,562	0,298	1
MESDC2	0,000448	0,532284	0,219	0	1
ZNF593	0,00045	0,555977	0,287	0,043	1
PYURF	0,000451	1,410894	0,41	0,149	1
CLDND1	0,000452	0,536616	0,219	0	1
DPH3	0,000454	0,766032	0,315	0,064	1
ZNF655	0,000454	0,713324	0,315	0,064	1
CELF1	0,000456	0,839185	0,399	0,128	1
CCT5	0,000457	1,469776	0,5	0,234	1
BIRC2	0,000459	0,833906	0,315	0,064	1
MTCH1	0,000461	0,390159	0,427	0,149	1
TMEM170I	0,00047	1,163213	0,337	0,085	1
AKAP9	0,000471	0,9127	0,399	0,128	1
POLR2J3	0,000473	0,687205	0,253	0,021	1
MPG	0,000477	0,611608	0,253	0,021	1
PRKDC	0,000478	0,559196	0,253	0,021	1
GPS1	0,00048	0,488471	0,253	0,021	1
COQ10B	0,00048	0,529825	0,253	0,021	1
CLN3	0,00048	0,529825	0,253	0,021	1
TNFRSF10E	0,00048	0,529825	0,253	0,021	1
SMARCA5	0,000482	0,644178	0,315	0,064	1
ITGA5	0,000487	1,607553	0,309	0,064	1
NCF4	0,00049	0,815198	0,393	0,128	1
ATG12	0,000491	0,563021	0,343	0,085	1

G3BP2	0,000497	0,763939	0,337	0,085	1
RNF144B	0,000499	2,796727	0,444	0,191	1
CLTC	0,000499	1,036718	0,449	0,17	1
ATRAID	0,000504	0,67953	0,404	0,128	1
LILRB4	0,000507	0,876193	0,421	0,149	1
HECA	0,000509	0,393676	0,292	0,043	1
HOTAIRM1	0,000513	0,620203	0,32	0,064	1
ARID5A	0,000514	1,402808	0,343	0,085	1
RELT	0,000516	0,77157	0,337	0,085	1
NANS	0,00052	0,624574	0,365	0,106	1
SYF2	0,000525	0,975275	0,579	0,319	1
TFEC	0,00053	0,723697	0,281	0,043	1
LUCAT1	0,000536	2,298962	0,337	0,085	1
ACP1	0,000539	0,680289	0,309	0,064	1
SIRPB1	0,000539	0,794554	0,309	0,064	1
TUG1	0,000542	0,386476	0,213	0	1
MICU2	0,000542	0,433802	0,213	0	1
UTY	0,000544	0,404148	0,213	0	1
DDB1	0,000546	0,467246	0,213	0	1
FAR1	0,000546	0,66824	0,309	0,064	1
CD300LF	0,000548	0,540628	0,281	0,043	1
NEMF	0,000548	0,578608	0,281	0,043	1
C10orf11	0,000548	0,526599	0,213	0	1
TRMT6	0,000548	0,567863	0,213	0	1
NDUFS7	0,000549	1,299773	0,601	0,319	1
PQLC3	0,000552	0,375311	0,348	0,085	1
ATP2A2	0,000552	0,526285	0,281	0,043	1
PPM1F	0,000552	0,597172	0,213	0	1
FAM111A	0,000555	0,652683	0,213	0	1
ABTB1	0,000556	0,511733	0,281	0,043	1
SRRT	0,00056	0,496967	0,281	0,043	1
MAPK6	0,00056	0,704983	0,281	0,043	1
SNAI1	0,000561	1,193051	0,213	0	1
PPP4R3A	0,000563	0,425374	0,348	0,085	1
DCTN2	0,000564	0,440064	0,281	0,043	1
CD69	0,000565	32,81822	0,213	0	1
TAF7	0,000566	0,899287	0,36	0,106	1
EIF4E2	0,000567	0,627877	0,427	0,149	1
TBC1D7	0,000568	0,596406	0,309	0,064	1
NUDT3	0,000575	0,546766	0,309	0,064	1
PNRC2	0,000576	0,99069	0,404	0,149	1
SEC62	0,000577	1,746258	0,629	0,404	1
HSD17B11	0,000578	0,910973	0,494	0,213	1
TRAF3IP3	0,000579	0,813039	0,36	0,106	1
CLINT1	0,000582	0,525775	0,337	0,085	1
BAG1	0,000583	0,716673	0,337	0,085	1
GLA	0,000584	15,78265	0,247	0,021	1
BDP1	0,000584	0,823386	0,247	0,021	1
CASC3	0,000585	0,502565	0,247	0,021	1
ZNF292	0,000587	0,568302	0,247	0,021	1

SZRD1	0,00059	0,605887	0,247	0,021	1
DDA1	0,000591	0,439349	0,247	0,021	1
CCNT1	0,000592	0,4229	0,247	0,021	1
PTDSS1	0,000592	0,450999	0,247	0,021	1
NBN	0,000597	0,465266	0,309	0,064	1
SAR1A	0,000597	0,542903	0,309	0,064	1
MAP2K1	0,000605	0,916154	0,438	0,17	1
IFT20	0,00061	0,843026	0,309	0,064	1
PABPN1	0,000611	0,499784	0,421	0,149	1
FIS1	0,000611	1,410892	0,562	0,298	1
PTBP1	0,000611	0,579084	0,343	0,085	1
HPS1	0,000619	0,705208	0,36	0,106	1
ABI1	0,000622	0,709704	0,315	0,064	1
CAMLG	0,000625	0,792232	0,433	0,17	1
RYBP	0,000626	0,59555	0,315	0,064	1
SCP2	0,000628	0,630385	0,483	0,191	1
DENR	0,000629	0,901978	0,275	0,043	1
SSR3	0,000636	2,175045	0,567	0,34	1
HMOX1	0,000636	18,66811	0,534	0,255	1
RALY	0,00064	1,396123	0,455	0,191	1
OSER1	0,000648	0,707916	0,275	0,043	1
FCGR1A	0,000652	3,674951	0,416	0,149	1
CHTF8	0,000657	0,343343	0,208	0	1
STRA13	0,000657	0,343343	0,208	0	1
PRKACA	0,000658	0,5394	0,275	0,043	1
MLX	0,000659	0,574813	0,36	0,106	1
C6orf1	0,000663	0,379896	0,208	0	1
TIAL1	0,000665	0,642627	0,303	0,064	1
RP11-295G	0,000665	0,397683	0,208	0	1
MESDC1	0,000666	0,824457	0,208	0	1
RAB1A	0,000667	0,978693	0,517	0,255	1
NECAP1	0,000668	0,41516	0,208	0	1
DGUOK	0,000669	0,992044	0,455	0,191	1
ECHDC1	0,00067	0,54959	0,275	0,043	1
EMC3	0,000673	0,527275	0,36	0,106	1
BTG3	0,000673	0,837905	0,208	0	1
MEA1	0,000673	0,628728	0,36	0,106	1
KIAA1551	0,000673	0,924558	0,337	0,085	1
LRPPRC	0,000675	0,551578	0,208	0	1
ZBTB1	0,000675	0,551578	0,208	0	1
TMEM120A	0,000676	0,573551	0,275	0,043	1
06. Mrz	0,000676	0,670512	0,275	0,043	1
ZFR	0,000677	0,525262	0,208	0	1
ANKRD37	0,000679	0,719639	0,208	0	1
FOXP1	0,000683	0,82897	0,354	0,106	1
PIM3	0,000684	0,827747	0,399	0,128	1
GOLGA4	0,000691	0,531334	0,275	0,043	1
BCL2L11	0,000691	0,632272	0,275	0,043	1
FNDC3A	0,000691	1,053377	0,275	0,043	1
NFYC	0,000696	0,434254	0,275	0,043	1

MTMR11	0,000696	0,434254	0,275	0,043	1
STT3B	0,000698	0,582976	0,331	0,085	1
RAB11FIP1	0,000706	1,416257	0,376	0,128	1
RBCK1	0,000715	0,523579	0,36	0,106	1
TRIM44	0,000719	0,620162	0,365	0,106	1
IL18	0,000719	0,799392	0,242	0,021	1
DDX60L	0,000719	1,505916	0,242	0,021	1
G6PD	0,000721	0,483117	0,331	0,085	1
NFAT5	0,000723	0,548491	0,242	0,021	1
SMU1	0,000723	0,587482	0,242	0,021	1
MIIP	0,000724	0,449609	0,242	0,021	1
GMPR2	0,000724	0,449609	0,242	0,021	1
BPTF	0,000724	0,737146	0,41	0,149	1
GGA2	0,000724	0,492567	0,242	0,021	1
PARP8	0,000727	0,416781	0,242	0,021	1
DENND4B	0,000728	0,461141	0,242	0,021	1
RRP7A	0,000729	0,474609	0,303	0,064	1
KAT6A	0,000729	0,44505	0,242	0,021	1
ATXN10	0,000729	0,51382	0,303	0,064	1
ELF2	0,000729	0,58791	0,303	0,064	1
CNOT7	0,000731	0,993493	0,326	0,085	1
NARS	0,000738	0,499573	0,303	0,064	1
FKBP8	0,000746	0,97269	0,64	0,404	1
TUBB	0,000747	10,41947	0,427	0,17	1
PSMC3	0,000757	0,439829	0,331	0,085	1
AKNA	0,000762	0,824508	0,399	0,149	1
OLA1	0,000766	0,779942	0,36	0,106	1
PSMB9	0,000779	2,314824	0,646	0,447	1
CCPG1	0,00078	0,750193	0,27	0,043	1
CITED2	0,00078	2,781935	0,36	0,106	1
C7orf50	0,000792	0,532769	0,309	0,064	1
RCSD1	0,000799	1,200771	0,421	0,17	1
RAP1GDS1	0,000806	0,355041	0,202	0	1
DMXL1	0,000806	0,355041	0,202	0	1
YBX3	0,00081	1,89172	0,652	0,383	1
HCFC1	0,00081	0,373272	0,202	0	1
PLAA	0,00081	0,373272	0,202	0	1
CD58	0,000811	0,818346	0,27	0,043	1
APBB3	0,000813	0,391176	0,202	0	1
AIMP2	0,000813	0,438286	0,202	0	1
CTD-2336(	0,000816	0,49933	0,202	0	1
DNAJC15	0,000818	1,177515	0,444	0,191	1
OXR1	0,000818	0,426052	0,202	0	1
AGO2	0,000821	0,443044	0,202	0	1
TUBB6	0,000821	0,638685	0,202	0	1
ARIH1	0,000832	1,011964	0,27	0,043	1
STK24	0,000832	0,675476	0,27	0,043	1
PEA15	0,000832	2,903835	0,298	0,064	1
SNRNP200	0,000838	0,618058	0,27	0,043	1
TBC1D8	0,000838	0,774238	0,27	0,043	1

SHISA5	0,000841	0,536657	0,404	0,128	1
ZNF330	0,000843	0,68008	0,298	0,064	1
IDH2	0,000845	0,540379	0,27	0,043	1
MRPL43	0,000845	0,540379	0,27	0,043	1
P2RY13	0,000854	3,132765	0,326	0,085	1
STX4	0,000857	0,470806	0,27	0,043	1
PTGS2	0,000859	5,602608	0,326	0,085	1
LY96	0,000866	0,965477	0,601	0,34	1
PSMD9	0,000869	0,481715	0,27	0,043	1
NDUFAB1	0,000869	0,601769	0,556	0,277	1
CD300A	0,000878	1,650137	0,399	0,149	1
EAF1	0,000883	1,296159	0,32	0,085	1
DNAJB9	0,000884	0,596336	0,236	0,021	1
RAD21	0,000884	1,553722	0,348	0,106	1
CNDP2	0,000887	0,676355	0,331	0,085	1
TMOD3	0,000888	0,585601	0,36	0,106	1
GPBP1L1	0,000888	0,522722	0,298	0,064	1
HIST1H1E	0,000889	2,052402	0,236	0,021	1
ELL	0,000891	0,471181	0,236	0,021	1
AGPAT2	0,000893	0,410623	0,236	0,021	1
PXK	0,000893	0,410623	0,236	0,021	1
TMCO1	0,000893	0,894861	0,36	0,106	1
ZNFX1	0,000895	0,393691	0,236	0,021	1
ITPR2	0,000896	0,376467	0,236	0,021	1
DEDD2	0,000896	0,422611	0,236	0,021	1
CHP1	0,000897	0,869476	0,421	0,17	1
SNRK	0,000897	0,523353	0,236	0,021	1
IREB2	0,000897	0,405882	0,236	0,021	1
DGKD	0,000899	0,32294	0,236	0,021	1
MBP	0,000902	0,938128	0,416	0,17	1
SIRPA	0,000904	0,582199	0,331	0,085	1
KDM5A	0,000906	0,463611	0,326	0,085	1
ITGB1BP1	0,00091	0,569778	0,298	0,064	1
RPL41	0,000912	-8,15704	0,994	1	1
GBP2	0,000918	3,063112	0,326	0,085	1
TET2	0,000933	0,586248	0,382	0,128	1
ICAM3	0,000934	0,672376	0,494	0,234	1
GAPT	0,000957	1,046161	0,264	0,043	1
SNX1	0,000959	0,396648	0,242	0,021	1
PRDM1	0,000966	2,153545	0,292	0,064	1
MRPL20	0,000967	0,784739	0,539	0,255	1
PDK4	0,000978	7,877476	0,264	0,043	1
CCDC53	0,00098	0,329552	0,197	0	1
CDK16	0,00098	0,379582	0,197	0	1
MICAL2	0,00098	0,379582	0,197	0	1
KLF9	0,00098	1,555152	0,292	0,064	1
MON2	0,000984	0,348249	0,197	0	1
PLEKHM1	0,000984	0,397375	0,197	0	1
PTRHD1	0,000984	1,020464	0,449	0,191	1
ACTN4	0,000986	0,508052	0,348	0,106	1

USP32	0,000988	0,366603	0,197	0	1
WBSCR22	0,000988	0,366603	0,197	0	1
PRICKLE4	0,000991	0,384627	0,197	0	1
SPHK1	0,000995	0,643528	0,197	0	1
NUS1	0,000998	0,465541	0,197	0	1
RP11-796E	0,000998	0,465541	0,197	0	1
HAUS4	0,000998	0,551314	0,197	0	1
R3HDM4	0,001004	0,719117	0,264	0,043	1
KIAA1109	0,001005	0,470171	0,197	0	1
UQCR10	0,001005	1,542765	0,826	0,66	1
RP11-1000	0,001007	0,570508	0,197	0	1
MATR3.1	0,001008	0,710193	0,197	0	1
FAM96B	0,001012	1,007681	0,528	0,298	1
CHCHD3	0,001012	0,499612	0,264	0,043	1
SLC1A3	0,001014	1,116511	0,197	0	1
PPP2R1A	0,001022	0,547243	0,421	0,17	1
GTF2B	0,001034	1,047336	0,326	0,085	1
SASH3	0,001035	0,563901	0,427	0,17	1
RNF114	0,001036	0,800382	0,393	0,149	1
NKG7	0,001036	34,69088	0,236	0,021	1
STAT2	0,001036	0,358975	0,303	0,064	1
ACER3	0,001037	0,707846	0,292	0,064	1
CAMTA1	0,001037	0,750833	0,416	0,17	1
TIMM10	0,001038	0,990692	0,264	0,043	1
NDFIP1	0,001039	1,395678	0,455	0,213	1
CTSZ	0,001044	0,999436	0,607	0,34	1
DOCK11	0,001046	0,586353	0,264	0,043	1
JAK2	0,001046	0,559432	0,264	0,043	1
RSF1	0,00105	0,718882	0,292	0,064	1
TMEM123	0,001053	1,485504	0,478	0,213	1
IFI16	0,001056	0,666255	0,539	0,255	1
SPTY2D1	0,001061	0,449697	0,264	0,043	1
ZBTB43	0,001062	1,108525	0,298	0,064	1
PRPF4B	0,001064	0,581928	0,292	0,064	1
GNG2	0,001065	1,037277	0,343	0,106	1
NDUFAF3	0,001068	0,605038	0,494	0,234	1
PSME2	0,001069	12,09272	0,702	0,489	1
CATSPER1	0,001081	0,469818	0,23	0,021	1
TBC1D1	0,001081	0,511935	0,23	0,021	1
PCIF1	0,001088	0,437657	0,23	0,021	1
GMIP	0,001088	0,481121	0,23	0,021	1
ADSS	0,001089	0,830257	0,298	0,064	1
MAZ	0,001091	0,42118	0,23	0,021	1
GM2A	0,001091	0,507651	0,23	0,021	1
FGD4	0,001092	0,91737	0,376	0,128	1
YIF1B	0,001092	0,650719	0,292	0,064	1
ECE1	0,001094	0,449327	0,23	0,021	1
HGS	0,001094	0,449327	0,23	0,021	1
LSM14A	0,001095	0,656448	0,388	0,128	1
NKIRAS2	0,001096	0,476703	0,23	0,021	1

PSMC4	0,001099	0,673003	0,32	0,085	1
TAF12	0,0011	0,352416	0,23	0,021	1
MICAL1	0,0011	0,352416	0,23	0,021	1
PSMA3	0,001107	0,711421	0,371	0,128	1
IQGAP2	0,001109	0,767965	0,354	0,106	1
PRDX1	0,001113	9,994672	0,624	0,404	1
PPP1R12A	0,001113	0,685039	0,343	0,106	1
ZFYVE16	0,001115	0,960586	0,343	0,106	1
HIST1H4C	0,00112	1,046191	0,298	0,064	1
SNRPB2	0,001144	1,712823	0,433	0,191	1
UBE2B	0,001146	1,065242	0,567	0,319	1
SRSF4	0,001151	0,835806	0,41	0,17	1
PQBP1	0,001165	0,693039	0,343	0,106	1
ACTN1	0,001168	0,625803	0,326	0,085	1
C15orf48	0,001188	5,44907	0,225	0,021	1
RCC1	0,001194	0,322585	0,191	0	1
TMEM109	0,001194	0,540718	0,191	0	1
CYBRD1	0,001198	0,341411	0,191	0	1
CWC15	0,0012	0,459592	0,326	0,085	1
SDR39U1	0,001203	0,35989	0,191	0	1
USP24	0,001203	0,408461	0,191	0	1
EVI5	0,001203	0,408461	0,191	0	1
CA5B	0,001207	0,378034	0,191	0	1
LATS2	0,001207	0,378034	0,191	0	1
UTP11L	0,001207	0,378034	0,191	0	1
SLC35A4	0,001207	0,425752	0,191	0	1
GZF1	0,00121	0,442749	0,191	0	1
RSL24D1	0,001211	2,054172	0,59	0,383	1
DUSP5	0,001214	0,413363	0,191	0	1
TUBA4A	0,001214	0,503529	0,191	0	1
PROSC	0,001214	0,503529	0,191	0	1
ARFGAP3	0,001214	0,935646	0,315	0,085	1
PAPD4	0,001217	0,43057	0,191	0	1
FHL3	0,001217	0,560826	0,191	0	1
CXorf40B	0,00122	0,492072	0,191	0	1
NDRG1	0,00122	0,788918	0,258	0,043	1
C9orf78	0,00122	0,515991	0,376	0,128	1
ZC3H7A	0,00122	0,534756	0,191	0	1
MIR222HG	0,00122	0,642339	0,191	0	1
SNX18	0,001225	0,669573	0,191	0	1
HCAR3	0,001228	1,002126	0,191	0	1
FXR1	0,001229	0,72103	0,365	0,128	1
ZBTB16	0,00123	1,297942	0,191	0	1
ZFAND3	0,00123	0,585827	0,258	0,043	1
SIPA1L1	0,00123	0,681659	0,258	0,043	1
POLR3GL	0,001234	0,593931	0,287	0,064	1
TAF9	0,001238	0,784182	0,315	0,085	1
SEC63	0,00124	0,494139	0,258	0,043	1
IK	0,001242	0,847808	0,399	0,149	1
DCTN4	0,001252	0,656614	0,258	0,043	1

GAB2	0,001252	0,595556	0,258	0,043	1
PSMD11	0,00126	0,794086	0,315	0,085	1
PSMF1	0,00126	0,659179	0,348	0,106	1
HIPK2	0,001262	0,504798	0,258	0,043	1
KCNE3	0,001263	0,618454	0,258	0,043	1
KHDRBS1	0,001268	1,019819	0,478	0,234	1
AP1B1	0,001283	0,515344	0,258	0,043	1
PSMD1	0,001283	0,617828	0,258	0,043	1
SAP30	0,001284	1,163358	0,225	0,021	1
PSMC6	0,001288	0,590246	0,287	0,064	1
WDR33	0,001293	0,416619	0,258	0,043	1
SPN	0,001293	0,500631	0,258	0,043	1
MCEMP1	0,001304	2,171513	0,365	0,128	1
IKZF1	0,001304	1,554348	0,365	0,128	1
RBBP4	0,00131	0,702114	0,371	0,128	1
GPAT4	0,001312	0,383846	0,258	0,043	1
HK2	0,001317	0,795539	0,225	0,021	1
ZMAT2	0,001322	0,597682	0,438	0,191	1
PHF11	0,001323	0,526419	0,287	0,064	1
EML4	0,001326	1,347858	0,225	0,021	1
FRYL	0,00133	0,517237	0,225	0,021	1
NRAS	0,001334	0,41505	0,225	0,021	1
RNF41	0,001334	0,41505	0,225	0,021	1
MGRN1	0,001334	0,41505	0,225	0,021	1
GTF2F1	0,001338	0,443367	0,225	0,021	1
SYTL3	0,001338	6,786378	0,225	0,021	1
RANBP1	0,001338	0,736355	0,382	0,149	1
AURKAIP1	0,001339	0,697816	0,567	0,34	1
MPHOSPH	0,001341	0,646171	0,287	0,064	1
LPP	0,001344	0,363602	0,225	0,021	1
UBA2	0,001344	0,363602	0,225	0,021	1
TMEM259	0,001347	0,345847	0,225	0,021	1
LUC7L2	0,001347	0,393392	0,225	0,021	1
ITPA	0,001352	0,474795	0,264	0,043	1
ZNF106	0,00137	1,176838	0,41	0,17	1
COMMD7	0,001375	0,561559	0,32	0,085	1
TAP1	0,001376	0,621074	0,287	0,064	1
GNAI3	0,001387	0,685325	0,461	0,213	1
MTMR14	0,001389	0,584746	0,32	0,085	1
DUT	0,001391	0,548937	0,354	0,106	1
CPM	0,001407	1,710269	0,253	0,043	1
RNF138	0,001409	0,454018	0,287	0,064	1
NUCKS1	0,001415	1,324491	0,404	0,17	1
RPL26L1	0,001426	0,345411	0,264	0,043	1
FABP5	0,001426	32,62838	0,287	0,064	1
EPB41L4A-	0,00143	0,323212	0,331	0,085	1
SDHC	0,001433	0,615262	0,371	0,128	1
NDUFC1	0,001442	0,571803	0,506	0,277	1
GAK	0,001444	0,260864	0,23	0,021	1
DIABLO	0,001447	0,296244	0,185	0	1

KLHL18	0,001452	0,315568	0,185	0	1
PHKG2	0,001452	0,315568	0,185	0	1
ERVK3-1	0,001452	0,315568	0,185	0	1
CECR5	0,001458	0,334527	0,185	0	1
ARHGEF6	0,001458	0,364231	0,287	0,064	1
SNU13	0,001461	3,188002	0,579	0,383	1
MTF1	0,001462	0,353132	0,185	0	1
FIP1L1	0,001462	0,353132	0,185	0	1
FGD3	0,001462	0,353132	0,185	0	1
ZBTB11	0,001462	0,448637	0,185	0	1
GSK3B	0,001467	0,371398	0,185	0	1
VHL	0,001467	0,539401	0,185	0	1
SUCO	0,001467	0,419426	0,185	0	1
MTMR10	0,001467	0,419426	0,185	0	1
NUBP1	0,001467	0,419426	0,185	0	1
CTDNEP1	0,001469	0,696355	0,365	0,128	1
RRN3	0,001475	0,406957	0,185	0	1
CGGBP1	0,001475	0,810007	0,32	0,085	1
RAB14	0,001491	0,455707	0,292	0,064	1
HAX1	0,001492	0,986994	0,253	0,043	1
PPP1R11	0,001495	0,634961	0,337	0,106	1
THRAP3	0,001509	0,604931	0,36	0,128	1
NECAP2	0,00151	0,441852	0,292	0,064	1
JTB	0,001511	1,745116	0,579	0,34	1
RAB11A	0,001517	0,746351	0,472	0,234	1
CDC40	0,001518	0,730089	0,309	0,085	1
USP4	0,001528	0,427802	0,292	0,064	1
LARS	0,001532	0,553066	0,253	0,043	1
GPBAR1	0,001532	0,781675	0,253	0,043	1
SP3	0,001545	0,761718	0,253	0,043	1
CCNY	0,001558	0,470852	0,337	0,106	1
HMGN1	0,00156	1,680555	0,551	0,319	1
TBCA	0,001567	1,16028	0,697	0,489	1
TTC7A	0,00157	0,426758	0,253	0,043	1
CBWD2	0,00157	0,426758	0,253	0,043	1
CPQ	0,00157	0,426758	0,253	0,043	1
TTC17	0,00157	0,426758	0,253	0,043	1
PSEN1	0,001571	0,469222	0,253	0,043	1
DDAH2	0,001571	0,848884	0,253	0,043	1
ST3GAL1	0,001571	0,949411	0,253	0,043	1
PFDN1	0,001573	0,779831	0,309	0,085	1
SUPT4H1	0,001575	0,836136	0,646	0,362	1
PIK3AP1	0,001577	0,698928	0,438	0,191	1
SLC16A6	0,001579	1,289177	0,219	0,021	1
CYFIP1	0,001583	0,410671	0,253	0,043	1
ATG16L2	0,001587	0,956413	0,427	0,191	1
NDUFS2	0,001592	0,72469	0,36	0,128	1
UBL7	0,001595	0,394321	0,253	0,043	1
UBE2J2	0,001595	0,438155	0,253	0,043	1
PMPCB	0,001595	0,438155	0,253	0,043	1

TERF2IP	0,001596	0,406287	0,348	0,106	1
RPL13A	0,0016	-9,33163	0,989	1	1
KRT10	0,001605	0,655605	0,427	0,17	1
NIFK	0,001612	0,644879	0,219	0,021	1
DHX36	0,001618	0,459226	0,326	0,085	1
ASGR2	0,001618	0,969401	0,315	0,085	1
BUD31	0,001618	0,514943	0,416	0,17	1
AP2A1	0,001621	0,594827	0,281	0,064	1
ZNF394	0,001624	0,689516	0,219	0,021	1
SNX9	0,001629	0,701544	0,219	0,021	1
HDAC9	0,001629	0,604213	0,219	0,021	1
TYK2	0,001633	0,391919	0,219	0,021	1
NFKBIB	0,001633	0,391919	0,219	0,021	1
CPT1A	0,001633	0,437371	0,219	0,021	1
MRPS35	0,001634	0,480847	0,219	0,021	1
HUS1	0,001638	0,374665	0,219	0,021	1
BAZ1B	0,001642	0,357107	0,219	0,021	1
RPS6KA4	0,001642	0,404132	0,219	0,021	1
PUF60	0,001643	0,545107	0,281	0,064	1
KMT2D	0,001646	0,339235	0,219	0,021	1
COMMD4	0,001646	0,339235	0,219	0,021	1
AP1M1	0,001663	0,452723	0,281	0,064	1
CHCHD5	0,001666	0,671952	0,382	0,149	1
CHIC2	0,001675	0,621672	0,36	0,128	1
CD86	0,001679	1,438322	0,41	0,17	1
OGT	0,001685	0,517304	0,281	0,064	1
ITSN2	0,001692	0,535352	0,376	0,128	1
SDF4	0,001696	0,577653	0,315	0,085	1
SSBP1	0,00171	1,931051	0,528	0,255	1
ERCC1	0,001725	0,555045	0,41	0,17	1
ELP5	0,001737	0,326256	0,258	0,043	1
PCNXL4	0,001758	0,28904	0,18	0	1
TICAM1	0,001764	0,308503	0,18	0	1
TIMM50	0,001764	0,308503	0,18	0	1
C14orf159	0,001764	0,308503	0,18	0	1
CSNK1G2	0,001764	0,35957	0,18	0	1
UBE2Q2	0,00177	0,327594	0,18	0	1
RASSF1	0,001775	0,346328	0,18	0	1
LMAN1	0,001775	0,346328	0,18	0	1
RAB9A	0,001776	0,442454	0,18	0	1
FAM103A1	0,00178	0,364717	0,18	0	1
SLC25A32	0,001781	0,413059	0,18	0	1
EBLN3	0,001789	0,400511	0,18	0	1
STARD3NL	0,001789	2,681334	0,343	0,106	1
HSD17B12	0,001789	0,447192	0,18	0	1
ILF3-AS1	0,001789	0,447192	0,18	0	1
ZPR1	0,00179	0,491792	0,18	0	1
RGS1	0,00179	32,78231	0,213	0,021	1
C1D	0,001798	0,38728	0,309	0,085	1
NADK	0,001799	0,428659	0,309	0,085	1

CYB5D1	0,0018	0,607876	0,18	0	1
GIMAP2	0,001801	0,602889	0,247	0,043	1
CLEC5A	0,001809	1,142785	0,18	0	1
DPYSL2	0,001811	0,972436	0,382	0,149	1
EMP1	0,001812	7,874949	0,18	0	1
CXorf21	0,001821	1,032152	0,247	0,043	1
UBE2N	0,001823	0,691826	0,41	0,17	1
UBAC2	0,001829	0,736822	0,388	0,149	1
AK2	0,001832	0,714279	0,331	0,106	1
ZNF638	0,001836	0,537672	0,247	0,043	1
KCTD20	0,001848	0,749097	0,275	0,064	1
NDUFB10	0,001854	1,284393	0,584	0,383	1
WBP11	0,00187	0,609555	0,331	0,106	1
CARD8	0,00187	0,516223	0,331	0,106	1
ITGAL	0,001871	0,383153	0,287	0,064	1
MKRN1	0,001871	0,753371	0,331	0,106	1
AAK1	0,001887	0,493878	0,247	0,043	1
SLC25A39	0,001887	0,53364	0,247	0,043	1
MGAT4A	0,001888	0,788196	0,247	0,043	1
ST13	0,0019	0,408412	0,511	0,255	1
PKN1	0,001901	0,628587	0,275	0,064	1
CEP350	0,001901	0,662295	0,275	0,064	1
PPM1B	0,001903	0,436795	0,247	0,043	1
NUMA1	0,001903	0,478844	0,247	0,043	1
SECISBP2	0,001903	0,519196	0,247	0,043	1
SPAG7	0,001912	0,742872	0,331	0,106	1
CBWD1	0,001919	0,42087	0,247	0,043	1
ARCN1	0,001919	0,46358	0,247	0,043	1
IGFLR1	0,001924	0,725497	0,303	0,085	1
FAM177A1	0,001931	0,591622	0,36	0,128	1
LAGE3	0,001935	0,689707	0,247	0,043	1
CCDC59	0,001941	0,428005	0,433	0,191	1
SAFB	0,00195	0,388238	0,247	0,043	1
SPIDR	0,00195	0,7942	0,247	0,043	1
ATP6V0A1	0,00195	0,432334	0,247	0,043	1
CHMP5	0,001955	0,778003	0,275	0,064	1
EMC4	0,001957	0,485382	0,303	0,085	1
GAA	0,001958	0,651495	0,303	0,085	1
ALG13	0,001963	0,495048	0,213	0,021	1
CHRAC1	0,001963	0,495048	0,213	0,021	1
UBL3	0,001964	0,371515	0,247	0,043	1
CEBPG	0,001964	0,371515	0,247	0,043	1
MAN2A1	0,001977	0,419457	0,213	0,021	1
GON4L	0,001991	0,385606	0,213	0,021	1
IKBKG	0,001991	0,43134	0,213	0,021	1
SMIM3	0,001991	0,622723	0,213	0,021	1
CHST15	0,001993	0,475695	0,331	0,106	1
HUWE1	0,001997	0,501762	0,213	0,021	1
PHIP	0,002002	0,689459	0,354	0,128	1
GPR108	0,002002	0,350569	0,213	0,021	1

DUSP22	0,002007	0,462183	0,275	0,064	1
CCS	0,002007	0,319596	0,247	0,043	1
ALKBH5	0,002007	0,33258	0,213	0,021	1
BLCAP	0,002007	0,33258	0,213	0,021	1
SUPT5H	0,002007	0,380745	0,213	0,021	1
MTMR3	0,002033	0,447176	0,275	0,064	1
SLC36A4	0,002034	0,487456	0,275	0,064	1
PLCB2	0,002034	0,526175	0,275	0,064	1
MRPS36	0,002035	0,536522	0,399	0,149	1
C15orf39	0,002048	1,063586	0,326	0,106	1
PNKD	0,002053	0,637595	0,382	0,149	1
NCSTN	0,00206	0,472826	0,275	0,064	1
HSF1	0,00206	0,472826	0,275	0,064	1
CHCHD7	0,002061	0,512105	0,275	0,064	1
SERTAD2	0,002061	0,512105	0,275	0,064	1
OTUD1	0,002061	0,936839	0,365	0,128	1
UBE3A	0,002062	0,481405	0,303	0,085	1
HEBP1	0,002063	0,519136	0,303	0,085	1
MPHOSPH	0,002068	0,464441	0,371	0,128	1
FYN	0,002095	0,9348	0,326	0,106	1
HSPA1B	0,002126	19,56155	0,376	0,149	1
NT5C	0,002132	0,452706	0,303	0,085	1
APOA1BP	0,002133	0,281783	0,174	0	1
MAPK8IP3	0,002133	0,281783	0,174	0	1
ACLY	0,002133	0,281783	0,174	0	1
RASA3	0,002146	0,464965	0,174	0	1
AGGF1	0,002146	0,320613	0,174	0	1
RALGAPA1	0,002146	0,496641	0,174	0	1
PVRL2	0,002146	0,371081	0,174	0	1
TMEM261	0,002152	0,339477	0,174	0	1
WBP1L	0,002152	0,339477	0,174	0	1
RIC1	0,002152	0,389025	0,174	0	1
TMEM55A	0,002152	0,389025	0,174	0	1
TPMT	0,002158	0,357991	0,174	0	1
AD000671	0,002158	0,357991	0,174	0	1
SMARCC2	0,002158	0,357991	0,174	0	1
ZMYM6NB	0,002158	0,406652	0,174	0	1
PDE4DIP	0,002158	0,497399	0,174	0	1
ME2	0,002167	1,345544	0,281	0,064	1
DDX50	0,002167	0,394022	0,174	0	1
RP11-386I	0,002168	0,82207	0,174	0	1
HCAR2	0,002168	0,441001	0,174	0	1
CACNA2D4	0,002172	0,573459	0,174	0	1
FAM46A	0,002183	2,069546	0,174	0	1
EGR2	0,002184	0,760071	0,174	0	1
CNOT1	0,002203	0,463108	0,303	0,085	1
RAB3D	0,002203	0,543304	0,281	0,064	1
COPS3	0,002237	0,757458	0,303	0,085	1
GLG1	0,002238	0,892534	0,242	0,043	1
EVL	0,002254	1,081436	0,27	0,064	1

B4GALT1	0,002259	1,311707	0,388	0,149	1
EHD4	0,00228	0,676882	0,242	0,043	1
BUB3	0,002283	0,693325	0,27	0,064	1
CXCL3	0,00229	15,47671	0,208	0,021	1
TMEM208	0,002291	0,584449	0,376	0,149	1
PRPF38A	0,0023	0,488374	0,242	0,043	1
NUDT21	0,002306	0,702106	0,298	0,085	1
URM1	0,00232	0,473255	0,242	0,043	1
ARL6IP4	0,002334	0,93616	0,781	0,553	1
SEL1L	0,00234	0,457905	0,242	0,043	1
MRPL4	0,00234	0,869415	0,242	0,043	1
CLEC12A	0,002345	-0,57783	0,5	0,234	1
ZFC3H1	0,00235	0,669399	0,27	0,064	1
STK17A	0,002352	2,771012	0,247	0,043	1
TSSC4	0,002359	0,442315	0,242	0,043	1
PHKB	0,002359	0,442315	0,242	0,043	1
PDHB	0,002376	0,504755	0,208	0,021	1
SCAMP3	0,002379	0,426479	0,242	0,043	1
MTMR6	0,002379	0,426479	0,242	0,043	1
MAF1	0,002381	0,658174	0,348	0,128	1
PSMD6	0,002382	0,524971	0,27	0,064	1
COMMD9	0,002384	0,745148	0,348	0,128	1
CPNE3	0,002389	0,517962	0,298	0,085	1
CEP170	0,002391	0,589485	0,298	0,085	1
TOR2A	0,002396	0,473715	0,208	0,021	1
RABGEF1	0,002396	0,473715	0,208	0,021	1
PDPK1	0,002405	0,413316	0,208	0,021	1
MTX1	0,002405	0,413316	0,208	0,021	1
ETV3	0,002406	0,569672	0,208	0,021	1
NDUFA7	0,002414	0,441682	0,208	0,021	1
LARP4B	0,002414	0,441682	0,208	0,021	1
GPR132	0,002414	0,441682	0,208	0,021	1
SLC25A1	0,002414	0,484974	0,208	0,021	1
RECQL	0,002415	0,394033	0,242	0,043	1
CYB561D2	0,002415	0,394033	0,242	0,043	1
ATXN1	0,002416	0,548723	0,27	0,064	1
AATF	0,002422	0,379253	0,208	0,021	1
SMARCE1	0,00243	0,361776	0,208	0,021	1
RNPEPL1	0,002433	0,541125	0,298	0,085	1
SORT1	0,002437	0,343989	0,208	0,021	1
SETD3	0,002437	0,39162	0,208	0,021	1
SNN	0,002437	0,39162	0,208	0,021	1
TLR1	0,002437	0,437086	0,208	0,021	1
ASXL2	0,002444	0,495375	0,208	0,021	1
UBAP2L	0,00245	0,307436	0,208	0,021	1
MAGT1	0,00245	0,307436	0,208	0,021	1
SFSWAP	0,00245	0,356797	0,208	0,021	1
IER5	0,002457	1,01396	0,365	0,128	1
TIMP2	0,002464	0,739615	0,455	0,234	1
IMPDH1	0,002474	0,451451	0,298	0,085	1

SLBP	0,002481	0,584557	0,27	0,064	1
EIF4G1	0,002488	0,905369	0,326	0,106	1
PRKAG2-A!	0,002511	1,660037	0,292	0,085	1
ZRANB2	0,002514	0,57129	0,27	0,064	1
AIP	0,002514	0,506878	0,27	0,064	1
H2AFZ	0,002517	2,256064	0,697	0,468	1
ZC3H13	0,002518	0,436768	0,298	0,085	1
IL27RA	0,002518	0,436768	0,298	0,085	1
MCTP1	0,002548	1,014668	0,343	0,128	1
CIB1	0,002568	3,885534	0,68	0,489	1
DRAM2	0,002569	0,952976	0,371	0,149	1
CCBL2	0,002575	0,254331	0,169	0	1
PTPRA	0,002591	0,29422	0,169	0	1
NEK9	0,002599	0,364398	0,169	0	1
MFSD14B	0,002599	0,533487	0,169	0	1
CPD	0,002604	0,819913	0,292	0,085	1
SLC1A5	0,002605	0,332578	0,169	0	1
BRE	0,002605	0,332578	0,169	0	1
CASP9	0,002606	0,382461	0,169	0	1
UQCRHL	0,002606	0,382461	0,169	0	1
HPRT1	0,00261	0,502857	0,27	0,064	1
NOA1	0,002612	0,35122	0,169	0	1
SPG20	0,002612	0,400203	0,169	0	1
BST1	0,002616	0,900536	0,343	0,128	1
05. Mrz	0,002617	0,36952	0,169	0	1
CLMN	0,002622	0,387491	0,169	0	1
MYO5A	0,002623	0,434771	0,169	0	1
CHKA	0,002631	0,468183	0,169	0	1
DEGS1	0,002632	0,511876	0,169	0	1
H1F0	0,002632	0,553739	0,169	0	1
CKAP4	0,002635	0,45428	0,275	0,064	1
PCM1	0,002643	0,528954	0,388	0,149	1
IL2RG	0,002646	2,490145	0,236	0,043	1
WDR26	0,002647	0,432592	0,298	0,085	1
SENP6	0,002648	1,98519	0,298	0,085	1
SNX17	0,002717	0,930784	0,416	0,191	1
NCOA1	0,002725	0,636395	0,275	0,064	1
LASP1	0,00273	0,6219	0,32	0,106	1
CHD9	0,002748	0,650616	0,236	0,043	1
RAC1	0,002757	2,989381	0,815	0,787	1
IDH3G	0,002767	0,679542	0,399	0,17	1
USP16	0,002781	0,306259	0,404	0,17	1
TOX4	0,002787	0,469415	0,32	0,106	1
PPP2R5C	0,00279	0,609917	0,32	0,106	1
GNB4	0,002799	0,62477	0,236	0,043	1
ATF7IP	0,002799	0,659738	0,236	0,043	1
FNIP2	0,002806	0,539976	0,292	0,085	1
CNTRL	0,002808	0,609995	0,292	0,085	1
MRPS5	0,002808	0,724069	0,275	0,064	1
TADA3	0,002824	0,467636	0,236	0,043	1

DYNC1LI1	0,002824	0,467636	0,236	0,043	1
CASS4	0,002843	0,68263	0,202	0,021	1
MECP2	0,002848	0,452198	0,236	0,043	1
NKTR	0,002858	0,526545	0,292	0,085	1
USP34	0,00286	0,697459	0,264	0,064	1
RNF168	0,002872	0,436519	0,236	0,043	1
CENPC	0,002872	0,436519	0,236	0,043	1
PIK3R5	0,002872	0,436519	0,236	0,043	1
EIF2S1	0,002873	0,478579	0,236	0,043	1
IQSEC1	0,002896	0,375961	0,236	0,043	1
CPNE1	0,002896	0,375961	0,236	0,043	1
ATG101	0,002896	0,420589	0,236	0,043	1
FGD5-AS1	0,002896	0,54364	0,236	0,043	1
RIT1	0,002912	0,61889	0,292	0,085	1
TDG	0,002921	0,407137	0,202	0,021	1
ITCH	0,002921	0,451918	0,202	0,021	1
YPEL2	0,002932	0,435676	0,202	0,021	1
RALGDS	0,002933	0,520954	0,202	0,021	1
DENND6B	0,002933	0,626305	0,202	0,021	1
RASA2	0,002942	0,341805	0,236	0,043	1
IL4R	0,002942	0,387949	0,236	0,043	1
SRP54	0,002943	0,372859	0,202	0,021	1
FEM1C	0,002943	0,419166	0,202	0,021	1
HMGN4	0,002943	0,463423	0,202	0,021	1
OSBPL9	0,002962	0,337365	0,202	0,021	1
UBR2	0,002962	0,337365	0,202	0,021	1
PPP2R2A	0,002962	0,385305	0,202	0,021	1
JPX	0,002964	0,37122	0,236	0,043	1
METTL23	0,003013	0,408889	0,208	0,021	1
IRF8	0,003018	2,887437	0,337	0,128	1
HECTD1	0,003033	0,412321	0,32	0,106	1
CCDC85B	0,003044	0,265449	0,399	0,149	1
CD1D	0,003056	0,978213	0,365	0,149	1
SVBP	0,003058	0,310101	0,242	0,043	1
SDF2	0,00306	0,461923	0,264	0,064	1
PDCD2	0,00306	0,501624	0,264	0,064	1
TCERG1	0,003066	0,337912	0,242	0,043	1
PCMTD1	0,003067	0,470943	0,292	0,085	1
ZFAND6	0,003083	0,90625	0,36	0,149	1
NOSIP	0,003099	0,40493	0,264	0,064	1
CCT7	0,003121	0,84674	0,292	0,085	1
XXbac-BPG	0,003125	0,32028	0,163	0	1
PSMG2	0,003129	0,670338	0,365	0,149	1
TRIM38	0,003134	0,65091	0,343	0,128	1
DCTN1	0,003134	0,287001	0,163	0	1
COPS4	0,003134	0,287001	0,163	0	1
TMEM218	0,003134	0,287001	0,163	0	1
STAC3	0,003134	0,287001	0,163	0	1
NGDN	0,003134	0,287001	0,163	0	1
STAT5A	0,003134	0,287001	0,163	0	1

LTV1	0,003134	0,33915	0,163	0	1
C1orf228	0,003142	0,35767	0,163	0	1
HAT1	0,003142	0,35767	0,163	0	1
PIKFYVE	0,003142	0,35767	0,163	0	1
SSH1	0,00315	0,325632	0,163	0	1
EGLN2	0,00315	0,325632	0,163	0	1
SAMD8	0,003157	0,344402	0,163	0	1
TSPYL2	0,003157	0,516628	0,163	0	1
EIF4EBP3	0,003157	0,393713	0,163	0	1
PSMA3-AS	0,003159	0,382503	0,32	0,106	1
RP11-138A	0,003163	0,362827	0,163	0	1
BCAP29	0,003163	0,411258	0,163	0	1
IL10RB-AS1	0,003169	0,428501	0,163	0	1
CCDC6	0,003174	0,445452	0,163	0	1
SQLE	0,003179	0,925196	0,163	0	1
SEMA6B	0,003182	0,521768	0,163	0	1
MS4A4A	0,003192	2,073726	0,163	0	1
ADD1	0,003218	0,400477	0,264	0,064	1
TMED9	0,003232	0,589451	0,36	0,128	1
SPCS2	0,003234	0,323483	0,382	0,149	1
TMEM14B	0,003289	0,647791	0,449	0,234	1
CMIP	0,003309	0,487151	0,382	0,149	1
FDPS	0,003314	0,741095	0,27	0,064	1
GPR65	0,003321	0,876935	0,315	0,106	1
SPINT2	0,003322	0,949507	0,315	0,106	1
SIGIRR	0,003366	0,438136	0,382	0,149	1
STK38L	0,003368	0,532153	0,23	0,043	1
HPSE	0,003385	3,235515	0,292	0,085	1
ACSL4	0,003412	0,900554	0,287	0,085	1
TMBIM1	0,00346	0,402995	0,23	0,043	1
TMEM230	0,003461	0,556438	0,444	0,213	1
HNRNPR	0,003462	0,739584	0,5	0,277	1
NIN	0,003475	0,702132	0,258	0,064	1
RAB5A	0,003496	0,58723	0,32	0,106	1
CHMP4B	0,003517	0,697056	0,466	0,234	1
MARCKS	0,00352	3,497698	0,517	0,298	1
MRPL18	0,003533	0,949506	0,337	0,128	1
TECR	0,003538	0,753548	0,287	0,085	1
IST1	0,003539	0,436012	0,315	0,106	1
GDI1	0,003542	0,400919	0,197	0,021	1
ITGA4	0,003546	1,599844	0,376	0,17	1
LETMD1	0,003549	0,352727	0,23	0,043	1
NOTCH2NL	0,00357	0,366424	0,197	0,021	1
NIPA2	0,00357	0,366424	0,197	0,021	1
NOLC1	0,00357	0,413023	0,197	0,021	1
PLPPR2	0,00357	0,413023	0,197	0,021	1
CEP85L	0,00357	0,607621	0,197	0,021	1
AP3D1	0,003577	0,335393	0,23	0,043	1
YWHAG	0,003588	0,967824	0,371	0,149	1
SLC43A3	0,003595	0,330697	0,197	0,021	1

TRIM28	0,003605	0,317754	0,23	0,043	1
TMED4	0,003606	0,312343	0,197	0,021	1
UTP18	0,003606	0,312343	0,197	0,021	1
SBNO2	0,003606	0,361468	0,197	0,021	1
TNIP1	0,00362	0,638643	0,337	0,128	1
SCLT1	0,00362	0,524726	0,258	0,064	1
TMEM165	0,003621	0,790122	0,382	0,17	1
TMX2	0,003627	0,274592	0,197	0,021	1
SIGLEC7	0,003636	0,255168	0,197	0,021	1
TMEM183A	0,003663	0,479938	0,287	0,085	1
ALDH3B1	0,003663	0,479938	0,287	0,085	1
RABGGTB	0,003668	0,430348	0,258	0,064	1
ANAPC5	0,003705	0,456831	0,292	0,085	1
NRDC	0,003716	0,791585	0,337	0,128	1
DHX9	0,003717	0,414851	0,258	0,064	1
UBE2E3	0,003717	0,414851	0,258	0,064	1
ADD3	0,003728	0,897486	0,287	0,085	1
VDAC3	0,003767	0,481843	0,258	0,064	1
SH3BP1	0,003775	0,587862	0,264	0,064	1
POP5	0,003776	0,313248	0,157	0	1
FAM126B	0,003776	0,259693	0,157	0	1
FAM127B	0,003776	0,259693	0,157	0	1
WBP1	0,003786	0,27973	0,157	0	1
PDLIM5	0,003793	0,490062	0,287	0,085	1
MFSD14A	0,003795	0,299373	0,157	0	1
SREBF2	0,003795	0,299373	0,157	0	1
KIF1B	0,003795	0,350897	0,157	0	1
SH2D3C	0,003795	0,350897	0,157	0	1
SSRP1	0,003803	0,318638	0,157	0	1
TCF12	0,003803	0,318638	0,157	0	1
NOB1	0,003803	0,318638	0,157	0	1
RP11-750H	0,003803	0,318638	0,157	0	1
KIAA0430	0,003803	0,494984	0,157	0	1
MON1B	0,00381	0,337538	0,157	0	1
PLCXD1	0,003811	0,510853	0,157	0	1
FAM195A	0,003811	0,38718	0,157	0	1
RSU1	0,003815	0,779699	0,258	0,064	1
WDR82	0,003815	0,426006	0,258	0,064	1
DUSP3	0,003815	0,426006	0,258	0,064	1
TMX1	0,003816	0,606726	0,258	0,064	1
FPGS	0,003817	0,356088	0,157	0	1
FAM65A	0,003818	0,451324	0,157	0	1
HLA-DQB2	0,003824	0,467896	0,157	0	1
VNN1	0,003848	1,472621	0,157	0	1
FAM192A	0,003863	0,366866	0,258	0,064	1
DNAJA2	0,003867	0,81443	0,382	0,17	1
TRAPPC6A	0,003871	0,48882	0,292	0,085	1
SNF8	0,003891	0,453065	0,292	0,085	1
NQO2	0,003912	0,282904	0,365	0,128	1
LYRM2	0,00395	0,747612	0,264	0,064	1

LAMTOR3	0,003959	0,378567	0,258	0,064	1
THOC7	0,003979	0,435372	0,371	0,149	1
COMMD1	0,003999	0,346011	0,315	0,106	1
PJA2	0,004002	0,592128	0,337	0,128	1
EFHD2	0,00403	0,775835	0,691	0,489	1
IL16	0,004082	0,44511	0,225	0,043	1
IFITM1	0,004094	5,757754	0,197	0,021	1
MPC2	0,00412	0,429318	0,225	0,043	1
NPEPPS	0,004121	0,471675	0,225	0,043	1
CIAO1	0,004122	0,512312	0,225	0,043	1
DDX24	0,004167	0,522471	0,416	0,191	1
PSMB7	0,004171	0,860996	0,382	0,17	1
FNBP1	0,004191	1,11652	0,371	0,17	1
SRGAP2B	0,004198	0,69331	0,225	0,043	1
ADAM8	0,004212	0,847278	0,23	0,043	1
TSC22D2	0,00423	0,487746	0,191	0,021	1
ASH1L	0,004234	0,467368	0,225	0,043	1
VPS13B	0,004249	0,428213	0,191	0,021	1
PRDM2	0,00427	0,363531	0,225	0,043	1
EIF2AK1	0,00427	0,408705	0,225	0,043	1
HADHB	0,004279	0,386271	0,399	0,17	1
BICD2	0,004289	0,439993	0,191	0,021	1
OSCAR	0,004297	0,576381	0,309	0,106	1
MRPS7	0,004306	0,346385	0,225	0,043	1
REV3L	0,004307	0,539083	0,191	0,021	1
LUC7L	0,004323	0,359948	0,191	0,021	1
USP22	0,004323	0,359948	0,191	0,021	1
TMEM248	0,004323	0,359948	0,191	0,021	1
XAF1	0,004324	1,275286	0,191	0,021	1
MRPL11	0,004327	0,32892	0,292	0,085	1
CD300LB	0,004327	0,495102	0,253	0,064	1
HIPK1	0,004328	0,631097	0,253	0,064	1
TLR5	0,00434	0,342127	0,191	0,021	1
DHX30	0,00434	0,342127	0,191	0,021	1
SUPT16H	0,00434	0,43539	0,191	0,021	1
CPSF7	0,004355	0,372554	0,191	0,021	1
DIP2B	0,004384	0,286678	0,191	0,021	1
RAB5B	0,004384	0,286678	0,191	0,021	1
PPP4R2	0,004387	0,455198	0,309	0,106	1
NCBP3	0,004397	0,26749	0,191	0,021	1
PSMC1	0,004404	0,469577	0,315	0,106	1
CCDC12	0,004405	0,368626	0,337	0,128	1
SLC25A13	0,004409	0,341501	0,225	0,043	1
ZNF800	0,004442	0,474713	0,281	0,085	1
NUDCD2	0,004447	0,424675	0,253	0,064	1
TXN	0,004505	2,2124	0,713	0,511	1
TRAF7	0,004507	0,4509	0,253	0,064	1
ILK	0,004508	0,511307	0,36	0,149	1
GRSF1	0,004509	0,69393	0,253	0,064	1
YIPF3	0,00452	0,46037	0,281	0,085	1

RWDD1	0,004553	0,783032	0,489	0,277	1
PIGC	0,004555	0,25222	0,152	0	1
HLA-G	0,004555	0,25222	0,152	0	1
ARID3A	0,004555	0,25222	0,152	0	1
CH507-9B2	0,004555	0,25222	0,152	0	1
DDX1	0,004566	0,272405	0,152	0	1
UGGT1	0,004566	0,272405	0,152	0	1
GSAP	0,004567	0,393257	0,253	0,064	1
COPS5	0,004567	0,393257	0,253	0,064	1
HDAC1	0,004567	0,435721	0,253	0,064	1
TMEM138	0,004576	0,292191	0,152	0	1
CXorf40A	0,004576	0,344077	0,152	0	1
SNRPE	0,004583	0,731864	0,466	0,234	1
RPA2	0,004586	0,362508	0,152	0	1
HNRNPU-A	0,004594	0,330626	0,152	0	1
CCR2	0,004594	0,505045	0,152	0	1
UPF3A	0,004594	0,380604	0,152	0	1
TNFSF8	0,004615	1,139892	0,152	0	1
TNF	0,00462	0,644313	0,152	0	1
PRKAR2A	0,004626	0,37717	0,253	0,064	1
MAN1A2	0,004686	0,404654	0,253	0,064	1
SEC14L1	0,004701	0,717061	0,416	0,213	1
RC3H1	0,004744	0,344199	0,253	0,064	1
TM9SF4	0,004744	0,344199	0,253	0,064	1
CCDC28A	0,004745	0,388751	0,253	0,064	1
SNX5	0,004745	0,709129	0,354	0,149	1
ZDHHC20	0,004779	0,464391	0,365	0,149	1
FAM120A	0,004827	0,506859	0,388	0,17	1
STX16	0,004861	0,356166	0,253	0,064	1
DDX27	0,004896	0,671006	0,219	0,043	1
EMC7	0,004914	0,426852	0,281	0,085	1
BAZ2A	0,004973	0,683027	0,258	0,064	1
NUDC	0,004986	0,629195	0,303	0,106	1
AHCTF1	0,004989	0,506908	0,219	0,043	1
RTCB	0,004989	0,610217	0,219	0,043	1
PRMT9	0,004991	0,79792	0,219	0,043	1
MAML2	0,005018	1,1413	0,247	0,064	1
KDELR2	0,005024	1,130664	0,354	0,149	1
PRKAR1A	0,00503	1,593349	0,517	0,319	1
OPA1	0,005035	0,450585	0,219	0,043	1
TRIP12	0,005038	0,532748	0,326	0,128	1
ACTR1A	0,005046	0,457113	0,287	0,085	1
NDUFS3	0,005053	0,300062	0,287	0,085	1
ALKBH7	0,005073	0,748164	0,472	0,255	1
NELFE	0,005091	0,392629	0,331	0,128	1
ABCF1	0,005126	0,418924	0,219	0,043	1
ATG2A	0,005162	0,493178	0,185	0,021	1
NCOR2	0,005171	0,55562	0,219	0,043	1
PEPD	0,005184	0,776815	0,309	0,106	1
SUPT20H	0,005185	0,433977	0,185	0,021	1

YLPM1	0,005207	0,371051	0,185	0,021	1
N4BP2L1	0,005207	0,371051	0,185	0,021	1
AP1G1	0,005207	0,371051	0,185	0,021	1
LRRFIP2	0,005215	0,386228	0,219	0,043	1
SP140L	0,005228	0,353429	0,185	0,021	1
PPM1M	0,005229	0,518679	0,185	0,021	1
PIGA	0,005229	0,400623	0,185	0,021	1
QARS	0,005237	0,565611	0,247	0,064	1
FNTA	0,005258	0,322445	0,219	0,043	1
KDM7A	0,005259	0,414382	0,219	0,043	1
AFG3L2	0,005269	0,317225	0,185	0,021	1
RPIA	0,005269	0,317225	0,185	0,021	1
OSGEP	0,005269	0,317225	0,185	0,021	1
DCAF8	0,005269	0,366118	0,185	0,021	1
THOC6	0,005269	0,366118	0,185	0,021	1
NRBP1	0,00528	0,483674	0,275	0,085	1
AGO3	0,005288	0,29862	0,185	0,021	1
CBL	0,005288	0,29862	0,185	0,021	1
DERA	0,005288	0,29862	0,185	0,021	1
CASC4	0,005288	0,348408	0,185	0,021	1
NUBP2	0,005301	0,304573	0,219	0,043	1
DNAJC4	0,005302	0,657677	0,427	0,213	1
ATG5	0,005306	0,279662	0,185	0,021	1
PRKD3	0,005306	0,330379	0,185	0,021	1
GNL3	0,005306	0,330379	0,185	0,021	1
DCP2	0,005309	0,575243	0,326	0,128	1
IFNAR2	0,005309	0,66092	0,326	0,128	1
PPP4R3B	0,00531	0,552088	0,247	0,064	1
SMIM4	0,00531	0,588428	0,247	0,064	1
MAP4K4	0,005311	0,623494	0,247	0,064	1
SF3B5	0,005327	1,705237	0,635	0,468	1
TROVE2	0,005341	0,36336	0,225	0,043	1
RUNX3	0,005341	1,186296	0,225	0,043	1
GALK1	0,005373	0,429769	0,275	0,085	1
MKKS	0,005374	0,46946	0,275	0,085	1
SIRT2	0,005384	0,267843	0,219	0,043	1
HERC4	0,005385	0,263408	0,191	0,021	1
BLOC1S1	0,005439	-0,63767	0,444	0,638	1
EFCAB14	0,00544	0,457774	0,326	0,128	1
NME3	0,005443	0,529183	0,326	0,128	1
LIPA	0,005449	3,588307	0,382	0,17	1
SNRPD1	0,005451	0,361514	0,393	0,17	1
NXF1	0,005454	0,445343	0,247	0,064	1
RCOR1	0,005454	0,445343	0,247	0,064	1
C20orf27	0,005467	1,148513	0,404	0,213	1
APOBR	0,005467	0,414761	0,275	0,085	1
KIAA0922	0,00549	0,299033	0,146	0	1
WDR6	0,005502	0,265027	0,146	0	1
FAR2	0,005502	0,265027	0,146	0	1
ZNF121	0,005502	0,265027	0,146	0	1

TDP2	0,005502	0,265027	0,146	0	1
RTCA	0,005502	0,318304	0,146	0	1
U2AF1L5	0,005513	0,284958	0,146	0	1
VASH1	0,005513	0,337211	0,146	0	1
SIGLEC14	0,005513	0,337211	0,146	0	1
SH3PXD2B	0,005513	0,337211	0,146	0	1
GTF3C2	0,005523	0,3045	0,146	0	1
USP39	0,005523	0,3045	0,146	0	1
ZNF333	0,005523	0,355767	0,146	0	1
PFDN4	0,005527	0,430079	0,247	0,064	1
LINC00877	0,005528	0,700813	0,247	0,064	1
LINC00969	0,005532	0,421892	0,146	0	1
DDHD1	0,005532	0,541588	0,146	0	1
ATXN7L3B	0,00554	0,55674	0,146	0	1
FMN1	0,005547	0,530562	0,146	0	1
PLK2	0,005566	0,952033	0,146	0	1
IFI27L2	0,005582	0,953992	0,539	0,362	1
HDLBP	0,005597	0,833825	0,365	0,17	1
DYNLL1	0,005702	0,396531	0,674	0,489	1
INPP5D	0,005707	0,559321	0,281	0,085	1
SUMO1	0,005707	1,360179	0,517	0,34	1
FDX1	0,005715	1,969617	0,303	0,106	1
GRK6	0,005743	0,338014	0,247	0,064	1
FUCA2	0,005754	0,410493	0,275	0,085	1
FAM107B	0,005764	0,391567	0,281	0,085	1
CREB5	0,005796	0,687041	0,343	0,149	1
WDR61	0,005815	0,410168	0,247	0,064	1
TRA2A	0,00582	0,906849	0,444	0,234	1
BFAR	0,00583	0,349951	0,253	0,064	1
ORMDL1	0,005864	0,453931	0,326	0,128	1
RAB6A	0,005888	0,50011	0,298	0,106	1
PPA2	0,005906	0,334538	0,253	0,064	1
HNMT	0,005911	0,428556	0,478	0,255	1
TMEM243	0,005915	0,605952	0,213	0,043	1
SSU72	0,005936	0,749421	0,449	0,255	1
ZBTB7A	0,005937	0,2914	0,253	0,064	1
FOXN2	0,005973	0,854664	0,213	0,043	1
CLIP1	0,005981	0,429154	0,253	0,064	1
SNX27	0,006003	0,582664	0,275	0,085	1
SEC61A1	0,006006	0,746409	0,281	0,085	1
GYPC	0,006029	0,731879	0,213	0,043	1
TMEM154	0,006042	0,658138	0,27	0,085	1
07. Mrz	0,006043	0,475014	0,275	0,085	1
EEA1	0,006142	0,512056	0,213	0,043	1
PARP14	0,00617	0,708997	0,36	0,149	1
LARP1	0,006196	0,412989	0,213	0,043	1
CHTOP	0,006196	0,412989	0,213	0,043	1
JAK3	0,006197	0,497294	0,213	0,043	1
IDH3A	0,006202	0,502902	0,18	0,021	1
SIGLEC10	0,006233	1,471373	0,18	0,021	1

COX14	0,006234	0,432346	0,517	0,298	1
IAH1	0,006262	0,624802	0,27	0,085	1
QPCT	0,006281	0,624271	0,298	0,106	1
PRDX4	0,006288	0,455885	0,18	0,021	1
SNAP23	0,006295	0,507466	0,371	0,17	1
U2SURP	0,006295	0,565248	0,376	0,17	1
TTC14	0,006305	0,333579	0,213	0,043	1
TFAM	0,006305	0,424544	0,213	0,043	1
GALNT2	0,006314	0,346867	0,18	0,021	1
KLHDC3	0,006314	0,346867	0,18	0,021	1
RELA	0,006314	0,346867	0,18	0,021	1
ST14	0,006315	0,513119	0,18	0,021	1
COG3	0,006315	0,394365	0,18	0,021	1
VIM-AS1	0,006323	0,560658	0,242	0,064	1
FAM126A	0,00634	0,32881	0,18	0,021	1
ING3	0,00634	0,32881	0,18	0,021	1
EHMT1	0,00634	0,32881	0,18	0,021	1
NARF	0,00634	0,32881	0,18	0,021	1
CDK11A	0,006341	0,497849	0,18	0,021	1
LMNB1	0,006341	0,377153	0,18	0,021	1
UGP2	0,006358	0,315907	0,213	0,043	1
EIF2AK2	0,006358	0,315907	0,213	0,043	1
CDC123	0,006359	0,363234	0,213	0,043	1
RNF115	0,006365	0,310421	0,18	0,021	1
UBE3C	0,006389	0,291687	0,18	0,021	1
PDHA1	0,006389	0,291687	0,18	0,021	1
NPIPB5	0,006389	0,291687	0,18	0,021	1
SLC25A36	0,006389	0,341813	0,18	0,021	1
ARHGEF2	0,006411	0,297918	0,213	0,043	1
POLR2B	0,006411	0,297918	0,213	0,043	1
PISD	0,006411	0,297918	0,213	0,043	1
LINC00937	0,006412	0,272596	0,18	0,021	1
CINP	0,006412	0,272596	0,18	0,021	1
CRLS1	0,006412	1,241222	0,18	0,021	1
ADRM1	0,006459	0,633409	0,371	0,17	1
TPRKB	0,006461	0,534466	0,275	0,085	1
ABCE1	0,006463	0,328632	0,213	0,043	1
YTHDF2	0,006496	0,413231	0,242	0,064	1
PIK3R1	0,006499	0,49485	0,242	0,064	1
TMEM43	0,006514	0,310872	0,213	0,043	1
ATP1B1	0,006555	-0,64066	0,079	0,213	1
ABHD14B	0,006586	0,546417	0,242	0,064	1
PRRC2B	0,006587	0,480328	0,242	0,064	1
GNA15	0,006587	0,480328	0,242	0,064	1
EVI2A	0,006589	0,503109	0,41	0,191	1
SURF1	0,0066	0,409183	0,27	0,085	1
TNFAIP8	0,006603	0,621344	0,27	0,085	1
TSPAN3	0,006607	0,427091	0,14	0	1
PLBD2	0,006607	0,291849	0,14	0	1
IGF2R	0,006617	0,26386	0,219	0,043	1

RFX5	0,006621	0,257593	0,14	0	1
TP53BP2	0,006621	0,257593	0,14	0	1
CPSF3	0,006621	0,257593	0,14	0	1
C1GALT1	0,006621	0,257593	0,14	0	1
THAP7	0,006621	0,257593	0,14	0	1
LINC00998	0,006621	0,311258	0,14	0	1
AGPS	0,006621	0,311258	0,14	0	1
KCNMB1	0,006621	0,311258	0,14	0	1
COX7A2	0,006631	1,68482	0,809	0,66	1
SETD7	0,006633	0,277672	0,14	0	1
ZNF791	0,006633	0,277672	0,14	0	1
TMEM256-	0,006633	0,277672	0,14	0	1
MEMO1	0,006633	0,330297	0,14	0	1
EXOSC10	0,006643	0,297355	0,14	0	1
DNAJB2	0,006643	0,297355	0,14	0	1
MRPS10	0,006643	0,297355	0,14	0	1
FLT3	0,006643	0,297355	0,14	0	1
UBE2M	0,006643	0,297355	0,14	0	1
ICT1	0,006643	0,297355	0,14	0	1
DIMT1	0,006644	0,477173	0,14	0	1
PPARD	0,006644	0,34898	0,14	0	1
SELO	0,006644	0,34898	0,14	0	1
RHOT2	0,006653	0,316658	0,14	0	1
MYC	0,006654	0,77233	0,14	0	1
SLC12A7	0,006654	0,367321	0,14	0	1
ZNF280D	0,006654	0,367321	0,14	0	1
FOSL1	0,006662	0,335596	0,14	0	1
CCND2	0,006671	0,670622	0,14	0	1
MYLIP	0,006671	0,449591	0,14	0	1
RBM42	0,006674	0,381446	0,242	0,064	1
AC058791.	0,006675	0,372428	0,14	0	1
MAPK7	0,006677	0,466191	0,14	0	1
H1FX	0,006682	3,761675	0,416	0,191	1
MTHFS	0,006683	0,415939	0,298	0,106	1
ILF2	0,006714	0,683575	0,343	0,149	1
CEBPB	0,006716	1,876095	0,826	0,617	1
TPST2	0,006762	0,518651	0,242	0,064	1
HNRNPAB	0,006841	0,344725	0,303	0,106	1
KRAS	0,006873	0,640571	0,371	0,17	1
SREK1IP1	0,006905	0,359573	0,247	0,064	1
BAZ1A	0,006925	1,367397	0,416	0,234	1
CDK2AP2	0,00693	0,61233	0,326	0,128	1
KARS	0,006937	0,33179	0,242	0,064	1
PRKAG2	0,006944	0,578598	0,292	0,106	1
AP2B1	0,007063	0,346435	0,27	0,085	1
RAB11B	0,007094	0,495148	0,292	0,106	1
BRD4	0,007109	0,691122	0,343	0,149	1
COPS6	0,007157	0,357266	0,275	0,085	1
GSDMD	0,007162	-0,34043	0,287	0,085	1
HNRNPH1	0,007163	0,765119	0,5	0,277	1

NEU1	0,007204	1,201129	0,208	0,043	1
ACP5	0,007205	1,639622	0,208	0,043	1
SCIMP	0,007214	0,795106	0,427	0,213	1
BSG	0,007221	0,879284	0,567	0,383	1
CNIH1	0,007221	0,76683	0,433	0,213	1
YY1	0,007245	0,566768	0,32	0,128	1
SRPK2	0,007253	0,754865	0,292	0,106	1
MED15	0,007284	0,261497	0,242	0,064	1
BZW2	0,00734	0,439053	0,208	0,043	1
DNM1L	0,00734	0,439053	0,208	0,043	1
PPCS	0,007348	0,64988	0,298	0,106	1
FDFT1	0,007353	0,79932	0,27	0,085	1
LSM4	0,007383	0,515091	0,404	0,191	1
STK38	0,007388	0,325968	0,303	0,106	1
TSC22D4	0,007401	0,327347	0,275	0,085	1
JMY	0,00741	0,645464	0,208	0,043	1
KIAA0513	0,007477	0,40702	0,208	0,043	1
CTNND1	0,007477	0,55932	0,208	0,043	1
WTAP	0,007506	0,565375	0,449	0,234	1
DNAJC3	0,007515	1,033011	0,315	0,128	1
BTF3L4	0,007537	0,64084	0,427	0,234	1
SDE2	0,007548	0,508253	0,174	0,021	1
PPP6R3	0,007582	0,405094	0,174	0,021	1
GRIPAP1	0,00761	0,327114	0,208	0,043	1
FADS1	0,00761	0,327114	0,208	0,043	1
GMFB	0,00761	0,327114	0,208	0,043	1
EDEM2	0,007615	0,340262	0,174	0,021	1
SOX4	0,007616	0,650786	0,174	0,021	1
SYNE3	0,007616	0,433691	0,174	0,021	1
TSPYL1	0,007616	0,433691	0,174	0,021	1
GLMP	0,007647	0,322084	0,174	0,021	1
LRP10	0,007647	0,322084	0,174	0,021	1
SERPINB8	0,007671	0,273052	0,18	0,021	1
SGK3	0,007679	0,30357	0,174	0,021	1
ATXN7	0,007679	0,353118	0,174	0,021	1
ASNA1	0,007706	0,511342	0,298	0,106	1
APEH	0,007709	0,284706	0,174	0,021	1
MALSU1	0,007709	0,284706	0,174	0,021	1
THYN1	0,007709	0,284706	0,174	0,021	1
ACTR10	0,007709	0,284706	0,174	0,021	1
RRAGA	0,007709	0,460734	0,174	0,021	1
TFG	0,007709	0,335174	0,174	0,021	1
CBWD5	0,007713	0,279446	0,213	0,043	1
RPL7L1	0,007722	0,423071	0,236	0,064	1
COPS7A	0,007723	0,268087	0,18	0,021	1
TPP2	0,007725	0,503922	0,236	0,064	1
TAOK1	0,007726	0,514386	0,292	0,106	1
ACOT8	0,007738	0,26548	0,174	0,021	1
NAA15	0,007738	0,26548	0,174	0,021	1
TAB2	0,007738	0,26548	0,174	0,021	1

NAA60	0,007738	0,26548	0,174	0,021	1
PREB	0,007738	0,316903	0,174	0,021	1
SERINC5	0,007739	0,680938	0,213	0,043	1
DYM	0,00774	0,385937	0,208	0,043	1
SP1	0,00774	0,339698	0,208	0,043	1
DPM3	0,007752	0,471793	0,326	0,128	1
COPG1	0,007804	0,272774	0,208	0,043	1
HSD17B10	0,007818	0,47524	0,348	0,149	1
RHBDF2	0,007831	0,555035	0,236	0,064	1
APPL1	0,007833	0,626237	0,236	0,064	1
FGD2	0,007846	0,841101	0,337	0,149	1
ANKRD11	0,00788	0,548221	0,32	0,128	1
CORO1C	0,007884	0,828807	0,365	0,17	1
VPS36	0,007936	0,391602	0,236	0,064	1
RPS6KB2	0,007938	0,434135	0,236	0,064	1
DIAPH2	0,007951	0,547282	0,264	0,085	1
COA4	0,007952	0,483427	0,264	0,085	1
POLDIP2	0,007958	0,250104	0,135	0	1
RAB4B	0,007958	0,250104	0,135	0	1
KIAA0196	0,007958	0,250104	0,135	0	1
PPP2R4	0,007958	0,250104	0,135	0	1
XPR1	0,007958	0,250104	0,135	0	1
PYGB	0,007958	0,250104	0,135	0	1
HOOK2	0,007958	0,250104	0,135	0	1
TXNRD2	0,007958	0,250104	0,135	0	1
NBPF15	0,007958	0,304161	0,135	0	1
ARHGEF40	0,007958	0,304161	0,135	0	1
RP11-345J4	0,007971	0,270332	0,135	0	1
SNIP1	0,007971	0,270332	0,135	0	1
MR1	0,007971	0,270332	0,135	0	1
PDZD8	0,007971	0,270332	0,135	0	1
NAA16	0,007971	0,270332	0,135	0	1
TOPORS-AS	0,007971	0,270332	0,135	0	1
ZHX1	0,007972	0,323335	0,135	0	1
VAMP4	0,007983	0,290159	0,135	0	1
AFF1	0,007983	0,290159	0,135	0	1
ABCB7	0,007983	0,290159	0,135	0	1
RC3H2	0,007983	0,290159	0,135	0	1
DDX52	0,007983	0,290159	0,135	0	1
GSTM1	0,007983	0,290159	0,135	0	1
GDAP2	0,007983	0,471165	0,135	0	1
IRAK2	0,007983	0,342148	0,135	0	1
ATL3	0,007983	0,342148	0,135	0	1
PLA2G7	0,007994	0,40915	0,135	0	1
SESN1	0,007994	0,530292	0,135	0	1
MID1IP1	0,007996	0,960518	0,287	0,106	1
LCOR	0,008003	0,328671	0,135	0	1
ADNP2	0,008003	0,328671	0,135	0	1
HLA-DOA	0,008011	0,396552	0,135	0	1
ID1	0,008021	0,782085	0,135	0	1

VCL	0,008044	0,418698	0,236	0,064	1
CDC5L	0,008045	0,460117	0,236	0,064	1
CH25H	0,00805	13,81856	0,135	0	1
SERPINB6	0,008063	0,452414	0,416	0,213	1
TLE3	0,008083	0,759476	0,354	0,17	1
SPNS1	0,008091	0,637815	0,264	0,085	1
TMEM127	0,00815	0,359111	0,236	0,064	1
DENND5A	0,008151	0,403018	0,236	0,064	1
B3GAT3	0,008151	0,445078	0,236	0,064	1
MTHFD2L	0,008152	0,551203	0,236	0,064	1
MMADHC	0,008161	0,369103	0,242	0,064	1
BAG3	0,00817	9,081181	0,202	0,043	1
RAD23B	0,008229	0,493516	0,264	0,085	1
ETFA	0,008254	0,691248	0,404	0,191	1
PPP2R5E	0,008257	0,34246	0,236	0,064	1
GRPEL1	0,008299	0,47904	0,27	0,085	1
TET3	0,008363	0,325528	0,236	0,064	1
SRP9	0,008377	1,454963	0,511	0,34	1
PAPSS1	0,008508	0,340461	0,264	0,085	1
PBRM1	0,008575	0,290778	0,236	0,064	1
EIF2AK3	0,008591	0,777656	0,202	0,043	1
F13A1	0,008825	3,625259	0,382	0,191	1
VTI1B	0,008826	0,494696	0,343	0,149	1
JAGN1	0,008924	0,417256	0,202	0,043	1
FYTTD1	0,008924	0,417256	0,202	0,043	1
GDE1	0,008925	0,460117	0,202	0,043	1
DENND4C	0,008948	0,420404	0,169	0,021	1
HSPA1A	0,008997	107,1379	0,517	0,383	1
RNF139	0,009006	0,355483	0,202	0,043	1
PKN2	0,009006	0,401014	0,202	0,043	1
TIMM23	0,009006	0,401014	0,202	0,043	1
LSM8	0,009037	0,982619	0,522	0,34	1
DYNLT1	0,009055	2,486834	0,646	0,404	1
EDEM3	0,009088	0,338197	0,202	0,043	1
ACYP2	0,009088	0,338197	0,202	0,043	1
RPS6KA1	0,009088	0,500512	0,202	0,043	1
SETD2	0,009129	0,351585	0,169	0,021	1
ZBTB38	0,009129	0,351585	0,169	0,021	1
OCIAD1	0,009144	0,959638	0,404	0,213	1
CUL3	0,009169	0,320608	0,202	0,043	1
SPG7	0,009169	0,320608	0,202	0,043	1
CHMP1A	0,009169	0,320608	0,202	0,043	1
RNF135	0,009169	0,320608	0,202	0,043	1
EXOSC5	0,009169	0,367717	0,202	0,043	1
YTHDC2	0,009169	0,412707	0,202	0,043	1
GAPVD1	0,009169	0,412707	0,202	0,043	1
ADSL	0,009173	0,427637	0,169	0,021	1
ADM	0,009173	0,964179	0,169	0,021	1
HDAC3	0,009212	0,315313	0,169	0,021	1
MPDU1	0,009212	0,315313	0,169	0,021	1

IWS1	0,009212	0,364297	0,169	0,021	1
FAM162A	0,009212	0,364297	0,169	0,021	1
WDR45	0,009217	0,456678	0,208	0,043	1
XPO6	0,009228	0,506207	0,258	0,085	1
ARF6	0,009243	1,133576	0,534	0,362	1
CLCN7	0,009249	0,302703	0,202	0,043	1
ZNF644	0,009249	0,350643	0,202	0,043	1
SHMT2	0,009249	0,39639	0,202	0,043	1
GNL2	0,009251	0,296672	0,169	0,021	1
KBTBD2	0,009251	0,346554	0,169	0,021	1
AAED1	0,009251	0,346554	0,169	0,021	1
RANBP9	0,009289	0,277676	0,169	0,021	1
PPIL4	0,009289	0,277676	0,169	0,021	1
GSR	0,009289	0,277676	0,169	0,021	1
ERGIC2	0,009289	0,277676	0,169	0,021	1
MRPL38	0,009325	0,258313	0,169	0,021	1
GABBR1	0,009325	0,310096	0,169	0,021	1
CPNE8	0,009325	0,310096	0,169	0,021	1
PRKAG1	0,009328	0,284472	0,202	0,043	1
EAPP	0,009328	0,333273	0,202	0,043	1
FAM53C	0,009328	0,333273	0,202	0,043	1
NFATC2IP	0,009406	0,265902	0,202	0,043	1
PLIN3	0,009447	1,136486	0,32	0,128	1
MAP3K11	0,009546	0,385704	0,23	0,064	1
PDLIM7	0,00955	0,508915	0,23	0,064	1
TIMM17A	0,009557	0,478221	0,258	0,085	1
ACOT9	0,009557	0,577936	0,258	0,085	1
SLC19A2	0,009571	0,262938	0,129	0	1
ATP2C1	0,009571	0,262938	0,129	0	1
ODF2	0,009571	0,262938	0,129	0	1
SMC4	0,009583	0,282911	0,129	0	1
POM121C	0,009583	0,282911	0,129	0	1
DCTPP1	0,009583	0,282911	0,129	0	1
RP11-426C	0,009583	0,282911	0,129	0	1
ACAT2	0,009584	0,46512	0,129	0	1
KIAA0226L	0,009595	0,35386	0,129	0	1
PCNX	0,009604	0,321697	0,129	0	1
NOC2L	0,009605	0,420107	0,129	0	1
LHFPL2	0,009606	2,049502	0,129	0	1
TNRC18	0,009613	0,390037	0,129	0	1
DNAAF1	0,00963	1,0728	0,129	0	1
MPLKIP	0,009646	0,446887	0,292	0,106	1
SERPINB2	0,00965	8,868155	0,129	0	1
CAMK1	0,009673	0,755437	0,287	0,106	1
KPNA4	0,009674	0,482476	0,287	0,106	1
ZSCAN16-A	0,009676	0,369494	0,23	0,064	1
SNRPC	0,009729	0,394093	0,421	0,213	1
MAPRE2	0,009795	0,87502	0,281	0,106	1
NPTN	0,009805	0,397187	0,23	0,064	1
ANKRD13D	0,009805	0,397187	0,23	0,064	1

LMBRD1	0,009806	0,439488	0,23	0,064	1
PRDX5	0,009843	1,297486	0,657	0,511	1
LEPROT	0,009918	0,503582	0,382	0,191	1
NSL1	0,009933	0,336264	0,23	0,064	1
SMIM19	0,009933	0,336264	0,23	0,064	1
DPEP2	0,009933	0,336264	0,23	0,064	1
RPS19BP1	0,009951	0,635458	0,331	0,149	1
MRPL57	0,010016	0,527374	0,433	0,213	1
ATG7	0,010058	0,434715	0,258	0,085	1
TMEM179I	0,010095	0,568907	0,393	0,191	1
HSD17B4	0,010155	0,427689	0,236	0,064	1
USP10	0,010155	0,317248	0,236	0,064	1
NDUFV3	0,010202	0,511465	0,309	0,128	1
PURB	0,010232	0,757617	0,281	0,106	1
PSMB2	0,01024	0,748189	0,455	0,277	1
MTIF3	0,010277	0,470076	0,343	0,149	1
GINM1	0,010286	0,30502	0,264	0,085	1
PEF1	0,010318	0,284253	0,23	0,064	1
NSFL1C	0,010319	0,376603	0,23	0,064	1
ANPEP	0,010337	7,59515	0,32	0,149	1
ARAF	0,01034	0,25669	0,236	0,064	1
CAPN1	0,01036	0,371642	0,264	0,085	1
MAT2B	0,010425	0,699345	0,298	0,106	1
CORO1B	0,010449	0,424757	0,309	0,128	1
BAZ2B	0,010454	0,747162	0,281	0,106	1
ARPC4	0,01049	0,584899	0,596	0,426	1
MAP3K3	0,010521	0,491596	0,331	0,149	1
RPS6KA3	0,010566	0,301133	0,258	0,085	1
TSG101	0,010566	0,389231	0,258	0,085	1
SP100	0,010596	0,347301	0,41	0,191	1
NAGK	0,010709	0,361859	0,472	0,234	1
RAB2A	0,010741	0,589379	0,449	0,255	1
RNF167	0,010897	0,403836	0,281	0,106	1
TYW3	0,01093	0,454221	0,163	0,021	1
PLXND1	0,01093	0,331762	0,197	0,043	1
CLK3	0,01093	0,331762	0,197	0,043	1
LONP2	0,010931	0,378361	0,197	0,043	1
NUTM2A-A	0,010931	0,53476	0,197	0,043	1
ERCC5	0,010981	0,392595	0,163	0,021	1
MOB4	0,011029	0,314058	0,197	0,043	1
USB1	0,011029	0,36147	0,197	0,043	1
PES1	0,011029	0,36147	0,197	0,043	1
APMAP	0,011029	0,520332	0,197	0,043	1
CUL4A	0,011032	0,32692	0,163	0,021	1
RMDN1	0,011033	0,375352	0,163	0,021	1
CARD9	0,011033	0,375352	0,163	0,021	1
FOXJ3	0,011082	0,308496	0,163	0,021	1
ZNF609	0,011082	0,308496	0,163	0,021	1
CNEP1R1	0,011082	0,308496	0,163	0,021	1
RTN1	0,011083	0,357806	0,163	0,021	1

KIAA0232	0,011083	0,357806	0,163	0,021	1
MBNL2	0,011083	0,404799	0,163	0,021	1
MIOS	0,011126	0,296035	0,197	0,043	1
SF3A3	0,011126	0,296035	0,197	0,043	1
WDR11	0,011126	0,344288	0,197	0,043	1
PPP3R1	0,011126	0,344288	0,197	0,043	1
METAP2	0,01113	0,467491	0,281	0,106	1
DEDD	0,011131	0,289726	0,163	0,021	1
SDAD1	0,011131	0,289726	0,163	0,021	1
SEC24D	0,011131	0,289726	0,163	0,021	1
TFE3	0,011131	0,289726	0,163	0,021	1
PTPRJ	0,011131	0,464945	0,163	0,021	1
MAML3	0,011131	0,339947	0,163	0,021	1
IPO5	0,011131	0,339947	0,163	0,021	1
ZCCHC17	0,011178	0,270596	0,163	0,021	1
IARS2	0,011178	0,270596	0,163	0,021	1
ZZEF1	0,011178	0,270596	0,163	0,021	1
STRN4	0,011178	0,270596	0,163	0,021	1
GABPB1-A!	0,011178	0,321764	0,163	0,021	1
ILKAP	0,011178	0,321764	0,163	0,021	1
RAPGEF1	0,011179	0,27915	0,202	0,043	1
MKLN1	0,011223	0,27768	0,197	0,043	1
U2AF2	0,011223	0,326806	0,197	0,043	1
UROD	0,011223	0,251094	0,163	0,021	1
TBC1D14	0,011223	0,251094	0,163	0,021	1
CDS2	0,011223	0,251094	0,163	0,021	1
BRD7	0,011223	0,303243	0,163	0,021	1
ANTXR2	0,011266	0,416063	0,163	0,021	1
DDX18	0,011323	0,512869	0,455	0,255	1
SCNM1	0,011375	0,630292	0,348	0,17	1
ANXA7	0,011375	0,630292	0,348	0,17	1
SULT1A1	0,011381	1,01216	0,348	0,17	1
HP1BP3	0,011418	0,553865	0,404	0,213	1
SHTN1	0,011429	0,78427	0,309	0,128	1
MTG1	0,011447	0,408016	0,124	0	1
FAM60A	0,011447	0,269982	0,124	0	1
MIER1	0,01146	0,433438	0,253	0,085	1
GID8	0,011461	0,472986	0,253	0,085	1
MLXIP	0,011464	0,464053	0,225	0,064	1
SULT1B1	0,011464	0,289816	0,124	0	1
SCYL2	0,011464	0,289816	0,124	0	1
NR1D2	0,011479	0,255489	0,124	0	1
TNIP2	0,011479	0,255489	0,124	0	1
SLC30A9	0,011479	0,255489	0,124	0	1
CHFR	0,011479	0,255489	0,124	0	1
CDCA4	0,011479	0,255489	0,124	0	1
RP11-24F1	0,011479	0,255489	0,124	0	1
DBNDD2	0,011479	0,255489	0,124	0	1
ZCCHC7	0,011493	0,275609	0,124	0	1
SULT1A2	0,011493	0,275609	0,124	0	1

ZNF276	0,011493	0,275609	0,124	0	1
NACC2	0,011493	0,32834	0,124	0	1
USPL1	0,011493	0,32834	0,124	0	1
LIMK2	0,011505	0,295333	0,124	0	1
RP11-707G	0,011506	0,396243	0,124	0	1
C15orf57	0,011516	0,365436	0,124	0	1
TRMT10C	0,011517	0,413745	0,124	0	1
CHML	0,011525	0,507585	0,124	0	1
ACAA2	0,011532	0,423062	0,258	0,085	1
BLOC1S2	0,011566	0,557735	0,326	0,149	1
NOP10	0,011601	2,047719	0,787	0,617	1
ZNF581	0,011616	0,363464	0,225	0,064	1
IMMT	0,011619	0,449074	0,225	0,064	1
CHSY1	0,011619	0,554797	0,225	0,064	1
SCAMP2	0,011766	0,599838	0,36	0,17	1
COLGALT1	0,011772	0,346887	0,225	0,064	1
ARPC5L	0,011773	0,433867	0,225	0,064	1
SPG21	0,011797	0,525029	0,522	0,34	1
TRPS1	0,011804	0,342713	0,23	0,064	1
HIST1H2AC	0,011909	10,74862	0,191	0,043	1
EFTUD2	0,011927	0,33003	0,225	0,064	1
TNKS2	0,011928	0,48859	0,225	0,064	1
HACD4	0,012015	0,961787	0,371	0,191	1
APP	0,012059	0,504307	0,309	0,128	1
SBF2	0,01207	0,36945	0,281	0,106	1
SNRNP27	0,012082	0,312884	0,225	0,064	1
STX5	0,012082	0,312884	0,225	0,064	1
MAD2L2	0,012216	0,446618	0,23	0,064	1
DENND1A	0,012237	0,295439	0,225	0,064	1
CRYL1	0,012237	0,295439	0,225	0,064	1
HDAC2	0,012237	0,342167	0,225	0,064	1
LRPAP1	0,012241	0,929275	0,404	0,234	1
HSPA6	0,012288	33,73234	0,219	0,064	1
KXD1	0,012357	0,578279	0,309	0,128	1
PRMT2	0,012357	0,508667	0,371	0,191	1
PAFAH1B2	0,012392	0,325229	0,225	0,064	1
HELZ	0,012411	0,325772	0,258	0,085	1
NUP50	0,012467	0,356337	0,253	0,085	1
BAG6	0,012501	0,640707	0,303	0,128	1
TMEM30A	0,012505	0,573794	0,275	0,106	1
GPBP1	0,012608	0,676233	0,416	0,213	1
E2F3	0,012763	0,464187	0,191	0,043	1
RNF19A	0,012763	1,977776	0,191	0,043	1
ADI1	0,012775	-0,27567	0,32	0,128	1
PARVB	0,012817	0,694809	0,219	0,064	1
MED4	0,012892	0,251306	0,258	0,085	1
ARID1A	0,012893	0,618967	0,354	0,17	1
RAB27A	0,012983	0,317428	0,32	0,128	1
CDC42EP2	0,013006	1,316239	0,191	0,043	1
SLC4A7	0,013088	0,2838	0,163	0,021	1

MPPE1	0,013125	0,325286	0,191	0,043	1
CD46	0,013131	0,57696	0,343	0,17	1
FCGR2B	0,013145	0,372955	0,163	0,021	1
DYNLRB1	0,013162	0,429682	0,528	0,319	1
ELMSAN1	0,013178	0,473487	0,225	0,064	1
GPRIN3	0,013185	0,612796	0,219	0,064	1
CEACAM4	0,013191	0,505948	0,157	0,021	1
BCCIP	0,013242	0,457371	0,247	0,085	1
KDM3B	0,013244	0,307465	0,191	0,043	1
UIMC1	0,013244	0,307465	0,191	0,043	1
SAMD4B	0,013245	0,474445	0,191	0,043	1
ZFHX3	0,013245	0,514971	0,191	0,043	1
OGFR	0,013249	0,280582	0,337	0,149	1
ERO1A	0,013255	0,368933	0,157	0,021	1
ESF1	0,013255	0,368933	0,157	0,021	1
BTK	0,013312	0,462364	0,275	0,106	1
FUCA1	0,013315	0,301631	0,157	0,021	1
KPNA3	0,013315	0,301631	0,157	0,021	1
NCOA2	0,013363	0,428483	0,191	0,043	1
EXOC7	0,013363	0,337892	0,191	0,043	1
CBFB	0,013363	0,337892	0,191	0,043	1
SF3A2	0,013363	0,384213	0,191	0,043	1
DPM2	0,013375	0,282731	0,157	0,021	1
MAN2A2	0,013375	0,282731	0,157	0,021	1
CRISPLD2	0,013375	0,282731	0,157	0,021	1
DNAJC13	0,013376	0,333296	0,157	0,021	1
ARFIP1	0,013376	0,333296	0,157	0,021	1
MAPKAPK5	0,013376	0,333296	0,157	0,021	1
MRPS15	0,013394	0,383947	0,309	0,128	1
ZC3H18	0,013434	0,263466	0,157	0,021	1
TMUB1	0,013434	0,263466	0,157	0,021	1
TOR1AIP1	0,01348	0,270843	0,191	0,043	1
CAPN7	0,01348	0,270843	0,191	0,043	1
MAN2C1	0,01348	0,270843	0,191	0,043	1
NFKBID	0,013545	0,855561	0,292	0,128	1
PIN4	0,013585	0,368128	0,275	0,106	1
MAP7D1	0,013585	0,368128	0,275	0,106	1
LSM2	0,013588	0,547869	0,275	0,106	1
CCDC115	0,013596	0,350342	0,191	0,043	1
PIEZO1	0,013596	0,252016	0,191	0,043	1
HTATSF1	0,013596	0,427725	0,191	0,043	1
TBC1D5	0,013735	0,417084	0,219	0,064	1
IGF2BP2	0,01374	0,464832	0,118	0	1
ASRGL1	0,01374	0,282565	0,118	0	1
TFIP11	0,01374	0,282565	0,118	0	1
LRRC47	0,013757	0,302153	0,118	0	1
KBTBD8	0,013757	0,302153	0,118	0	1
SNX19	0,013757	0,302153	0,118	0	1
FASTKD2	0,013771	0,268254	0,118	0	1
TBCC	0,013771	0,268254	0,118	0	1

ATP9B	0,013771	0,268254	0,118	0	1
ZNF431	0,013771	0,268254	0,118	0	1
ERLIN2	0,013772	0,321365	0,118	0	1
EGR3	0,013772	0,890562	0,118	0	1
SLC30A5	0,013784	0,288122	0,118	0	1
WDFY4	0,013784	0,288122	0,118	0	1
WIPI1	0,013784	0,288122	0,118	0	1
DHX34	0,013784	0,288122	0,118	0	1
RIOK1	0,013785	0,389727	0,118	0	1
IL18BP	0,013794	0,307603	0,118	0	1
ENTPD1	0,013798	0,36264	0,281	0,106	1
DERL1	0,013923	0,483931	0,219	0,064	1
ST8SIA4	0,014002	0,499889	0,253	0,085	1
01. Mrz	0,014073	1,037689	0,478	0,319	1
SMIM14	0,014107	0,385423	0,219	0,064	1
TMEM167I	0,014108	0,428214	0,219	0,064	1
LTBR	0,01414	0,505334	0,36	0,17	1
SNRNP70	0,014147	0,336942	0,275	0,106	1
DR1	0,014192	0,438624	0,247	0,085	1
RNF10	0,014205	0,389841	0,253	0,085	1
MSL3	0,014308	0,372816	0,281	0,106	1
MFNG	0,014367	0,348097	0,225	0,064	1
ANAPC15	0,014386	0,382521	0,326	0,149	1
EFR3A	0,014432	0,320976	0,275	0,106	1
COA6	0,014479	0,306502	0,219	0,064	1
AKR1A1	0,014489	0,910333	0,371	0,191	1
MBD2	0,014584	1,243812	0,32	0,149	1
VAMP7	0,014666	0,335969	0,219	0,064	1
CAMKK2	0,014666	0,380882	0,219	0,064	1
SNAP29	0,014673	0,322323	0,247	0,085	1
BRWD1	0,014674	0,366419	0,247	0,085	1
SLC39A1	0,014851	0,271073	0,219	0,064	1
RPP21	0,014852	0,318926	0,219	0,064	1
THOC2	0,014916	0,414349	0,298	0,128	1
MFSD10	0,014917	0,305599	0,247	0,085	1
GOLGB1	0,015148	0,431593	0,185	0,043	1
DNAJC1	0,015162	0,288591	0,247	0,085	1
PSTPIP2	0,015242	0,471407	0,275	0,106	1
ZNRD1	0,015274	0,438489	0,298	0,128	1
NAA10	0,01538	0,329502	0,388	0,191	1
GLO1	0,015408	0,473288	0,247	0,085	1
PDE4A	0,01559	0,382777	0,185	0,043	1
PPIH	0,01559	0,382777	0,185	0,043	1
MBD4	0,01559	0,382777	0,185	0,043	1
MRPS18C	0,015636	0,46206	0,298	0,128	1
MRPS33	0,015641	0,354495	0,197	0,043	1
NFE2	0,015667	0,413977	0,152	0,021	1
VPS26A	0,015701	0,254227	0,191	0,043	1
ARFGEF1	0,015736	0,365961	0,185	0,043	1
KPNA2	0,015736	0,411028	0,185	0,043	1

CCAR1	0,015767	0,721629	0,213	0,064	1
FNIP1	0,015814	0,44358	0,371	0,191	1
ARHGAP31	0,015826	0,331732	0,152	0,021	1
CLASP2	0,01588	0,348857	0,185	0,043	1
DGCR6L	0,01588	0,348857	0,185	0,043	1
RABGAP1L	0,015885	0,530788	0,27	0,106	1
RAD50	0,015905	0,757244	0,152	0,021	1
PTX3	0,015906	0,870564	0,152	0,021	1
DCAF11	0,016022	0,282563	0,185	0,043	1
DLST	0,016022	0,282563	0,185	0,043	1
WDFY1	0,016023	0,331456	0,185	0,043	1
ZMPSTE24	0,016054	0,275687	0,152	0,021	1
NSUN2	0,016054	0,275687	0,152	0,021	1
DBF4	0,016054	0,275687	0,152	0,021	1
PYROXD1	0,016054	0,275687	0,152	0,021	1
TRPM7	0,016054	0,275687	0,152	0,021	1
ZFP91	0,016054	0,326601	0,152	0,021	1
ISG20L2	0,016054	0,326601	0,152	0,021	1
FAM136A	0,016055	0,495984	0,152	0,021	1
MEFV	0,016091	0,396338	0,242	0,085	1
MSL2	0,016126	0,256285	0,152	0,021	1
OARD1	0,016126	0,256285	0,152	0,021	1
XPA	0,016126	0,256285	0,152	0,021	1
BRD3	0,016126	0,256285	0,152	0,021	1
NUTM2B-A	0,016126	0,256285	0,152	0,021	1
STYX	0,016126	0,256285	0,152	0,021	1
ZMAT5	0,016126	0,256285	0,152	0,021	1
SNHG12	0,016126	0,357497	0,152	0,021	1
CUTC	0,016126	0,308171	0,152	0,021	1
DSTN	0,016139	0,633415	0,292	0,128	1
TP53INP2	0,016164	0,263958	0,185	0,043	1
CLASRP	0,016165	0,437753	0,185	0,043	1
SUGP2	0,016165	0,313746	0,185	0,043	1
YIPF6	0,016165	0,313746	0,185	0,043	1
CTDSP1	0,016173	0,460588	0,348	0,17	1
SYNRG	0,016196	0,420466	0,152	0,021	1
PPP6C	0,016227	0,49456	0,371	0,191	1
CDK5	0,016263	0,270259	0,152	0,021	1
SMIM20	0,016305	0,295717	0,185	0,043	1
CALHM2	0,016427	0,411335	0,213	0,064	1
ATIC	0,016435	0,255134	0,112	0	1
RING1	0,016435	0,255134	0,112	0	1
DNAJC25	0,016435	0,255134	0,112	0	1
BCL2L1	0,016435	0,255134	0,112	0	1
PI4K2A	0,016455	0,275261	0,112	0	1
IRF2BPL	0,016455	0,275261	0,112	0	1
MEX3C	0,016473	0,294991	0,112	0	1
ALCAM	0,016473	0,475199	0,112	0	1
NAGPA	0,016473	0,475199	0,112	0	1
DCP1A	0,016488	0,260845	0,112	0	1

RP11-61J1!	0,016488	0,260845	0,112	0	1
GOLIM4	0,016488	0,31434	0,112	0	1
PARP4	0,016497	0,346776	0,219	0,064	1
PNO1	0,016501	0,280859	0,112	0	1
PLCB1	0,016501	0,280859	0,112	0	1
LPAR6	0,016503	0,894337	0,27	0,106	1
S1PR3	0,016513	0,351949	0,112	0	1
BCL2	0,016522	0,370236	0,112	0	1
C1QA	0,016525	20,81822	0,112	0	1
DHX15	0,016541	0,388649	0,27	0,106	1
STAU1	0,016542	0,42872	0,27	0,106	1
CCL5	0,016564	40,81822	0,112	0	1
INTS10	0,01665	0,395539	0,213	0,064	1
USP47	0,016657	0,36505	0,242	0,085	1
BCL7B	0,016661	0,550545	0,242	0,085	1
POLR2K	0,016687	0,465476	0,32	0,149	1
NASP	0,016871	0,334512	0,213	0,064	1
NSD1	0,016873	0,379489	0,213	0,064	1
DNASE2	0,016873	0,379489	0,213	0,064	1
SEMA4D	0,016874	0,422529	0,213	0,064	1
C3AR1	0,016874	0,422529	0,213	0,064	1
TMEM205	0,016927	0,677173	0,494	0,319	1
FUBP1	0,016946	0,572974	0,242	0,085	1
IFI35	0,017047	0,384715	0,326	0,149	1
CISD3	0,017096	0,40691	0,213	0,064	1
POLR2L	0,017168	4,808636	0,837	0,702	1
TAF11	0,01723	0,332752	0,242	0,085	1
COPB1	0,01723	0,332752	0,242	0,085	1
UBXN2B	0,017231	0,486236	0,242	0,085	1
DECR1	0,017233	0,970234	0,416	0,255	1
ZNF24	0,01731	0,326914	0,219	0,064	1
TTC3	0,017339	1,44752	0,36	0,191	1
SPG11	0,017395	0,313491	0,275	0,106	1
PAPSS2	0,017431	4,781782	0,18	0,043	1
STAG2	0,017454	0,462638	0,365	0,191	1
GOLT1B	0,017519	0,360564	0,242	0,085	1
ACO2	0,017533	0,311139	0,219	0,064	1
CEBPZ	0,017552	0,540503	0,236	0,085	1
AAMP	0,017754	0,409105	0,292	0,128	1
DAP	0,017765	0,312582	0,213	0,064	1
LPGAT1	0,017769	0,675954	0,264	0,106	1
NAA50	0,017769	1,240932	0,264	0,106	1
CHCHD10	0,017892	1,348009	0,489	0,319	1
C11orf58	0,01799	0,915438	0,472	0,298	1
FAM214B	0,018305	0,364499	0,18	0,043	1
LZIC	0,018411	0,310749	0,185	0,043	1
TMF1	0,018604	0,41932	0,292	0,128	1
PAK1	0,018714	0,590519	0,371	0,191	1
SFXN3	0,018834	0,312205	0,18	0,043	1
TIMMDC1	0,018835	0,359703	0,18	0,043	1

ARRB1	0,018836	0,405046	0,18	0,043	1
AVPI1	0,018876	0,479843	0,146	0,021	1
GK5	0,018876	0,479843	0,146	0,021	1
KMT2E-AS:	0,018907	0,452031	0,264	0,106	1
TGFBR2	0,018909	0,627555	0,264	0,106	1
ADAM9	0,01893	0,260291	0,185	0,043	1
WDFY3	0,019008	0,294148	0,18	0,043	1
SDHA	0,019008	0,294148	0,18	0,043	1
HERPUD2	0,019008	0,294148	0,18	0,043	1
ARHGAP24	0,019009	0,463187	0,18	0,043	1
GNPAT	0,019009	0,342491	0,18	0,043	1
MRPS34	0,019015	0,440462	0,32	0,149	1
P4HA1	0,019064	0,752867	0,146	0,021	1
APAF1	0,019087	0,516399	0,208	0,064	1
ITPR1	0,019156	0,28776	0,146	0,021	1
DHX38	0,019156	0,28776	0,146	0,021	1
TBC1D23	0,019181	0,275759	0,18	0,043	1
PSPC1	0,019181	0,275759	0,18	0,043	1
ATXN3	0,019181	0,275759	0,18	0,043	1
LMF2	0,019182	0,324977	0,18	0,043	1
STX8	0,019182	0,471507	0,236	0,085	1
PCID2	0,019247	0,268593	0,146	0,021	1
EXOC1	0,019247	0,268593	0,146	0,021	1
YKT6	0,019247	0,31986	0,146	0,021	1
TMEM170/	0,019247	0,31986	0,146	0,021	1
CAB39	0,019292	0,356768	0,264	0,106	1
EPS15	0,019303	0,911793	0,315	0,149	1
SECISBP2L	0,019335	0,474671	0,146	0,021	1
COA5	0,019342	0,378094	0,208	0,064	1
PLEKHA2	0,019353	0,257025	0,18	0,043	1
CEP57	0,019353	0,257025	0,18	0,043	1
TRPV2	0,019353	0,257025	0,18	0,043	1
CLEC10A	0,019448	11,71545	0,202	0,064	1
NSMAF	0,019523	0,289002	0,18	0,043	1
SLC38A10	0,019608	0,361759	0,208	0,064	1
CCDC186	0,019611	0,405553	0,208	0,064	1
GPR84	0,019692	1,24151	0,107	0	1
PHF1	0,019692	0,267904	0,107	0	1
OBFC1	0,019692	0,267904	0,107	0	1
LRRC8D	0,019711	0,287778	0,107	0	1
SLC46A2	0,019711	0,287778	0,107	0	1
MRPS31	0,019711	0,287778	0,107	0	1
TOR3A	0,019726	0,25338	0,107	0	1
REV1	0,019726	0,25338	0,107	0	1
FAM35A	0,019726	0,25338	0,107	0	1
SETDB1	0,019727	0,307265	0,107	0	1
IPO9	0,019727	0,307265	0,107	0	1
ACVR1B	0,019727	0,307265	0,107	0	1
FAM101B	0,019727	0,307265	0,107	0	1
CYP51A1	0,019727	0,766385	0,107	0	1

SRFBP1	0,019754	0,441372	0,107	0	1
FPR3	0,01977	0,429176	0,107	0	1
CST7	0,019792	8,874578	0,107	0	1
N4BP2L2	0,019832	0,338379	0,517	0,319	1
MAPKAPK2	0,019851	0,442518	0,236	0,085	1
NFIC	0,019851	0,442518	0,236	0,085	1
CCNG1	0,019874	0,345153	0,208	0,064	1
GYG1	0,019888	0,694885	0,275	0,106	1
TMBIM4	0,019958	1,106439	0,528	0,34	1
PSTPIP1	0,019999	0,569477	0,382	0,213	1
UBE2G2	0,020142	0,373519	0,208	0,064	1
GIMAP4	0,020295	1,300855	0,483	0,298	1
SMARCC1	0,020407	0,31109	0,208	0,064	1
PIK3CD	0,020407	0,31109	0,208	0,064	1
DOCK10	0,020409	0,511743	0,208	0,064	1
NCOA3	0,020483	0,309064	0,264	0,106	1
UHRF1BP1	0,020515	0,281905	0,242	0,085	1
PPIP5K2	0,020675	0,497467	0,208	0,064	1
PARP1	0,020941	0,275826	0,208	0,064	1
DNPEP	0,020941	0,275826	0,208	0,064	1
RBM22	0,020941	0,323458	0,208	0,064	1
SARS	0,021082	0,514644	0,287	0,128	1
SLC6A6	0,021111	0,488515	0,258	0,106	1
A1BG	0,021111	0,584347	0,258	0,106	1
ZFAND1	0,021208	0,257717	0,208	0,064	1
DHPS	0,021208	0,306198	0,208	0,064	1
PCBD1	0,021216	0,293113	0,236	0,085	1
DESI1	0,021216	0,338487	0,236	0,085	1
GLUD1	0,021486	0,794299	0,337	0,17	1
SP110	0,021597	0,511099	0,292	0,128	1
PTPN12	0,021623	0,318665	0,298	0,128	1
ARHGAP18	0,021674	0,995678	0,174	0,043	1
MALT1	0,021821	1,073132	0,303	0,149	1
P2RX4	0,021912	0,422776	0,236	0,085	1
TMEM9B	0,021914	0,587319	0,315	0,149	1
DNAJA4	0,022123	2,93985	0,14	0,021	1
ITGAE	0,022167	0,468274	0,281	0,128	1
AP3S1	0,022185	0,732473	0,399	0,234	1
TIMM8B	0,022228	0,522059	0,466	0,277	1
AFTPH	0,022273	0,446218	0,309	0,149	1
CLIP4	0,022301	0,487991	0,174	0,043	1
XRN1	0,022449	0,366536	0,258	0,106	1
NAP1L4	0,022511	0,353405	0,174	0,043	1
WDR74	0,022512	0,442661	0,174	0,043	1
KCNE1	0,022594	0,384501	0,14	0,021	1
CD1C	0,0226	6,809316	0,14	0,021	1
UBE2G1	0,022719	0,287422	0,174	0,043	1
YY1AP1	0,022719	0,287422	0,174	0,043	1
NUDT4	0,02272	0,336083	0,174	0,043	1
POP4	0,02272	0,336083	0,174	0,043	1

POU2F2	0,022746	1,97253	0,461	0,298	1
COX20	0,022813	0,651707	0,292	0,128	1
PSMD2	0,022813	0,504534	0,23	0,085	1
GMPS	0,022927	0,268908	0,174	0,043	1
TCEAL4	0,022927	0,441915	0,174	0,043	1
NADSYN1	0,022927	0,318456	0,174	0,043	1
PTOV1	0,022927	0,318456	0,174	0,043	1
FLVCR2	0,02294	0,331415	0,14	0,021	1
P2RX1	0,02294	0,331415	0,14	0,021	1
SMARCD3	0,022954	-0,27021	0,18	0,043	1
KLF3	0,023045	0,653424	0,309	0,149	1
VPS13D	0,02305	0,261448	0,14	0,021	1
YIPF5	0,02305	0,261448	0,14	0,021	1
MRPL32	0,02305	0,261448	0,14	0,021	1
NLRC5	0,02305	0,261448	0,14	0,021	1
TMEM199	0,02305	0,261448	0,14	0,021	1
SLC35C2	0,02305	0,261448	0,14	0,021	1
ABHD17A	0,02305	0,261448	0,14	0,021	1
CERK	0,02305	0,261448	0,14	0,021	1
ACSS2	0,02305	0,441266	0,14	0,021	1
UFL1	0,02305	0,313074	0,14	0,021	1
CUL1	0,02305	0,313074	0,14	0,021	1
PTRH2	0,02305	0,313074	0,14	0,021	1
SATB1	0,023056	0,457018	0,202	0,064	1
SOD1	0,023065	0,986115	0,494	0,298	1
NAPRT	0,023111	0,385242	0,287	0,128	1
TIPRL	0,023133	0,250044	0,174	0,043	1
BBIP1	0,023133	0,250044	0,174	0,043	1
ERN1	0,023133	0,250044	0,174	0,043	1
PTGES2	0,023133	0,300512	0,174	0,043	1
PIK3C2A	0,023133	0,300512	0,174	0,043	1
BEX4	0,023158	0,711881	0,14	0,021	1
COG5	0,023158	0,424849	0,14	0,021	1
RCC2	0,023158	0,29439	0,14	0,021	1
GATAD1	0,023158	0,29439	0,14	0,021	1
BORCS5	0,023158	0,29439	0,14	0,021	1
PPP6R2	0,023158	0,29439	0,14	0,021	1
TLR8	0,023264	0,699381	0,14	0,021	1
UBA6	0,023264	0,275351	0,14	0,021	1
AP2A2	0,023264	0,275351	0,14	0,021	1
DCAF10	0,023367	0,255942	0,14	0,021	1
PHPT1	0,023371	0,693891	0,522	0,362	1
DNAJC7	0,02345	0,470047	0,388	0,213	1
OASL	0,02355	0,399755	0,101	0	1
IKZF5	0,02355	0,260492	0,101	0	1
POLR3E	0,02355	0,260492	0,101	0	1
RP11-362F	0,02355	0,260492	0,101	0	1
FOXO1	0,02357	0,417196	0,101	0	1
SSBP3	0,02357	0,332993	0,101	0	1
PRPF19	0,02357	0,280513	0,101	0	1

DIP2A	0,023587	0,351626	0,101	0	1
PIM2	0,023587	0,479501	0,101	0	1
SLC25A25	0,023599	0,266173	0,101	0	1
CLPX	0,023599	0,266173	0,101	0	1
PDXDC1	0,023599	0,266173	0,101	0	1
SAE1	0,023599	0,266173	0,101	0	1
RALB	0,023603	0,437093	0,23	0,085	1
ID3	0,023635	10,81826	0,101	0	1
CYP27A1	0,023686	1,952544	0,202	0,064	1
STOML2	0,023998	0,321982	0,202	0,064	1
GOLPH3	0,024001	0,380543	0,23	0,085	1
ABHD2	0,024004	0,462183	0,23	0,085	1
RPL31	0,024042	3,807371	0,966	0,936	1
MRPL41	0,024163	0,552055	0,376	0,213	1
MRPL34	0,024312	0,515323	0,303	0,149	1
C22orf39	0,024315	0,304696	0,202	0,064	1
H2AFV	0,024351	0,664697	0,522	0,362	1
ARRDC1	0,024385	0,412875	0,281	0,128	1
DNAJB12	0,024388	0,450854	0,281	0,128	1
ZFAND2B	0,024403	0,320603	0,23	0,085	1
IPMK	0,024406	0,407074	0,23	0,085	1
CAPRIN1	0,024515	0,624948	0,253	0,106	1
SYPL1	0,024553	0,303138	0,236	0,085	1
CNOT4	0,024633	0,287107	0,202	0,064	1
ARID4A	0,024635	0,379206	0,202	0,064	1
PDCD10	0,024795	0,33082	0,258	0,106	1
TM2D1	0,024809	0,30385	0,23	0,085	1
TAF10	0,024955	0,474852	0,208	0,064	1
CDC26	0,024961	0,398532	0,281	0,128	1
RCBTB2	0,024972	0,309155	0,27	0,106	1
RPL21	0,025198	43,61795	1	1	1
UBE2W	0,02527	0,250971	0,202	0,064	1
SIGLEC9	0,025545	0,469721	0,253	0,106	1
RPAIN	0,02563	0,269478	0,23	0,085	1
USP48	0,02563	0,315911	0,23	0,085	1
FAM200B	0,025707	0,352277	0,438	0,255	1
PTPN7	0,025708	0,315074	0,174	0,043	1
NSRP1	0,026075	0,588671	0,253	0,106	1
TRIM25	0,026111	0,483171	0,169	0,043	1
RAB18	0,026138	0,369213	0,281	0,128	1
EIF3J	0,026253	0,426396	0,287	0,128	1
NDUFA4	0,026303	2,907854	0,775	0,617	1
RNF141	0,026434	0,443911	0,298	0,149	1
RAB32	0,026479	0,716929	0,41	0,255	1
CCT8	0,026479	0,716929	0,41	0,255	1
LILRA6	0,026546	0,316151	0,258	0,106	1
SNX29	0,026613	0,364202	0,169	0,043	1
LBR	0,026637	0,387286	0,287	0,128	1
MDM4	0,026724	0,346118	0,292	0,128	1
GPAA1	0,026862	0,298951	0,169	0,043	1

SEC16A	0,026862	0,298951	0,169	0,043	1
NAF1	0,027027	1,094679	0,135	0,021	1
KPNA6	0,027113	0,280651	0,169	0,043	1
PSMG4	0,027113	0,280651	0,169	0,043	1
INPPL1	0,027113	0,280651	0,169	0,043	1
PDIA4	0,027189	0,411141	0,202	0,064	1
ARHGAP21	0,027305	0,342837	0,135	0,021	1
ENG	0,027306	0,858797	0,135	0,021	1
KIAA0319L	0,027362	0,26201	0,169	0,043	1
ACBD6	0,027362	0,26201	0,169	0,043	1
QRICH1	0,027362	0,26201	0,169	0,043	1
ESYT2	0,027362	0,26201	0,169	0,043	1
API5	0,027363	0,311892	0,169	0,043	1
CBX6	0,027441	0,273693	0,135	0,021	1
OAS2	0,027442	0,451507	0,135	0,021	1
RAD51C	0,027443	0,373243	0,135	0,021	1
ZNF326	0,027576	0,254252	0,135	0,021	1
WASL	0,027576	0,254252	0,135	0,021	1
OXLD1	0,027576	0,254252	0,135	0,021	1
PPP3CC	0,027576	0,306241	0,135	0,021	1
WDR43	0,027576	0,306241	0,135	0,021	1
PSMB8-AS:	0,027687	0,37191	0,253	0,106	1
F5	0,027708	0,418741	0,135	0,021	1
PPP6R1	0,027708	0,287428	0,135	0,021	1
CALM3	0,027711	0,974807	0,528	0,362	1
PHB	0,027772	0,900156	0,281	0,128	1
COPZ1	0,027793	0,82692	0,331	0,17	1
TMEM131	0,027837	0,319539	0,135	0,021	1
DCPS	0,027837	0,268254	0,135	0,021	1
CCSER2	0,027837	0,268254	0,135	0,021	1
DSC2	0,027837	0,268254	0,135	0,021	1
MLLT1	0,027837	0,268254	0,135	0,021	1
KIAA2026	0,027837	0,268254	0,135	0,021	1
MRPS23	0,027856	0,275434	0,169	0,043	1
POLR2J	0,027918	0,310516	0,399	0,234	1
NUCB1	0,027995	1,562585	0,455	0,277	1
CALU	0,0281	0,256695	0,169	0,043	1
SETD5	0,028144	0,286096	0,23	0,085	1
CYB5R3	0,028154	0,495764	0,275	0,128	1
FNDC3B	0,028386	0,618061	0,258	0,106	1
RAB8B	0,028405	0,382832	0,337	0,17	1
SMIM15	0,028474	0,37477	0,225	0,085	1
BBX	0,028476	0,522383	0,225	0,085	1
AP3B1	0,028476	0,522383	0,225	0,085	1
ATP6V1C1	0,028477	0,456864	0,225	0,085	1
TPPP3	0,028609	-0,71836	0,067	0,17	1
TTYH3	0,028634	0,403347	0,258	0,106	1
MYCBP	0,028921	0,539458	0,197	0,064	1
AGO4	0,02895	0,358908	0,225	0,085	1
SMC1A	0,028998	0,528308	0,247	0,106	1

RNASE2	0,029157	0,606366	0,281	0,128	1
RNF44	0,029429	0,34279	0,225	0,085	1
CMTM3	0,02949	0,469199	0,275	0,128	1
RAB10	0,029534	1,272218	0,449	0,319	1
PADI4	0,029608	0,931861	0,163	0,043	1
C12orf10	0,029673	0,262534	0,197	0,064	1
PPIG	0,029701	0,445001	0,438	0,277	1
COA3	0,029785	0,362934	0,331	0,17	1
MAP3K1	0,029809	0,381193	0,382	0,213	1
CREBRF	0,029911	0,28047	0,225	0,085	1
CMC1	0,029913	10,80869	0,225	0,085	1
IRAK4	0,030052	0,340131	0,197	0,064	1
PSENEN	0,030288	0,45862	0,354	0,191	1
MRPL13	0,030397	0,263025	0,225	0,085	1
RAE1	0,030431	0,275513	0,197	0,064	1
NME1	0,030888	0,769388	0,169	0,043	1
ARHGAP27	0,031449	0,537044	0,247	0,106	1
FCGR1B	0,031514	0,470025	0,219	0,085	1
ALG3	0,03171	0,476886	0,163	0,043	1
LSM10	0,031742	0,527237	0,348	0,191	1
MX1	0,031776	2,557341	0,197	0,064	1
PSMD7	0,031782	0,67957	0,393	0,234	1
WAPL	0,031783	0,44047	0,32	0,17	1
FAM198B	0,031812	0,561187	0,253	0,106	1
CHST11	0,03201	0,461591	0,163	0,043	1
MOSPD2	0,03201	0,34069	0,163	0,043	1
CFDP1	0,032079	0,381531	0,247	0,106	1
PLXNB2	0,032269	0,290864	0,281	0,128	1
CLCN3	0,032308	0,273834	0,163	0,043	1
CREBL2	0,032308	0,273834	0,163	0,043	1
EPB41L3	0,032308	0,711894	0,225	0,085	1
EIF2AK4	0,032309	0,323144	0,163	0,043	1
NDUFS8	0,032475	0,74804	0,506	0,362	1
PUM3	0,032606	0,255063	0,163	0,043	1
ALDH3A2	0,032606	0,255063	0,163	0,043	1
BCAT1	0,032607	0,70746	0,163	0,043	1
TAF13	0,032607	0,305285	0,163	0,043	1
CHD8	0,032628	0,28579	0,129	0,021	1
POLDIP3	0,032628	0,28579	0,129	0,021	1
PRKACB	0,03263	0,336205	0,129	0,021	1
MMGT1	0,032716	0,323558	0,247	0,106	1
PPIL2	0,032795	0,266585	0,129	0,021	1
HMGCS1	0,032797	1,238944	0,129	0,021	1
TIA1	0,032797	0,317953	0,129	0,021	1
PLEKHM2	0,032797	0,36681	0,129	0,021	1
MFSD11	0,03312	0,280417	0,129	0,021	1
NFKBIE	0,033197	0,268581	0,163	0,043	1
TRAF1	0,033278	0,261107	0,129	0,021	1
PIAS2	0,033278	0,261107	0,129	0,021	1
ERC1	0,033278	0,261107	0,129	0,021	1

DCTN5	0,033278	0,261107	0,129	0,021	1
PTS	0,033288	0,29204	0,197	0,064	1
PDCL3	0,033437	0,3265	0,191	0,064	1
DENND4A	0,03344	0,371831	0,191	0,064	1
IMPDH2	0,033555	0,30724	0,225	0,085	1
FKBP3	0,033555	0,30724	0,225	0,085	1
PIP4K2A	0,033708	0,368964	0,219	0,085	1
LY6E	0,033836	2,842513	0,528	0,362	1
ADAMTSL4	0,033852	0,316965	0,202	0,064	1
SEMA4A	0,033886	0,548136	0,191	0,064	1
PLEKHJ1	0,03402	0,377255	0,247	0,106	1
BABAM1	0,034266	0,308304	0,219	0,085	1
HAGH	0,034327	0,291785	0,191	0,064	1
CAMK2D	0,03433	0,495976	0,191	0,064	1
YWHAH	0,034482	2,246236	0,343	0,191	1
ATF6	0,034746	0,426769	0,27	0,128	1
PIGBOS1	0,034774	0,273965	0,191	0,064	1
SPOP	0,035222	0,255821	0,191	0,064	1
IKBIP	0,035241	0,259644	0,225	0,085	1
IL1R2	0,035248	4,014528	0,157	0,043	1
TESC	0,035347	0,257194	0,247	0,106	1
SCFD1	0,035401	0,320312	0,219	0,085	1
TRPC4AP	0,035401	0,320312	0,219	0,085	1
ZNHIT3	0,035972	0,25653	0,219	0,085	1
C4orf48	0,035986	1,018188	0,612	0,489	1
MIR4435-2	0,036033	0,969775	0,365	0,213	1
CDC42SE2	0,036036	0,504358	0,343	0,191	1
RRBP1	0,03612	0,641643	0,27	0,128	1
CD151	0,036149	0,314853	0,275	0,128	1
FIBP	0,036168	0,473738	0,343	0,191	1
ANXA4	0,036351	0,358208	0,27	0,128	1
TSEN34	0,036471	0,748284	0,292	0,149	1
MCTS1	0,036474	0,559907	0,292	0,149	1
FRG1	0,036822	0,339503	0,275	0,128	1
LDHB	0,036882	4,89734	0,556	0,447	1
C19orf53	0,037	1,218509	0,612	0,511	1
CES1	0,037086	0,668618	0,129	0,021	1
GRHPR	0,037222	0,426231	0,32	0,17	1
NUCB2	0,037375	0,413628	0,157	0,043	1
CACNA2D3	0,03773	0,738691	0,157	0,043	1
DNAJC10	0,037732	0,397327	0,157	0,043	1
RBM23	0,037826	0,482605	0,242	0,106	1
BECN1	0,03783	0,455508	0,242	0,106	1
DPP8	0,038085	0,285519	0,157	0,043	1
MRPL3	0,03814	0,499222	0,264	0,128	1
CTBP2	0,038442	0,266969	0,157	0,043	1
CARD8-AS1	0,038442	0,266969	0,157	0,043	1
STXBP3	0,038444	0,316611	0,157	0,043	1
NDUFAF2	0,038444	0,316611	0,157	0,043	1
UBAC1	0,038444	0,363905	0,157	0,043	1

STX7	0,038494	0,629771	0,331	0,191	1
MARCO	0,038499	1,208758	0,275	0,128	1
RAB4A	0,038575	0,401781	0,242	0,106	1
UBE2V2	0,038958	0,274384	0,219	0,085	1
RPRD1A	0,038958	0,278768	0,124	0,021	1
SIDT2	0,039023	0,769493	0,264	0,128	1
ATAD2B	0,039152	0,560411	0,157	0,043	1
AKAP17A	0,039152	0,280328	0,157	0,043	1
LRMP	0,039152	0,280328	0,157	0,043	1
HES4	0,039163	0,360336	0,124	0,021	1
VPS25	0,039168	0,37892	0,213	0,085	1
RUFY1	0,039359	0,292434	0,124	0,021	1
SYS1	0,039554	0,406412	0,124	0,021	1
MRTO4	0,039554	0,273357	0,124	0,021	1
SLC8B1	0,039554	0,273357	0,124	0,021	1
FAM102B	0,039554	0,273357	0,124	0,021	1
KLHL15	0,039554	0,273357	0,124	0,021	1
WDR46	0,039745	0,253909	0,124	0,021	1
SLC25A19	0,039745	0,253909	0,124	0,021	1
CCNL2	0,039745	0,253909	0,124	0,021	1
TMEM101	0,039745	0,253909	0,124	0,021	1
RANBP3	0,039745	0,253909	0,124	0,021	1
PELI2	0,039933	0,37211	0,124	0,021	1
CHD4	0,040095	0,371642	0,242	0,106	1
RNASEH2B	0,040149	0,349277	0,185	0,064	1
KRCC1	0,040151	0,757063	0,185	0,064	1
FAM173A	0,040475	0,302097	0,213	0,085	1
MAP3K13	0,040477	0,347074	0,213	0,085	1
VBP1	0,040673	0,285266	0,185	0,064	1
TMEM87A	0,040673	0,285266	0,185	0,064	1
KDM2A	0,040676	0,377527	0,185	0,064	1
PPHLN1	0,040866	0,426281	0,242	0,106	1
BMP2K	0,0412	0,267328	0,185	0,064	1
CYB5A	0,041708	-1,34329	0,09	0,191	1
CWC25	0,042437	0,280094	0,242	0,106	1
USE1	0,042478	0,297318	0,213	0,085	1
SEC11C	0,042708	0,392949	0,264	0,128	1
MLKL	0,043263	0,37642	0,124	0,021	1
HNRNPH2	0,043898	0,514843	0,326	0,17	1
USP14	0,043982	0,423797	0,152	0,043	1
TNRC6A	0,044404	0,36244	0,152	0,043	1
FAAP20	0,044637	0,548238	0,354	0,213	1
ARHGEF3	0,044826	0,29707	0,152	0,043	1
OSBPL8	0,045113	1,387425	0,517	0,404	1
ZNF148	0,045251	0,278735	0,152	0,043	1
SS18L2	0,04529	0,630698	0,348	0,213	1
BCL10	0,045373	0,536817	0,236	0,106	1
DEF6	0,045415	0,345679	0,208	0,085	1
RAB21	0,045425	0,430096	0,208	0,085	1
NT5C2	0,045653	0,475513	0,18	0,064	1

CCDC50	0,045675	0,260058	0,152	0,043	1
MGST1	0,04604	0,463153	0,449	0,277	1
TOMM40	0,046098	0,698553	0,152	0,043	1
ELK3	0,046098	0,291939	0,152	0,043	1
MCFD2	0,046554	0,307838	0,185	0,064	1
DIS3	0,046723	0,252215	0,118	0,021	1
RIN2	0,046725	0,304308	0,118	0,021	1
MOAP1	0,046725	0,304308	0,118	0,021	1
G6PC3	0,046938	0,385804	0,152	0,043	1
NNT	0,046964	0,335889	0,118	0,021	1
CHCHD1	0,047141	0,435395	0,236	0,106	1
FXYD6	0,047198	0,266246	0,118	0,021	1
PCTP	0,047198	0,266246	0,118	0,021	1
ENTPD6	0,047199	0,727251	0,118	0,021	1
PDE4D	0,04743	0,299033	0,118	0,021	1
RCHY1	0,047658	0,667799	0,118	0,021	1
CAT	0,048083	0,468126	0,354	0,213	1
KLF11	0,048102	0,278704	0,18	0,064	1
MRPS25	0,048102	0,278704	0,18	0,064	1
POGZ	0,048104	0,326202	0,18	0,064	1
GPATCH8	0,048106	0,371546	0,18	0,064	1
AHCY	0,048106	0,371546	0,18	0,064	1
TCN2	0,048481	0,278676	0,208	0,085	1
PDLIM2	0,048483	0,324694	0,208	0,085	1
CYP20A1	0,048723	0,260647	0,18	0,064	1
COMMD2	0,048723	0,260647	0,18	0,064	1
S1PR4	0,048723	0,260647	0,18	0,064	1
PHYKPL	0,048725	0,547897	0,18	0,064	1
SUGT1	0,04876	0,485305	0,236	0,106	1
KIAA0930	0,048951	0,290636	0,236	0,106	1
EIF3B	0,049109	0,387183	0,213	0,085	1
CDC73	0,049263	0,30801	0,208	0,085	1
MBOAT7	0,049623	0,750581	0,393	0,255	1
GPAT3	0,049662	1,528945	0,23	0,106	1
CASP8	0,04997	0,27365	0,18	0,064	1
RASSF3	0,050041	0,304858	0,258	0,128	1
TRADD	0,050049	0,291043	0,208	0,085	1
HIST1H1C	0,050277	3,775693	0,281	0,149	1
UBE2D1	0,050703	0,96866	0,5	0,404	1
HPS3	0,051153	0,372877	0,258	0,128	1
POLE3	0,051223	0,37996	0,23	0,106	1
ACAA1	0,051299	0,554284	0,326	0,191	1
SUMO3	0,051458	1,780922	0,343	0,213	1
MLEC	0,051486	0,383685	0,236	0,106	1
HSPB11	0,052275	0,357945	0,258	0,128	1
DOCK5	0,052545	0,354438	0,281	0,149	1
LDLRAD4	0,052558	0,550001	0,18	0,064	1
CNOT6L	0,052569	0,39854	0,202	0,085	1
ASAP1	0,053236	0,349146	0,23	0,106	1
EMC10	0,053236	0,349146	0,23	0,106	1

PLEKHO2	0,053238	0,390799	0,23	0,106	1
RHOT1	0,053411	0,256093	0,258	0,128	1
PTPMT1	0,05369	0,271905	0,146	0,043	1
MSRB1	0,054079	0,382105	0,275	0,149	1
JKAMP	0,054192	0,253098	0,146	0,043	1
ARRDC4	0,054192	0,253098	0,146	0,043	1
PAFAH1B3	0,054194	0,303416	0,146	0,043	1
MTHFD2	0,054212	0,655608	0,343	0,213	1
FCGR3A	0,054603	2,588983	0,197	0,085	1
SAMD9L	0,054627	1,306162	0,174	0,064	1
TM6SF1	0,054694	0,810483	0,146	0,043	1
ATP6V1A	0,054768	0,425313	0,253	0,128	1
IFI44	0,054931	0,257353	0,18	0,064	1
DDT	0,055256	0,743208	0,539	0,447	1
CSF2RB	0,055338	0,469389	0,174	0,064	1
C6orf48	0,055402	0,367588	0,365	0,213	1
HMGCR	0,055708	0,297414	0,112	0,021	1
PIH1D1	0,055989	0,3716	0,253	0,128	1
DNAJB4	0,055995	0,278433	0,112	0,021	1
TCEAL3	0,055995	0,278433	0,112	0,021	1
MIF4GD	0,056057	0,289953	0,174	0,064	1
FRMD4B	0,056112	0,449311	0,202	0,085	1
TMEM147	0,056186	0,310365	0,399	0,255	1
ASGR1	0,056213	-0,27977	0,539	0,383	1
PRPF6	0,056338	0,255414	0,23	0,106	1
MILR1	0,056786	0,439366	0,174	0,064	1
PHF5A	0,057012	0,272282	0,202	0,085	1
C1orf123	0,057512	0,253922	0,174	0,064	1
TRIAP1	0,057513	0,694664	0,174	0,064	1
SPTSSA	0,058181	0,362124	0,258	0,128	1
ISCA1	0,058243	0,284955	0,174	0,064	1
TMEM91	0,058243	0,284955	0,174	0,064	1
MEF2C	0,058743	2,713532	0,539	0,383	1
ITGB2-AS1	0,058855	0,56576	0,225	0,106	1
DNAJC2	0,058946	0,420031	0,281	0,149	1
NRG1	0,059275	1,899456	0,36	0,234	1
EHBP1L1	0,05977	0,282918	0,253	0,128	1
MRPS6	0,059776	0,38221	0,343	0,213	1
TNFSF10	0,059924	0,688613	0,315	0,17	1
PFKL	0,059971	0,381749	0,281	0,149	1
SMAD2	0,061152	0,504472	0,225	0,106	1
MVP	0,061206	0,253404	0,258	0,128	1
IMP4	0,061737	0,39287	0,197	0,085	1
GARS	0,062315	0,299653	0,225	0,106	1
USP9X	0,062322	0,385263	0,225	0,106	1
GBP1	0,062592	3,460409	0,202	0,085	1
RHOC	0,062639	1,38969	0,309	0,191	1
DAZAP1	0,063778	0,361472	0,197	0,085	1
ZBTB8OS	0,064042	0,262648	0,303	0,17	1
ZKSCAN1	0,064117	0,296031	0,174	0,064	1

CCDC90B	0,064405	0,251161	0,174	0,064	1
SPTAN1	0,064801	0,327502	0,14	0,043	1
BROX	0,064811	0,345396	0,197	0,085	1
PTPN11	0,065391	0,309722	0,14	0,043	1
NEK7	0,065391	0,259728	0,14	0,043	1
VAV1	0,065391	0,259728	0,14	0,043	1
CIITA	0,065754	0,263215	0,174	0,064	1
DTX3L	0,065852	0,329056	0,197	0,085	1
CIR1	0,066198	0,863518	0,27	0,149	1
NDUFAF4	0,066375	0,290473	0,107	0,021	1
NRGN	0,066596	0,29325	0,253	0,128	1
GBP5	0,06672	0,271359	0,107	0,021	1
CUL5	0,066899	0,265847	0,197	0,085	1
ANKRD17	0,066899	0,265847	0,197	0,085	1
SLC37A2	0,067061	0,303981	0,107	0,021	1
TATDN3	0,067061	0,251871	0,107	0,021	1
KDM4C	0,067061	0,251871	0,107	0,021	1
NOTCH1	0,067061	0,251871	0,107	0,021	1
URI1	0,067061	0,251871	0,107	0,021	1
CD82	0,067061	0,718209	0,107	0,021	1
MRPL42	0,067061	0,433272	0,107	0,021	1
CYP1B1	0,06718	0,652099	0,253	0,128	1
DENND2D	0,067398	0,671224	0,107	0,021	1
CYSLTR1	0,067398	0,3703	0,107	0,021	1
NUP153	0,067761	0,296134	0,169	0,064	1
ATF7	0,067958	0,295554	0,197	0,085	1
E2F4	0,067958	0,295554	0,197	0,085	1
PA2G4	0,068324	0,848318	0,393	0,277	1
EAF2	0,068615	0,278392	0,169	0,064	1
CCDC88B	0,068615	0,278392	0,169	0,064	1
ARMC8	0,068615	0,278392	0,169	0,064	1
SPECC1	0,06873	0,339451	0,14	0,043	1
NDUFA5	0,069371	0,395334	0,298	0,17	1
NDUFS4	0,069433	0,368976	0,287	0,149	1
SCARB2	0,069472	0,666311	0,169	0,064	1
INSR	0,069472	0,308686	0,169	0,064	1
COA1	0,069472	0,260329	0,169	0,064	1
SLIRP	0,069955	0,916579	0,517	0,404	1
GGA1	0,070064	0,334324	0,225	0,106	1
NEDD8	0,070092	-0,82509	0,691	0,574	1
MGST2	0,070186	0,250481	0,348	0,191	1
PUM1	0,070995	0,356353	0,253	0,128	1
IRF7	0,072274	0,896648	0,27	0,149	1
SLC25A24	0,074145	0,433907	0,219	0,106	1
TXNDC12	0,074388	0,549129	0,242	0,128	1
ELOVL1	0,074556	0,336471	0,292	0,17	1
NDUFB6	0,075278	0,579188	0,36	0,234	1
BCKDK	0,075384	0,319961	0,298	0,17	1
MRPS28	0,07672	0,341383	0,163	0,064	1
SUCLG2	0,077117	0,322978	0,191	0,085	1

SLU7	0,077117	0,322978	0,191	0,085	1
TATDN1	0,077707	0,483814	0,163	0,064	1
ZBTB10	0,078208	0,369624	0,101	0,021	1
C12orf45	0,07862	0,250175	0,101	0,021	1
CEP63	0,078693	0,307189	0,163	0,064	1
SNRPN	0,079042	0,283484	0,101	0,021	1
CHD3	0,079042	0,283484	0,101	0,021	1
LGALS2	0,079203	-1,02799	0,517	0,362	1
MRPL37	0,079556	0,289268	0,191	0,085	1
RABIF	0,079685	0,289643	0,163	0,064	1
CHPT1	0,080153	0,298437	0,247	0,128	1
RHBDD2	0,08079	0,393198	0,191	0,085	1
FKBP15	0,080991	0,314562	0,242	0,128	1
HRH2	0,081131	0,351244	0,343	0,213	1
SLC16A7	0,081682	0,253601	0,163	0,064	1
NDUFS5	0,081811	2,182214	0,596	0,489	1
GLS	0,081936	0,452601	0,185	0,085	1
RASGRP2	0,082153	1,003738	0,253	0,128	1
SMNDC1	0,0827	0,254365	0,242	0,128	1
SMARCA2	0,083238	0,347523	0,213	0,106	1
CERS2	0,083243	0,389242	0,213	0,106	1
CD47	0,084535	0,56448	0,371	0,255	1
IRF2	0,084588	0,261743	0,275	0,149	1
FEZ2	0,084798	0,517548	0,213	0,106	1
ETNK1	0,085293	0,458761	0,247	0,128	1
ANKRD44	0,085694	1,034401	0,309	0,191	1
RPL7	0,085877	-2,34917	0,972	1	1
IL10	0,086023	0,384017	0,101	0,021	1
PANK2	0,086358	0,2707	0,213	0,106	1
DPM1	0,086362	0,315677	0,213	0,106	1
MRPL47	0,086803	0,746233	0,258	0,149	1
LMO2	0,087949	0,299365	0,213	0,106	1
CD81	0,088944	0,301543	0,129	0,043	1
CYTH4	0,089244	0,51904	0,287	0,17	1
TCF7L2	0,090093	1,292597	0,157	0,064	1
CHMP2B	0,091028	0,362155	0,258	0,149	1
DNASE1L1	0,091224	0,270231	0,157	0,064	1
ADH5	0,09151	0,252851	0,185	0,085	1
OAT	0,091513	0,300046	0,185	0,085	1
ZMYM2	0,091979	0,281236	0,236	0,128	1
VPS37C	0,092221	0,278088	0,129	0,043	1
MYOF	0,09238	0,347255	0,157	0,064	1
IMP3	0,092659	0,329913	0,264	0,149	1
ISG20	0,094401	0,989847	0,236	0,128	1
NBR1	0,09469	0,265134	0,157	0,064	1
ECH1	0,094851	0,966558	0,393	0,255	1
HSPB1	0,094932	23,79654	0,382	0,277	1
G3BP1	0,095396	0,291057	0,258	0,149	1
CPSF6	0,096208	0,266847	0,213	0,106	1
PARL	0,097636	0,275316	0,258	0,149	1

MAPK1	0,09771	0,470065	0,326	0,213	1
PPP1R10	0,099869	0,369419	0,236	0,128	1
PLXNC1	0,104004	0,32133	0,208	0,106	1
ZNHIT1	0,104851	0,255162	0,534	0,447	1
SLC9A3R1	0,104877	0,475448	0,298	0,191	1
RPS27L	0,105377	1,169227	0,635	0,489	1
CTR9	0,105575	0,281574	0,152	0,064	1
YDJC	0,10592	0,276491	0,124	0,043	1
HSDL2	0,105921	0,325674	0,124	0,043	1
SNW1	0,106419	0,360358	0,23	0,128	1
PARP9	0,106676	0,293787	0,18	0,085	1
C19orf24	0,107805	0,296292	0,264	0,149	1
B3GNT2	0,107841	0,37175	0,124	0,043	1
ATF5	0,107905	0,788081	0,208	0,106	1
NDUFB5	0,108148	0,969858	0,478	0,34	1
ARHGAP17	0,108302	0,276575	0,18	0,085	1
KIDINS220	0,108302	0,276575	0,18	0,085	1
MRPL14	0,108932	0,302888	0,303	0,191	1
HCCS	0,109577	0,324133	0,152	0,064	1
ZNF451	0,110918	0,258438	0,152	0,064	1
MCRS1	0,110918	0,258438	0,152	0,064	1
UBE2E2	0,11426	0,432416	0,202	0,106	1
AIMP1	0,118067	0,593599	0,354	0,255	1
TMEM107	0,118574	-0,69448	0,219	0,319	1
ATP6V1H	0,120331	0,328547	0,225	0,128	1
GCH1	0,120486	0,315394	0,202	0,106	1
GATAD2A	0,122217	0,257538	0,174	0,085	1
RETN	0,123964	6,365623	0,281	0,17	1
LLPH	0,124605	0,38248	0,27	0,17	1
SIAH2	0,124816	0,269646	0,118	0,043	1
FBXO38	0,124816	0,269646	0,118	0,043	1
USP1	0,125039	0,256863	0,146	0,064	1
PRMT1	0,125285	1,859873	0,225	0,128	1
CXXC5	0,125495	0,283022	0,152	0,064	1
GIMAP7	0,125724	1,333259	0,281	0,17	1
SNX30	0,125939	0,250796	0,118	0,043	1
FUOM	0,126057	0,796281	0,337	0,213	1
SLC25A11	0,12739	0,357346	0,208	0,106	1
NOD2	0,127814	0,252541	0,174	0,085	1
SPOPL	0,127814	0,252541	0,174	0,085	1
GTPBP4	0,128127	0,317822	0,146	0,064	1
GBA	0,129676	0,251697	0,146	0,064	1
CCDC47	0,129846	0,372514	0,197	0,106	1
ESD	0,132806	0,346126	0,354	0,234	1
MRPL55	0,135645	0,333719	0,32	0,213	1
SIL1	0,137699	0,417255	0,169	0,085	1
UBE2K	0,140415	0,760747	0,27	0,17	1
RNF5	0,141305	0,263145	0,219	0,128	1
MTHFR	0,141868	0,343431	0,169	0,085	1
PSMD14	0,143976	0,281152	0,169	0,085	1

SSBP4	0,143979	0,442143	0,169	0,085	1
UBASH3B	0,145579	0,28138	0,112	0,043	1
ALDH1A1	0,14689	0,478791	0,112	0,043	1
MAFG	0,148249	0,645969	0,169	0,085	1
LSM5	0,149106	0,593012	0,388	0,255	1
ARMCX3	0,152455	0,262689	0,191	0,106	1
LRG1	0,152462	0,308019	0,191	0,106	1
BNIP3	0,152757	-0,35643	0,051	0,106	1
NSMCE1	0,154841	0,352405	0,242	0,149	1
UBALD2	0,154966	0,362137	0,258	0,17	1
PDXK	0,155113	0,803167	0,191	0,106	1
MRPL16	0,157778	0,274868	0,191	0,106	1
ANKRD13A	0,159056	0,273547	0,213	0,128	1
LAMP2	0,159091	0,391307	0,376	0,277	1
ARRDC2	0,160475	0,257871	0,191	0,106	1
LNPEP	0,162283	0,325543	0,236	0,149	1
USF2	0,163071	0,400365	0,219	0,128	1
BORCS7	0,164614	0,273926	0,287	0,191	1
THUMPD1	0,166689	0,320968	0,163	0,085	1
MDH1	0,168817	0,293896	0,354	0,255	1
GTF2I	0,168919	0,259859	0,287	0,191	1
IFI6	0,169748	5,885998	0,315	0,213	1
EIF1AY	0,173574	0,445736	0,281	0,191	1
OSTM1	0,176197	0,367096	0,135	0,064	1
ZEB2-AS1	0,180326	0,296457	0,191	0,106	1
UBE2E1	0,189126	0,423034	0,23	0,149	1
FAM91A1	0,193141	0,289164	0,23	0,149	1
VMP1	0,193505	6,005943	0,747	0,723	1
RGS18	0,1953	0,488608	0,292	0,213	1
NFATC3	0,19803	0,3585	0,157	0,085	1
GIMAP8	0,200095	0,268043	0,129	0,064	1
FAM50A	0,201062	0,418147	0,264	0,17	1
ZNF703	0,204736	2,733432	0,18	0,106	1
PYM1	0,204736	0,279311	0,18	0,106	1
TIMM13	0,206772	0,35805	0,427	0,34	1
ZDHHC12	0,207106	0,262934	0,129	0,064	1
CCNDBP1	0,217433	0,339834	0,309	0,234	1
CBR1	0,224647	0,269918	0,208	0,128	1
CEBPD	0,226053	5,214514	0,815	0,766	1
TARDBP	0,226823	0,306328	0,174	0,106	1
EIF5B	0,228798	0,381598	0,281	0,213	1
PSMA4	0,230916	0,426552	0,461	0,383	1
CD84	0,234294	0,27312	0,174	0,106	1
ACIN1	0,23808	0,375555	0,174	0,106	1
MT2A	0,238394	96,66827	0,697	0,638	1
NDUFA2	0,242228	0,768946	0,646	0,66	1
KIAA1143	0,243339	0,301127	0,152	0,085	1
GBP4	0,244674	1,212821	0,146	0,085	1
MPST	0,244697	0,290566	0,287	0,213	1
AHCYL1	0,245674	0,267218	0,197	0,128	1

APOL6	0,250604	0,252612	0,27	0,191	1
SUCLG1	0,257924	-1,27345	0,303	0,383	1
RPL27A	0,262345	9,661087	0,961	0,957	1
TLE4	0,272561	0,33414	0,287	0,213	1
ETFB	0,274446	0,451601	0,388	0,298	1
IFI30	0,274601	1,912183	0,77	0,681	1
CISD2	0,275866	0,279018	0,169	0,106	1
MT1X	0,2759	-7,50536	0,354	0,383	1
SDF2L1	0,278626	1,816801	0,242	0,17	1
ADAP2	0,285913	0,342588	0,236	0,17	1
TRIM56	0,296164	0,399472	0,14	0,085	1
IFITM3	0,297995	23,22083	0,691	0,702	1
ASCC3	0,300078	0,383999	0,14	0,085	1
OAZ2	0,309865	0,290626	0,281	0,213	1
MSR1	0,310432	7,714673	0,112	0,064	1
CR1	0,312299	0,619163	0,112	0,17	1
C12orf57	0,313621	0,891521	0,275	0,213	1
RPS20	0,313667	1,775851	0,938	0,809	1
ARF3	0,31418	0,646345	0,337	0,277	1
LRRC8C	0,320611	1,147758	0,112	0,064	1
TM2D3	0,32062	0,26047	0,23	0,17	1
RARRES3	0,327992	1,345374	0,275	0,213	1
EIF6	0,332578	0,343776	0,331	0,277	1
IL32	0,333412	3,709956	0,079	0,128	1
ECHS1	0,334159	0,348335	0,298	0,234	1
PACSIN2	0,342852	0,255083	0,135	0,085	1
IGFBP7	0,369904	-0,34319	0,258	0,191	1
FPR2	0,382081	0,383086	0,157	0,106	1
ODC1	0,396451	0,932732	0,152	0,106	1
PLAC8	0,400635	-2,16289	0,455	0,34	1
TMEM71	0,406075	0,275874	0,219	0,17	1
KLHL5	0,423984	0,313126	0,101	0,064	1
SRSF9	0,426542	-0,96796	0,388	0,277	1
NDUFB7	0,432081	0,641475	0,573	0,532	1
TMEM41B	0,432553	0,277935	0,101	0,064	1
NAA38	0,443192	1,130506	0,522	0,532	1
HCFC1R1	0,443603	0,279735	0,236	0,191	1
NENF	0,45443	-0,27793	0,152	0,191	1
ATOX1	0,45819	0,942547	0,511	0,468	1
CCDC107	0,458434	0,662655	0,169	0,128	1
NCOA7	0,471437	0,876029	0,124	0,085	1
ISCU	0,47168	0,487644	0,348	0,298	1
ACAT1	0,480153	-0,25487	0,118	0,149	1
RAB13	0,496773	0,418433	0,118	0,085	1
STAT1	0,511881	5,784316	0,36	0,298	1
PEBP1	0,516999	-0,69599	0,298	0,34	1
MICU1	0,529627	0,276184	0,14	0,106	1
CLTB	0,542212	0,691315	0,309	0,277	1
VNN2	0,55015	0,370128	0,157	0,128	1
BIN1	0,573658	0,293352	0,112	0,085	1

PIM1	0,585581	0,327553	0,152	0,128	1
IFI44L	0,605353	0,388015	0,129	0,106	1
JOSD2	0,612432	-0,54946	0,213	0,17	1
EPSTI1	0,682136	0,674176	0,213	0,191	1
UQCC2	0,69919	0,344904	0,236	0,213	1
ERAP2	0,758203	0,261551	0,202	0,191	1
DAB2	0,760503	0,537908	0,09	0,106	1
VAMP5	0,772756	8,5334	0,539	0,553	1
POLR2F	0,777858	-0,28058	0,258	0,234	1
MTRNR2L1	0,827033	-0,78592	0,174	0,149	1
CYBA	0,836754	1,082825	0,983	1	1
TNNT1	0,883326	0,260996	0,096	0,106	1
NDUFB1	0,919623	0,393377	0,697	0,702	1
TTC1	0,934459	-0,61931	0,157	0,149	1
ISG15	0,951911	-12,2745	0,331	0,34	1
